# Elucidation of DNA Primase as a Drug Target in *Leishmania donovani*: Structure‐Guided Inhibitor Identification and Biochemical Validation

**DOI:** 10.1111/cbdd.70362

**Published:** 2026-07-25

**Authors:** Deep Bhowmik, Anupama Sharma, Ravi Prakash Arya, Diwakar Kumar

**Affiliations:** ^1^ Department of Microbiology Assam University Silchar Assam India; ^2^ Department of Veterinary Microbiology Assam Veterinary and Fishery University Khanapara, Guwahati India; ^3^ Center for Computational Natural Sciences and Bioinformatics International Institute of Information Technology Hyderabad India; ^4^ Department of Biotechnology Assam University Silchar Assam India

**Keywords:** DNA primase, enzyme inhibition, inhibitors, leishmaniasis, MTT assays, primase‐pyrophosphatase assay

## Abstract

Replication is started by DNA primase, which synthesizes an oligoribonucleotide primer that DNA polymerases extend. Effective chemotherapy is still needed to manage and treat leishmaniasis, which continues to pose a threat to public health around the world. Repurposing drugs offers alternative uses for drugs with established pharmacological effects, saving money and increasing the pool of human resources available to create novel anti‐leishmanials. Here, we used a non‐radioactive primase‐pyrophosphatase assay to biochemically characterize the *Leishmania donovani* nuclear DNA primase (*LdPri*) subunits, followed by in silico evaluation of inhibitors targeting *LdPri*. The best‐evaluated inhibitors showed *LdPri* inhibition in vitro under conditions similar to those of the primase‐pyrophosphatase experiment. The MTT assay also confirmed the inhibitors' anti‐leishmanial properties. Parasite growth and morphological analysis were performed by culturing cells in the presence of inhibitors. Biochemical characterization revealed that *LdPriS* activity was unstable, but was highly stabilized upon association with *LdPriL* in *LdPri*. *LdPri* was found to be thermostable and possesses 3′‐terminal nucleotidyltransferase activity. Additionally, in silico and in vitro studies evaluated Pritelivir (BAY 57‐1293) as the most efficient *LdPri* complex inhibitor, followed by Epigallocatechin Gallate (EGCG). Moreover, kinetic studies showed that Pritelivir and EGCG exhibit competitive and uncompetitive inhibition, respectively, for both NTP and DNA substrates. Pritelivir effectively disrupted the *L. donovani* cell cycle, and the replication mechanism in treated promastigotes showed irregular shapes and short flagella.

## Graphical abstract

Structure‐guided and biochemical analyses indicate that DNA primase of *Leishmania donovani* represents a potential therapeutic target, as repurposed compounds, particularly Pritelivir, demonstrate marked inhibitory effects accompanied by reduced parasite proliferation, replication, and altered cellular morphology in in vitro and cell‐based studies.

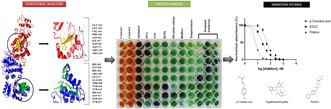

## 1 Introduction

Visceral leishmaniasis (VL) is a potentially fatal vector‐borne disease, ranking second and seventh among tropical diseases in mortality and disability‐adjusted life years, respectively (Wang et al. 2016). However, VL is classified as one of “the neglected diseases” because it is often seen as a disease of developing nations, and the pharmaceutical sector shows little interest in funding research into it (Yamey and Torreele 2002; Pacheco‐Fernandez et al. 2021). With an estimated 200 million individuals at risk, VL is endemic in more than 70 nations on every continent, except Antarctica and Australia. However, more than 90% of VL cases worldwide are documented in seven countries: Brazil, Ethiopia, India, Kenya, Somalia, South Sudan, and Sudan (Burza et al. 2018). Each year, there are an estimated 500,000 new cases of VL and 50,000 fatalities, both of which are believed to be underestimated (Murray 2002; van Griensven and Diro 2012).

The current control of visceral leishmaniasis (VL) is entirely based upon chemotherapy, as a competent vaccine is still a challenge. Currently, the challenges in chemotherapy include resistance, toxicity, the high cost of treatment, and the availability of very few drugs. Chemotherapeutics such as pentavalent antimonials, amphotericin B, miltefosine, and paromomycin have limited efficacy. Novel drugs and drug targets are of utmost importance for VL treatment (Chawla and Madhubala 2010). Generally, drug target identification from biological pathways follows two rules. First, to identify a putative target, either absent or different in the host in structural and functional form. Secondly, identifying targets required for essential biosynthesis and survivability, and those that are easily assayable for high‐throughput screening (HTS) (Raj et al. 2020).

Repurposing medications has already become a popular alternative to prescription drugs for a range of illnesses. Repurposing drugs involves using existing drugs with well‐established pharmacological effects for alternative uses, saving money and expanding the pool of human resources available to create novel anti‐leishmanials (Bustamante et al. 2019; de Souza et al. 2020). Several alternative options with effective anti‐leishmanial properties in vitro and in vivo have been identified previously, even though the practice of repurposing medicines with other approved uses and demonstrating their anti‐leishmanial activity is relatively recent (Bustamante et al. 2019). There have been several new initiatives to repurpose medications for neglected tropical diseases (Roatt et al. 2020).

DNA polymerases' trademark is their inefficiency in de novo DNA synthesis. DNA primases are crucial for DNA replication, catalyzing the synthesis of a short RNA primer on single‐stranded DNA (ssDNA) templates and then transferring the primer to DNA polymerases to initiate DNA synthesis (Kuchta and Stengel 2010). DNA primase also monitors cell division checkpoints and is crucial for DNA repair processes (Michael et al. 2000; Jozwiakowski et al. 2015; Xiong et al. 2025).

The DNA primase complex is found in all eukaryotic species studied to date (Guilliam et al. 2015). It consists of two subunits: a large accessory subunit of approximately 58 kDa and a small catalytic subunit of approximately 49 kDa. These subunits are associated with the 180 kDa DNA polymerase α (Baranovskiy et al. 2016). Large accessory subunit (p58) residues from both its N‐ and C‐terminal halves are involved in complex formation and can signal to drive the primase complex and itself to the nucleus (Copeland 1997). The small subunit has a well‐adapted RNA recognition motif, which is also present in Superfamilies A, B, and Y of DNA polymerases, cyclases, viral reverse transcriptase, and RNA‐dependent RNA polymerases (Iyer et al. 2005). In contrast, the large subunit has two primarily α‐helical domains plus a C‐terminal 4Fe–4S cluster (O'Brien et al. 2017).

Previous research has already discovered the Prim‐Pol genes in eukaryotes, including rats, humans, and yeasts (Frick and Richardson 2001). It was shown that the small component of DNA primase (PriS) could synthesize RNA primers, but that its free form was unstable and its activity was lower than that of the primase complex (Arezi et al. 1999). Though its exact function is still unknown, the large subunit (PriL) was able to bind ssDNA at the replication fork, stabilize the small subunit (PriS) in the primase complex, and participate in the start of primer synthesis, improvement of primase processivity, persistence of product size, and transfer products to DNA Pol α (Arezi et al. 1999).

Information regarding the role of DNA primase in DNA replication is limited in *Leishmania* spp. Primase synthesizes short RNA primers that DNA polymerases use to initiate DNA replication. Without primase, cells cannot duplicate their genome, making it indispensable for cell division. Primase belongs to the replication machinery, a class of enzymes already targeted by antiviral and anticancer drugs. Purification and biochemical characterization of these protein subunits have not been reported previously in *Leishmania* and other kinetoplastids. Despite the few similar sequences, many conserved motifs are present in all known eukaryotic primase sequences. Our laboratory experimentation included the isolation, purification, and biochemical characterization of *L. donovani* nuclear DNA primase (*LdPri*) and its subunits, large (*LdPriL*) and small (*LdPriS*), by a non‐radiolabelled primase‐pyrophosphatase assay. *LdPriL* and *LdPriS*, both subunits, possess primase activity; however, *LdPriS* activity was low and stabilized upon association with *LdPriL*. *LdPri* activity was assessed, analyzed, and interpreted by optimizing various parameters in the primase assay. Herein, we have characterized nuclear DNA primase in *L. donovani* and used it as a drug target to develop a time‐ and cost‐effective drug‐repurposing approach for VL treatment. A comprehensive perspective on the potential of *LdPriL* and *LdPriS* within the replication machinery of the *L. donovani* parasite, and on their inhibition, would be beneficial for VL treatment and eradication.

## 2 Materials and Methods

### 2.1 Sequence Retrieval and BlastP Analysis

The individual amino acid sequences of *L. donovani* nuclear DNA primase large subunit (*LdPriL*) (LDBPK_030080) and small subunit (*LdPriS*) (LDBPK_220270) were saved from the KEGG database (https://www.genome.jp/kegg‐bin/show_organism?org=ldo) in FASTA format. The sequences were cross‐validated against other databases, such as UniProt (accession no. E9B7M6) and NCBI (accession no. XP_003857974) for the large subunit, and UniProt (accession no. E9BFP6) and NCBI (accession no. XP_003860777) for the small subunit, respectively. The protein sequences of both subunits were submitted to BLASTP for homology searches against the human primase database (https://blast.ncbi.nlm.nih.gov/Blast.cgi?PAGE=Proteins), large (accession no. NP_000938) and small (accession no. NP_000937) subunits, respectively.

### 2.2 Homology Modeling and Model Validation

Homology modeling is a robust methodology with multiple easy‐to‐follow steps for analyzing a protein's three‐dimensional structure from its amino acid sequence. Sequence similarity between a known protein structure (template) and the protein to be modeled (target) significantly impacts the quality of predicted models and their application in drug design and discovery (Hillisch et al. 2004).

MODWEB (http://salilab.org/modweb) (Sánchez and Sali 1998; Vani et al. 2020) and Modeler version 9.20 (http://salilab.org/modeller) (Šali et al. 1995; Webb and Sali 2016) were used for homology modeling. Structurally reliable models for *LdPriL* were better with MODWEB, whereas *LdPriS* models were built with Modeler version 9.20.

The absence of a resolved, crystallized structure for *L. donovani* nuclear DNA primase prompted the construction of models for both subunits by comparing primary sequences with the nearest known structure. MODWEB performs comparative modeling for unknown protein structures. The input to MODWEB is the FASTA amino acid sequence, which is then used to generate models from the ideal template already deposited in the Protein Data Bank (PDB) (http://www.rcsb.org) (Eswar et al. 2003). The model with the highest ModPipe Protein Quality Score (MPQS) (> 1.1) and the highest sequence identity per region is considered reliable and selected for further docking studies (Shen and Sali 2006).

The BLAST tool (Ye et al. 2006) was used to select templates for homology modeling *of LdPriS* by querying protein repositories. The catalytic subunit of human primase (PDB: 4LIK) was chosen as a template because BLASTP revealed 34% identity and 72% sequence overlap with *LdPriS*. The template protein's PDB file was subsequently downloaded. The protein models were created using Modeler 9.20 (http://salilab.org/modeller), which requires a sequence alignment, template structures, the arrangement of atoms, and a short script file (Eswar et al. 2003).

PROCHECK's Ramachandran plot analysis validated all models built using MODWEB and Modeler (Laskowski et al. 1993; Colovos and Yeates 1993; Bhardwaj et al. 2024). Finally, the best model for each *L. donovani* nuclear DNA primase subunit was submitted to the YASARA Energy Minimization Server (Krieger et al. 2009; Nezhad et al. 2025) to obtain a low‐energy, highly stable conformation. The structural integrity and authenticity of the energy‐minimized models were investigated using ERRAT and ProSa, as well as (Colovos and Yeates 1993; Wiederstein and Sippl 2007; Nezhad et al. 2025).

### 2.3 Protein–Protein Docking and Interaction Analysis Between the *L. donovani* Nuclear DNA Primase Large Subunit ( *LdPriL* ) and Small Subunit ( *LdPriS* )

Small‐molecule protein inhibition has thrived since the mid‐1970s; however, efficacy has been declining due to limited space on the target for drug binding. However, protein–protein interactions (PPIs), which play a critical role in disease‐related metabolic pathways, are emerging as a promising target space for drug delivery and discovery (Macalino et al. 2018).

Protein–protein docking between the *LdPriL*and *LdPriS* has been carried out using an easy interface with the HADDOCK web server. The SPPIDER web server (Solvent accessibility‐based Protein–Protein Interface iDEntification and Recognition) was used to determine the active amino acid residues in protein–protein interactions for *LdPriL* and *LdPriS* (Table S1) (Porollo and Meller 2007), and the results were uploaded to HADDOCK 2.4 for analysis against the appropriate proteins. The interactions between *the LdPriL* and *LdPriS* to form the *LdPri* complex were analyzed by PDBsum (http://www.ebi.ac.uk/thornton‐srv/databases/pdbsum/Generate.html). Gibbs free energy (ΔG) and dissociation constant (*Kd*) values of *LdPri* were predicted utilizing the PRODIGY server (Xue et al. 2016).

### 2.4 Protein Preparation and Active Site Determination of *LdPri* Complex

The HADDOCK‐generated *LdPri* complex was subjected to energy minimization in YASARA (Krieger et al. 2009) to obtain the low‐energy, most stable conformation.

The active site of *LdPriL* was identified following a thorough study and multiple sequence alignment (MSA) with the large subunit of other eukaryotes. Sequence conservation and architectural similarity of the active site at the N‐terminus of the yeast *PriL*‐Carboxy‐Terminal Domain (*PriL*‐CTD), which possesses substrate‐binding sites and DNA photolyase/cryptochrome activity, provide promising insight into the pivotal role of *PriL*‐CTD in the replication machinery (Pokorny et al. 2008). The novel architecture of yeast *PriL*, as revealed by its crystallized structure, explains several biochemical and genetic observations. It sheds light on the mechanism of short RNA primer synthesis during eukaryotic replication (Sauguet et al. 2010). Evolutionary studies of *PriL*‐CTD identified residues (334–423) that constitute the active site and perform essential functional roles. MSA through Clustal Omega (Sievers and Higgins 2018) confirmed the conservation of these critical amino acid residues in *LdPriL* (Figure S1A). The yeast *PriL*‐CTD active site residues with their essential functions in yeast replication machinery are shown in Table S2.

For active site determination of *LdPriS*, the literature survey and MSA provided the required insights. An earlier investigation of the alanine alteration in the mouse catalytic DNA primase subunit showed that residues 104–111 are necessary for the generation of primers, with GLU 105 located close to the adjacent chains of two more critical residues, ASP 109 and ASP 111, that are located within the active site region (Bhowmik et al. 2021). Clustal Omega was used to align DNA primase protein sequences from different species with the sequence of our modeled *LdPriS* to identify essential residues and regions that might form our modeled *LdPriS*'s binding cavity. Sequence alignment confirmed the amino acid residues (ELFVDID) conservation among the eukaryotes (Bhowmik et al. 2021). Mutated ARG 162 and ARG 163 in the mouse catalytic subunit are associated with nucleotide binding (Copeland and Tan 1995) and were also found to be aligned with *LdPriS* residues, with the latter arginine residue found to be conserved across every other eukaryote. ASP 114 in mice was related to the recognition of the 5′ purine residue of the template and was also found to be conserved in *LdPriS* (Figure S1B).

### 2.5 *In Silico* Alanine Scanning Mutagenesis (ASM)

Identification of hotspot residues at the target protein's active site was achieved using *in silico* alanine‐scanning mutagenesis (ASM) via the PPCheck web server (David and Sternberg 2015; Sukhwal and Sowdhamini 2015). A mutagenesis study reveals the crucial residues that confer functionality and stability to the protein under study (Hafiz et al. 2022). The active site identified in MSA studies for both *LdPriL* and *LdPriS* was selected for hotspot residue identification by performing mutagenesis experiments to assess the importance of individual residues in the protein that interact with higher free energy (Sharma et al. 2022).

### 2.6 Ligand File Preparation

A total of 165 natural anti‐leishmanial compounds without any specific target against *L. donovani* were selected from earlier literature studies (Mishra et al. 2009; Rodrigues et al. 2015; Antwi et al. 2019; Gervazoni et al. 2020). Moreover, 124 synthetic DNA/RNA synthesis inhibitors and 409 protein–protein interaction (PPIs) inhibitors from Selleckchem.com (https://www.selleckchem.com/) were considered for docking purposes against *the LdPri* complex. Canonical SMILES (Simplified molecular‐input line‐entry system) string of all individual ligands from PubChem (Kim et al. 2019) were converted into Protein Data Bank (PDB) format using Chimera 1.14 (Goddard and Ferrin 2007) according to our docking protocol.

### 2.7 Molecular Docking and Interaction Analysis

Molecular docking between the prepared ligands against *LdPriL* and *LdPriS* individual proteins, followed by docking against *LdPriL* and *LdPriS* in *the LdPri* complex, was done through PyRx virtual screening software, which utilizes AutoDock 4 and AutoDockVina for docking purposes (Dallakyan and Olson 2015). The macromolecular structure of *LdPriL*, *LdPiS*, and *LdPri* complex, as well as the ligands, were prepared, and hotspot residues were prepared inside a grid box for induced fit docking with X, Y, and Z axes and dimensions adjusted to 25.41 Å *×* 28.28 Å *×* 19.15 Å for *LdPriL* and 25.44 Å *×* 11.09 Å *×* 20.28 Å for *LdPriS*.

Molecular docking against residues involved in H‐bond formation between *LdPriL* and *LdPriS* in the *LdPri* complex was also considered a hotspot in PyRx. Residues were within the grid box, and the dimensions were adjusted to 23.19 Å × 53.36 Å × 30.09 Å for identifying potential PPI inhibitors.

Using Ligplot (Wallace et al. 1995) and Pymol Molecular Visualization Software (Lill and Danielson 2011), the interaction between the ligands and the hotspot residues between *LdPriL* and *LdPriS* in the *LdPri* complex was investigated, and the 3D pictures were made using Pymol Molecular Visualization Software. Molecular dynamics (MD) simulation studies further evaluated the ligands with the lowest docked energies and efficiencies at all the considered hotspot sites and interacting residues in the *LdPri* complex.

Molecular docking between Epigallocatechin Gallate (EGCG), p‐Coumaric acid (4‐Hydroxycinnamic acid), and Pritelivir (BAY 57‐1293) was also done against both the large and small subunits of the energy minimized Human DNA Primase (PDB ID: 4RR2) through PyRx virtual screening software for docking purposes (Dallakyan and Olson 2015).

### 2.8 Molecular Dynamics (MD) Simulation

Molecular dynamics simulations are performed to validate the docking complex and assess stability and residual effects after docking. *LdPri* complex with its top binding ligands bound to the hotspot residues of *LdPriL* in the *LdPri* complex (**Set 1**), *LdPriS* in the *LdPri* complex (**Set 2**), as well as the interacting site between *LdPriL* and *LdPriS* in the *LdPri* complex (**Set 3**), along with the unbound *LdPri* complex without any inhibitor.

Stability of protein‐ligand complexes was studied by running the above three sets (**Set 1–3**) of molecular dynamics simulations experiments in GROMACS2016.3 (Abraham et al. 2015) utilizing CUDA acceleration and amber99sb‐ildn force field (Lindorff‐Larsen et al. 2010) to model the protein for the repurposing as well as to demonstrate the potential binding mode of the selected inhibitors. The topology receptors were retrieved using *pdb2gmx*. The general Amber force field (Gaff) (Wang et al. 2004) for the ligand parameters under study was generated using the Amber tools, and the resulting parameters were converted to GROMACS format using the acpype tool (Sousa da Silva and Vranken 2012). The TIP3P water model potential has been used to solvate the protein, with a 1 nm separation between the protein edges in all directions. Adding Na^+^ and Cl^−^ ions neutralized the system with periodic boundary conditions. When utilizing the leapfrog approach to solve the Newton equation of motion, the hydrogen bonds were subjected to the linear constraint solver (LINCS) algorithm (Hess et al. 1997). The simple space cutoff length was adjusted to 12.0 Å, and distant electrostatic forces were computed using the particle mesh Ewald summation (Essmann et al. 1995). To calculate van der Waals interactions, a shifted cutoff of 10.0–12.0 Å was used. System energy was minimized using the steepest‐descent algorithm with a maximum force of 1000 kJ/mol · nm. NVT equilibration was performed by gradually raising the system temperature to 300 K over 100 ps. Following NVT, a 100 ps NPT simulation was performed to obtain the correct density for the system, employing a velocity‐rescale thermostat and a Berendsen barostat with coupling constants of 1.0 and 2.0 ps, respectively. Then, for all of the complexes in **Sets (1–3)** and the unbound *LdPri* complex, 100 ns MD‐simulations under the NPT ensemble (*p* = 1 bar and *T* = 300 K) were carried out. Every 100 ps, trajectories of each system were saved and analyzed. The course was assessed using multiple GROMACS programs and visualized with PyMOL. The VMD tool (Humphrey et al. 1996) generated all the graphs from the analysis of the 100 ns MD simulation.

### 2.9 Essential Dynamics (ED) Study

A suitable analytical approach is essential dynamics (ED) or principal component analysis (PCA) for depicting biomolecule slow and functional movements (David and Jacobs 2014). The principal components for **Sets (1–3)** were derived by diagonalizing the covariance matrices and analyzing their eigenvalues and eigenvectors. While the eigenvalues only indicate the direction of motion, they also indicate the magnitude of motion. Using the GROMACS analysis tool gmx covar, the covariance matrix from the PCA experiment was calculated and diagonalized. The GROMACS analysis tool gmx anaeig was used to determine whether primary components and kinematic dimensions overlapped.

### 2.10 g_mmpbsa Analysis

The molecular mechanics/Poisson‐Boltzmann Surface Area (MM‐PBSA) method estimates the affinity of inhibitors for a protein receptor by combining different energies (Kumari et al. 2014). Typically, these energies generated due to the interaction of receptor‐ligand complexes were calculated using g_mmpbsa in conjunction with MD modeling using the equation:

(1) $$\Delta G_{\text{binding}}=\Delta G_{\text{complex}}–\left(\Delta G_{\text{protein}}+\Delta G_{\text{ligand}}\right)$$

Additionally, the specific free energy of a receptor‐inhibitor complex can be determined using Equation (2).

(2) $$\Delta G=\left(E_{\mathrm{MM}}\right)+G_{\text{solvation}}$$

where (*E*_MM_) is the molecular mechanics average potential energy, ignoring pressure. There are two components to the average free solvation energy: polar and non‐polar.

(3) $$\Delta G_{\text{solvation}}=\Delta G_{\text{polar}}+\Delta G_{\text{nonpolar}}$$

This method requires three stages for binding free energy calculation. Calculating the potential energy in the vacuum is the first step. Estimation of the polar and non‐polar solvation energies was followed. The solvent‐accessible surface area (SASA) model was used to determine the nonpolar solvation energy. The final 1000 frames from each trajectory were selected for calculating the binding free energy.

### 2.11 Cloning, Overexpression, and Purification of the *LdPriL* ‐pASK‐IBA43plus and *LdPriS* ‐pET‐28a (+) Construct

*L. donovani* nuclear DNA Primase, which is heterodimeric, was amplified using Phusion DNA polymerase and cloned into the pENTR/D‐TOPO vector. *LdPriL* was further subcloned into the expression vector pASK‐IBA43plus, while *LdPriS* was subcloned into pET −28 a (+) vector for overexpression studies. Recombinant *Escherichia coli* BL21 cells harboring *LdPriL* and *LdPriS* constructs were cultured in antibiotic‐supplemented LB medium and induced at mid‐log phase using anhydrotetracycline or IPTG, followed by incubation at 16°C to enhance soluble protein expression. Harvested cells were lysed using lysozyme‐assisted sonication, and soluble fractions were separated for SDS‐PAGE–based solubility assessment on 12% polyacrylamide gel. Proteins were purified from clarified lysates after lysis with lysis buffer (50 mM NaH_2_PO_4_, 300 mM NaCl, 10 mM imidazole, pH 8) via cobalt‐based immobilized metal affinity chromatography using TALON resin chelated with Co^2+^ ions specific for His‐tag purification, with stepwise imidazole elution (50 mM NaH_2_PO_4_, 300 mM NaCl, 250 mM imidazole, pH 8). Eluted proteins were desalted using Pierce Protein Concentrators with a 10 MWCO (PES, Thermo Scientific), quantified by Bradford assay, and stored in glycerol at −80°C for downstream enzymatic and inhibition studies. The western blot assay confirmed the synthesis of recombinant *LdPriL* and *LdPriS*. Protein samples (0.5, 5, 10, 20, 30, 50, and 100 ng) of both recombinant proteins were electrophoresed in 12% SDS‐PAGE and electroblotted on a 0.45 μm PVDF membrane (Thermo Scientific) in a tank transfer system at 100 V for 1 h at 4°C for blotting. PVDF membranes were blocked with 5% nonfat milk in PBST after protein transfer from 12% SDS‐PAGE. Membranes were washed thrice with PBST for 10 min before incubating for 1 h with a 1:2000 dilution of 6× His‐tagged rabbit Monoclonal antibody (NovagenR) in 5% nonfat milk‐PBST, followed by washes as described above. Following washing, the membranes were treated with a 1:5000 dilution of peroxidase‐conjugated goat antirabbit IgG (Sigma Aldrich) in 5% nonfat milk‐PBST, rewashed with PBST, and revealed using the Amersham enhanced chemiluminescence ECL reagent.

### 2.12 Optimization of Colorimetric Primase‐Pyrophosphatase Assay Conditions and Biochemical Characterization of *LdPri*

Most academic screening facilities are limited to testing for radioactive elements; a novel non‐radioactive primase‐pyrophosphatase (also called primase‐phosphatase) assay with high‐throughput screening (HTS) applications, reported earlier, provided a new, ready‐to‐use approach (Biswas et al. 2013). The primase activity assay was optimized on flat‐bottom 96‐well clear polystyrene plates (Thermo Scientific). 30 μL of reaction mixture contained *LdPri* (0.7 μM both *LdPriL* and *LdPriS* in 1:1 M ratio), M13mp18 ss DNA (1.25 μM or specified), dNTPs (100 μM or as specified), buffer (20 mM of CAPS pH 8.8 or specified), divalent metal ions (4 mM Mg^2+^ or as specified), 50 mM NaCl, potassium glutamate (KGlu) (150 mM or as specified) and 1 U pyrophosphatase (PPiase). Reactions were carried out at 22°C in an incubator for 30 min. After incubation, 90 μL malachite green reagent (MGR) was added, followed by 30 μL of 10% sodium citrate, which was added after 1 min to end the reaction, and the absorbance at 620 nm was measured in a microplate reader (Biswas et al. 2013; Rai et al. 2021). A primase‐pyrophosphatase assay for *LdPriL* and *LdPriS* was also considered to measure their primase activities individually.

- *Effect of pH*: *LdPri* activity on pH dependency was determined by primase‐pyrophosphatase assay at 22°C in different buffer solutions with varying pH. The buffer systems for optimization included 20 mM CAPS–NaOH (pH 9.9, 9.0, and 8.5); 50 mM Tris–HCl (pH 8.5, 7.5, and 6.8); 20 mM HEPES–NaOH (pH 7.5 and 6.5); and 20 mM MOPS–NaOH (pH 6.5 and 6.0).
- *Thermostability test*: The thermostability of the *LdPri* activity was measured by incubating *LdPri* (*LdPri* and *LdPriS* in a 1:1 M ratio) in Buffer (Tris 7.5, NaCl 300 mM, and 20% glycerol) at 40°C, 50°C, 60°C, 70°C, and 80°C for 15 min, and then checking for efficient primase activity. The samples were centrifuged at maximum speed to remove debris and any precipitate. The supernatant was assayed using M13mp18 ssDNA as the template for 30 min at 22°C.
- *Terminal nucleotidyltransferase activity assay*: The *LdPri* terminal transferase activity was carried out in the same way as the primase activity assay. The 30 μL primase‐pyrophosphatase reaction mixtures with random oligonucleotides including M13mp18ssDNA, a 34‐mer primer (5′CACCGAATTCCATATGCAAGCCATCACCGCCTCT 3′), an 18 nucleotides long Oligo (dT) and with individual dNTPs (dATP, dCTP, dGTP, dTTP) were used for analyzing the efficiency of *LdPri* activity.

### 2.13 Primase Inhibition Assay

Primase inhibition assay was carried out under similar conditions to the primase‐pyrophosphatase assay, that is, 30 μL of reaction mixture contained *LdPri* (0.7 μM both *LdPriL* and *LdPriS* in a 1:1 M ratio), M13mp18 ss DNA (1.25 μM or specified), dNTPs (100 μM or as specified), buffer (20 mM of CAPS pH 8.8 or specified), divalent metal ions (4 mM Mg^2+^ or as specified), 50 mM NaCl, potassium glutamate (KGlu) (150 mM or as specified), and 1 U pyrophosphatase (PPiase). In this assay, different concentrations of the selected ligands (10 nM, 25 nM, 50 nM, 100 nM, 250 nM, 500 nM, and 1 μM) were used in triplicate, with the primase reaction components kept constant. Absorbance at 620 nm was measured, and the IC_50_ values for the inhibitors were calculated using GraphPad Prism v8.0 (Biswas et al. 2013). Inhibitory studies were also carried out with the same ligand concentration on individual *LdPriL* and *LdPriS* activity.

### 2.14 Enzyme Kinetics and Analysis of the Mode of Inhibition Assays

Enzyme kinetics and inhibition mode were calculated from absorbance measurements of released PPi in 30‐min reactions. The primase‐pyrophosphatase reaction was run at different substrate concentrations: NTP (50, 100, 200, 300, 400, and 500 μM) and DNA (0.5, 1, 1.25, 1.75, and 2 μM). Spectroscopic measurements of the kinetics of polymerization of dNTPs and DNA by *LdPri*, in the absence and presence of inhibitors (IC_50_, 2× IC_50_), were performed at 620 nm for 30 min, with values recorded every 5 min. *V*_max_ and *K*_m_ for the reactions in the presence and absence of inhibitors were calculated from Lineweaver–Burk plots (Biswas et al. 2013; Iqbal et al. 2021). All the experiments were performed in triplicate. Results were evaluated using graphical techniques in GraphPad Prism v8.0 (Biswas et al. 2013).

### 2.15 Parasite Inhibition Assay

Parasite inhibition was assessed using the MTT Cell Growth Assay Kit. *L. donovani* promastigotes were harvested in fresh media M‐199 with 10% Fetal Bovine Serum (FBS) added in the presence and absence of different concentrations of inhibitors (10 μM, 25 μM, 50 μM, 100 μM, 250 μM, 500 μM, and 1 mM) as well as positive control Amphotericin B (10 μM, 25 μM, 50 μM, 100 μM, 250 μM, 500 μM and 1 mM) for 72 h at 25°C in 96 well plates. After incubation and removal of the medium, 5 mg/mL MTT solution in 1× PBS was added to each well for an additional 3 h. Following incubation, the crystallized formazan was solubilized in acidified isopropanol and incubated for an additional 30 min at room temperature. Optical density at 570 nm was measured using a Thermo Scientific microplate reader. Sensitivity was determined in three independent experiments, and data were expressed as the percentage of cell viability using Equation (4).

(4) $$\%\text{Cell viability}=\frac{\text{Mean}\;{\mathrm{OD}}_{570}\;\text{of}\;\text{test}\;\text{samples}}{\text{Mean}\;{\mathrm{OD}}_{570}\;\text{of}\;\text{control}}\times 100$$

### 2.16 Effects of Inhibitors on Parasite Growth and Morphology of *L. donovani* Promastigotes

The dose–response effects of inhibitors on *L. donovani* replication at different inhibitor concentrations (IC_50_, 2× IC_50_) and Amphotericin B (IC_50_) were determined against the untreated control by culturing *L. donovani* promastigotes in fresh M199 medium for 120 h at 25°C. The live parasites were determined by trypan blue staining every 24 h and counting them using a hemocytometer under a phase‐contrast microscope (100×). The total number of parasites per field was determined using ImageJ Software. Three independent experiments were conducted to determine the number of viable cells and the percentage of cell viability with Equations (5) and (6).

(5) $$\begin{array}{c} \text{Total}\;\text{number}\;\text{cells}=\left(\text{Total}\;\text{cells}\;\text{counted}\right)/\left(4\;\text{squares}\;\text{counted}\right)\times 10^{-4}\\ \;\times \text{initial}\;\text{volume}\times \text{dilution}\;\text{factor} \end{array}$$

(6) $$\%\text{Viable cells}=\frac{\text{Total number of viable cells}\;\mathrm{per}\;\text{milliliter of aliquot}}{\text{Total number of cells}\;\mathrm{per}\;\text{milliliter of aliquot}}\times 100.$$

Promastigotes in fresh M199 medium, in the presence of different inhibitor concentrations (IC_50_, 2× IC_50_) and Amphotericin B (IC_50_), or untreated, were cultured for 24 h at 25°C and subjected to low‐speed centrifugation for 20 min to obtain a complete pellet. These pellets were cleaned, resuspended in 1× PBS, and fixed for 10 min in 4% paraformaldehyde (PFA). The morphological differences between the inhibitor‐treated *L. donovani* promastigotes and the untreated culture slides were observed on coverslips mounted with DPX and viewed under a phase‐contrast microscope (100×).

### 2.17 Statistical Analysis

Results from triplicate experimentation were presented as mean ± SD (standard deviation). The IC50 values were determined from in vitro anti‐primase and anti‐leishmanial activity using non‐linear regression analysis in GraphPad Prism 8.0. In Microsoft Excel Version 16.56 and GraphPad Prism 8.0, the Student's *t*‐test and Dunnett's multiple‐comparisons test with *p*‐values < 0.05 were considered significant.

## 3 Results

### 3.1 Sequence Retrieval and BlastP Analysis

KEGG pathway (https://www.genome.jp/kegg‐bin/show_organism?org=ldo) was utilized for retrieving the individual amino acid sequences of the *L. donovani* nuclear DNA primase large subunit and small subunit. It was cross‐validated with other databases, such as UniProt and NCBI. BLASTP against the human DNA primase subunits (https://blast.ncbi.nlm.nih.gov/Blast.cgi?PAGE=Proteins) showed 31.17% and 34.25% similarity with the large and small subunits, respectively, along with low maximum and total scores, indicating poor homology (Table S3).

### 3.2 Homology Modeling and Model Validation

MODWEB generated three models for *LdPriL* based on two templates from PDB: 3LGB (Crystal Structure of the Fe–S Domain of the yeast DNA primase) with sequence identity 33%, and PDB: 4RR2 (Crystal structure of human primase) with sequence identity 29%. The homology models achieved high evaluation scores and were deemed of good quality for *LdPriL*. However, model 2 was selected and considered best for further docking studies, as the MPQS score was 1.2 (> 1.1), GA341 was > 0.7, and the discrete optimized protein energy zDope was < 1 (Altschul et al. 1997; Melo et al. 2002; Shen and Sali 2006). Further, Ramachandran plot analysis using PROCHECK evaluated model 2, which covered more residues (445 out of 501) than model 1, with 98.9% of residues covering favored, additionally and generously permitted regions, and only 1.1% in the disallowed region (Figure S2A). The ERRAT value for the energy‐minimized model was 89.778%, indicating high structural quality (Figure S2B). A Z‐score of −9.5 and energy plots by ProSa indicated an excellent *LdPriL* model (Figure S2C,D).

When *LdPriS* was compared to the protein databank using BLASTP, PDB: 4LIK showed the highest similarity and was selected as the template for model generation. Five *LdPriS* models were generated using Modeler version 9.20 and verified using PROCHECK. Model 4 was chosen for further investigation because it contained 99.5% residues in the favored, additionally allowed, and generously permitted regions and only 0.5% in the banned zone, as shown in Figure S3A. To improve the model's stereochemical properties, energy minimization was performed using the YASARA Energy minimization online tool. The ERRAT value for the energy‐minimized *LdPriS* was 89.756% (Figure S3B). ProSa's energy plots and a z‐score of −5.71 also revealed an overall high‐quality *LdPriS* model (Figure S3C,D).

### 3.3 Protein–Protein Docking and Interaction Analysis Between the *L. donovani* Nuclear DNA Primase Large Subunit ( *LdPriL* ) and Small Subunit ( *LdPriS* )

HADDOCK generated 9 structures in 2 clusters, accounting for 60% of the explicit‐water HADDOCK models. The HADDOCK score of −200.1 ± 26.0 for cluster 1 indicates proper interaction between *LdPriL* and *LdPriS*. The buried surface area (BSA) score of 2100.5 ± 93.6 A^2^ indicated close juxtaposition and hydrophobicity on the protein‐binding surface (Ghosh et al. 2021). The HADDOCK calculated energies and the Z‐Score are listed in Table S4.

**FIGURE 1:**
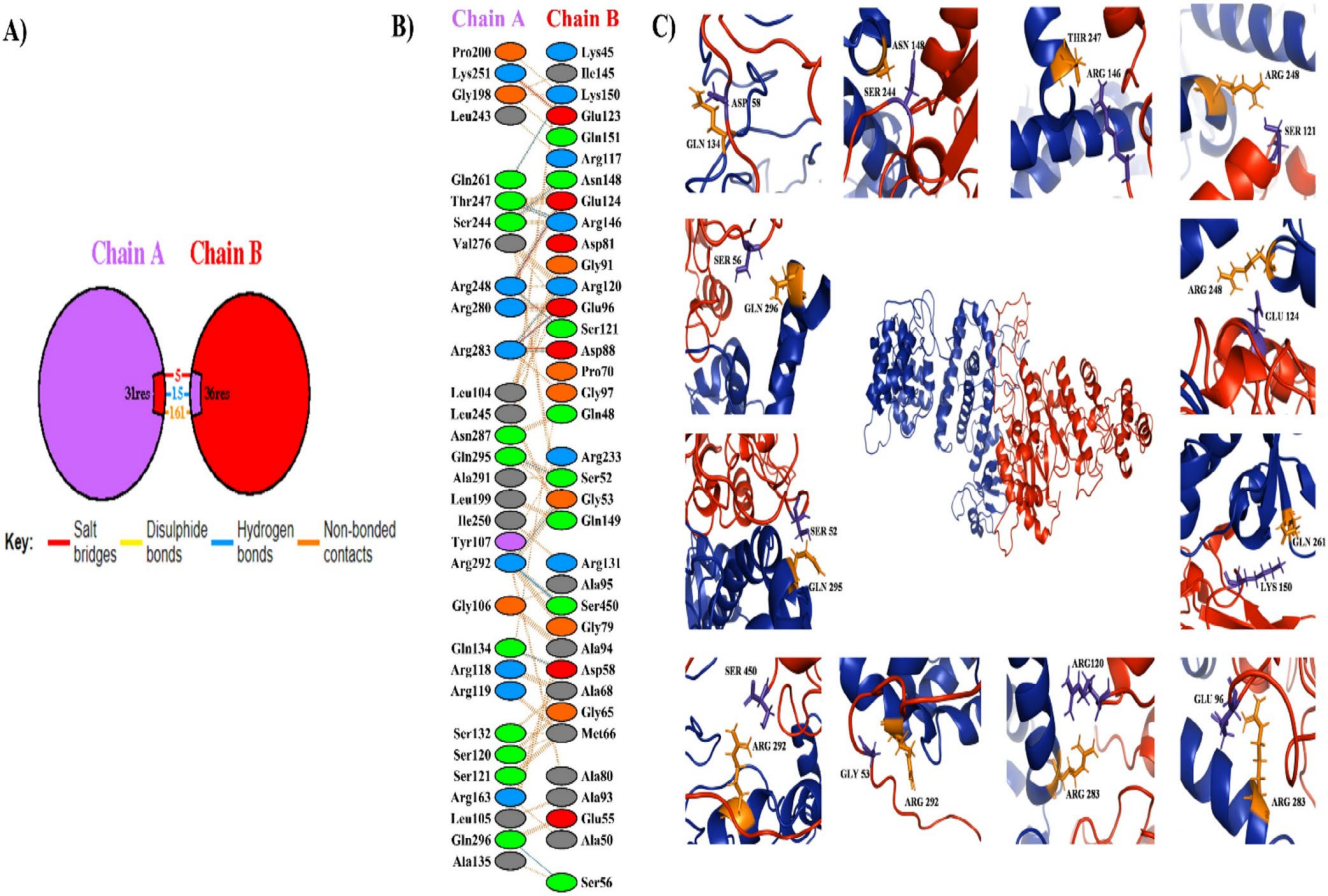
(A, B) 2D interaction between *LdPriL* (Chain A) and *LdPriS* (Chain B) by PDBsum showing the formation of 15 H‐bonds. Hydrogen bonds are shown with blue color lines, salt bridges with red color lines, and other contacts with orange color lines, (C) The interaction pattern between *LdPriL* (blue and cartoon) and *LdPriS* (red and cartoon) in *LdPri* complex. The interacting residues of *LdPriL* are shown in orange and sticks while interacting residues of *LdPriS* are shown in violetpurple and sticks.

Interaction analysis between *LdPriL* and *LdPriS* in the *LdPri* complex by PDBsum evaluated 15 hydrogen bonds (GLN134‐ASP58, SER244‐ASN148, THR247‐ARG146, THR247‐ARG146, ARG248‐SER121, ARG248‐GLU124, GLN261‐LYS150, ARG283‐GLU96, ARG283‐ARG120, ARG292‐GLY53, ARG292‐SER450, ARG292‐SER450, GLN295‐SER52, GLN296‐SER56) and five salt bridges (ARG248‐GLU124, LYS251‐GLU123, ARG280‐GLU96, ARG283‐ASP88, ARG283‐GLU96) formed between *LdPriL* and *LdPriS*, respectively (Figure 1A,B). The H‐bond interaction pattern between *LdPriL* and *LdPriS* residues in *the LdPri* complex is shown in Figure 1C.

The Gibbs free energy (ΔG) of the *LdPri* complex after the protein–protein docking was −15.7 kcal/mol, indicating the stability of the docked complex thermodynamically (Ghosh et al. 2021). The dissociation constant (*Kd*) of 8.0E‐12 M of the docked complex was evaluated by PRODIGY.

### 3.4 Alanine Fingerprinting and Evaluation of Hot Spot Residues

PPCheck server for studying the change in the binding free energy for the mutational study of the active site residues of both *LdPriL* and *LdPriS* identified *LdPriL* protein with significant free energy differences for the considered active site residues structure, as well as energy per residue except residues TYR412, HIS416 and SER426 in *LdPriL*, which were associated with no or negative energy difference after in silico alanine substitution and hence protein stability is not altered after mutation (Figure S4A). While *LdPriS* was identified with significant energy differences for all the considered active site residues after alanine substitution, residues ASP166, ARG220, and ASP168 possessed the most significant free energy difference of 32.6, 30.48, and 26.25 kcal/mol, respectively (Figure S4B). Thus, in silico alanine‐scanning mutation analysis by PPCheck suggested instability at individual residues of *LdPriL* and *LdPriS* within the *LdPri* complex (Sharma et al. 2022). The details of the binding free energy difference for the residues of both *LdPriL* and *LdPriS* by PPCheck are given in Tables S5 and S6, respectively. The *LdPri* protein complex and the evaluated hotspot sites of both the subunits for molecular docking studies are depicted in Figure S5.

### 3.5 Molecular Docking and Interaction Analysis

Molecular docking of natural anti‐leishmanial, DNA/RNA synthesis, and PPI inhibitors against individual *LdPriL* and *LdPriS* proteins and the *LdPri* complex provided descriptive insights into molecular interactions. Epigallocatechin Gallate (EGCG) and p‐Coumaric acid (4‐Hydroxycinnamic acid), among the natural anti‐leishmanial and DNA/RNA synthesis inhibitors, as well as PPI inhibitor Pritelivir (BAY 57‐1293), were found to be the most efficient toward individual *LdPriL* and LdPriS proteins as well as the *LdPri* complex against the hotspot residues and possessing the least binding energy (Tables S7–S11).

2D Interactions analysis between the EGCG, p‐Coumaric acid, and Pritelivir against individual *LdPriL* and *LdPriS*, as well as against *LdPriL* and *LdPriS* in *LdPri* complex, and against the interacting residues between *LdPriL* and *LdPriS* in *LdPri* complex are depicted in Table S10. LigPlot analyses showed H‐bond interaction of EGCG with hotspot residue HIS 363 in individual *LdPriL* and TYR410 of *LdPriL* in *the LdPri* complex (Figure S6). EGCG, on the other hand, formed an H‐bond with the hotspot residues ASP166, ASP168, and ARG220 in individual *LdPriS* and ASP166 of *LdPriS* in *the LdPri* complex (Figure S7). p‐Coumaric acid formed an H‐bond with hotspot residue HIS366 in *LdPriL* protein and LYS365 of *LdPriL* in *the LdPri* complex (Figure S8). Further, p‐Coumaric acid formed an H‐bond with hotspot residue ASP166 and ASP171 in *LdPriS* protein and ASP171 of *LdPriS* in the *LdPri* complex (Figure S9). Pritelivir interacted with residues THR408 and THR427 in *LdPriL* and HIS366 of *LdPriL* in *the LdPri* complex (Figure S10). Pritelivir also formed an H‐bond with hotspot residues ASP168 in *LdPriS* and ASP166 of *LdPriS* in *the LdPri* complex (Figure S11).

**FIGURE 2:**
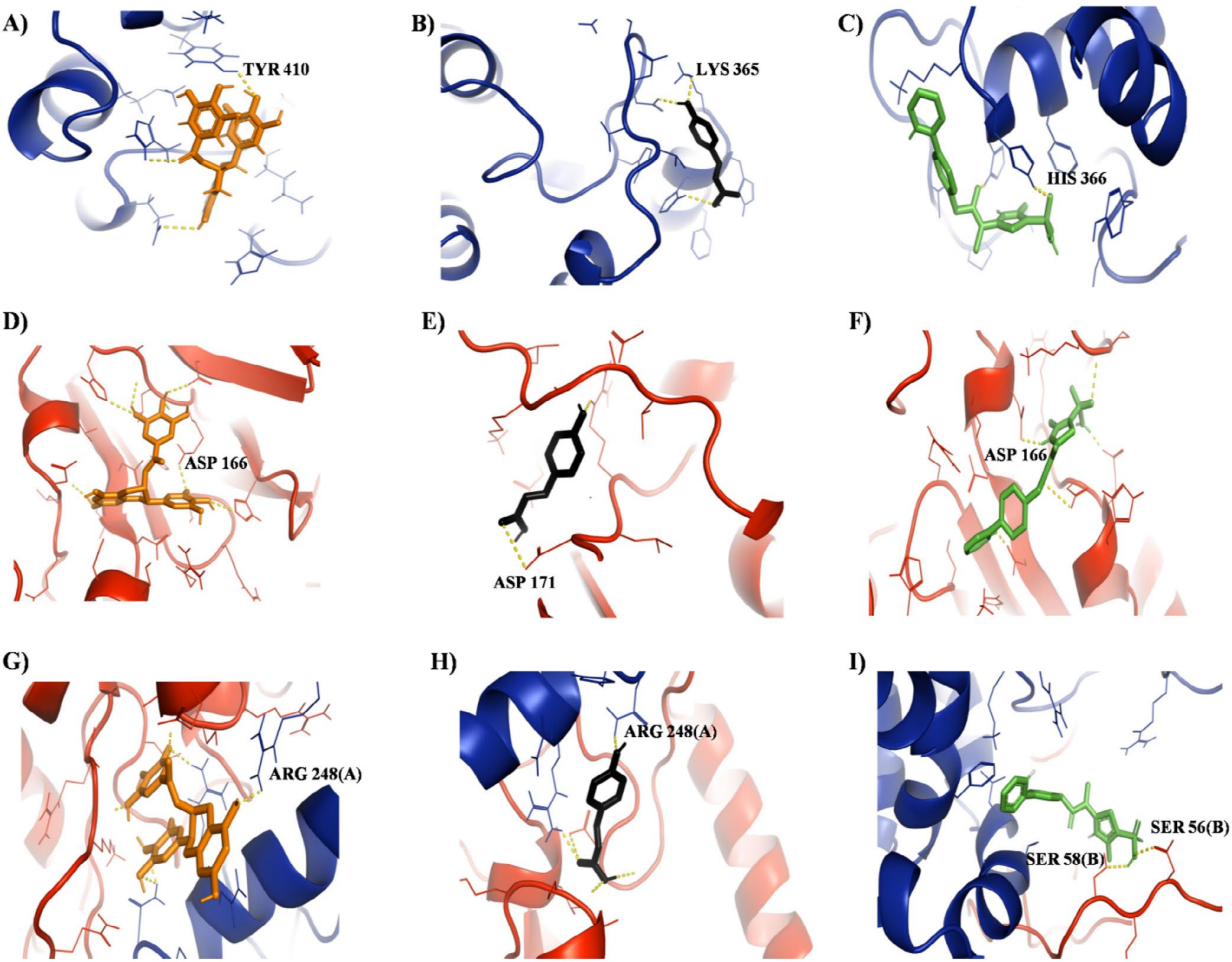
3D representation of the key H‐bond interaction of (A) EGCG (orange and sticks) with *LdPriL* (blue and cartoon) in *LdPri* complex, (B) p‐Coumaric acid (black and sticks) with *LdPriL* (blue and cartoon) in *LdPri* complex, (C) Pritelivir (green and sticks) with *LdPriL* (blue and cartoon) in *LdPri* complex, (D) EGCG (orange and sticks) with *LdPriS* (red and cartoon) in *LdPri* complex, (E) p‐Coumaric acid (black and sticks) with *LdPriS* (red and cartoon) in *LdPri* complex, (F) Pritelivir (green and sticks) with *LdPriS* (red and cartoon) in *LdPri* complex, (G) EGCG (orange and sticks) with *LdPriL* (blue and cartoon) and *LdPriS* (red and cartoon) interaction residues in *LdPri* complex, (H) p‐Coumaric acid (black and sticks) with *LdPriL* (blue and cartoon) and *LdPriS* (red and cartoon) interaction residues in *LdPri* complex, (I) Pritelivir (green and sticks) with *LdPriL* (blue and cartoon) and *LdPriS* (red and cartoon) interaction residues in *LdPri* complex.

H‐bond interactions study in LigPlot between EGCG, p‐Coumaric acid, and Pritelivir with the interacting residue between *LdPriL* and *LdPriS* in *LdPri* complex also revealed the interaction of these selected ligands with the core residues in *LdPriL* and *LdPriS* interaction to form *LdPri* complex, and further may act as potential PPI inhibitors (Figure S12). The 3D interactions between EGCG, p‐Coumaric acid, and Pritelivir against the different hotspot residues in the *LdPri* complex using Pymol (Lill and Danielson 2011) are provided in Figure 2.

The binding energy of EGCG, p‐Coumaric acid, and Pritelivir against both the large and small subunits of Human DNA Primase active site residues (Table S12) by PyRx was found to be poor (Table S13). Also, the interaction analysis with PyMOL for all three ligands against both subunits of Human DNA primase showed no interactions with active‐site residues considered in this study (Table S14, Figure S13).

### 3.6 MD Simulation

**FIGURE 3:**
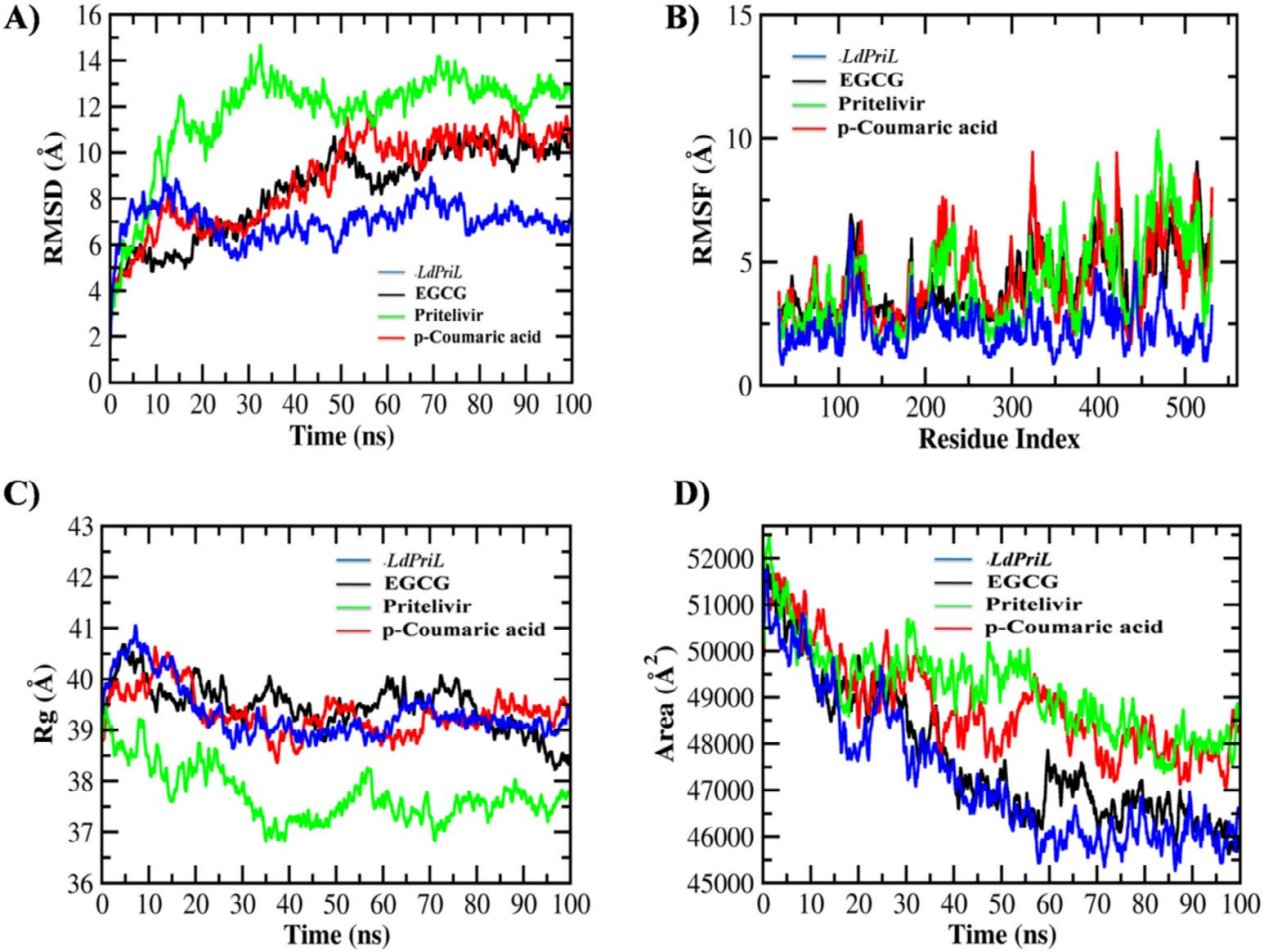
Analysis of molecular dynamics simulation results of EGCG (black), p‐Coumaric (red) acid and Pritelivir (green) and *LdPriL* apo (blue) for **Set 1** (A) Root mean square deviation (RMSD), (B) root mean square fluctuation (RMSF), (C) radius of gyration (Rg), (D) solvent accessible surface area (SASA).

**FIGURE 4:**
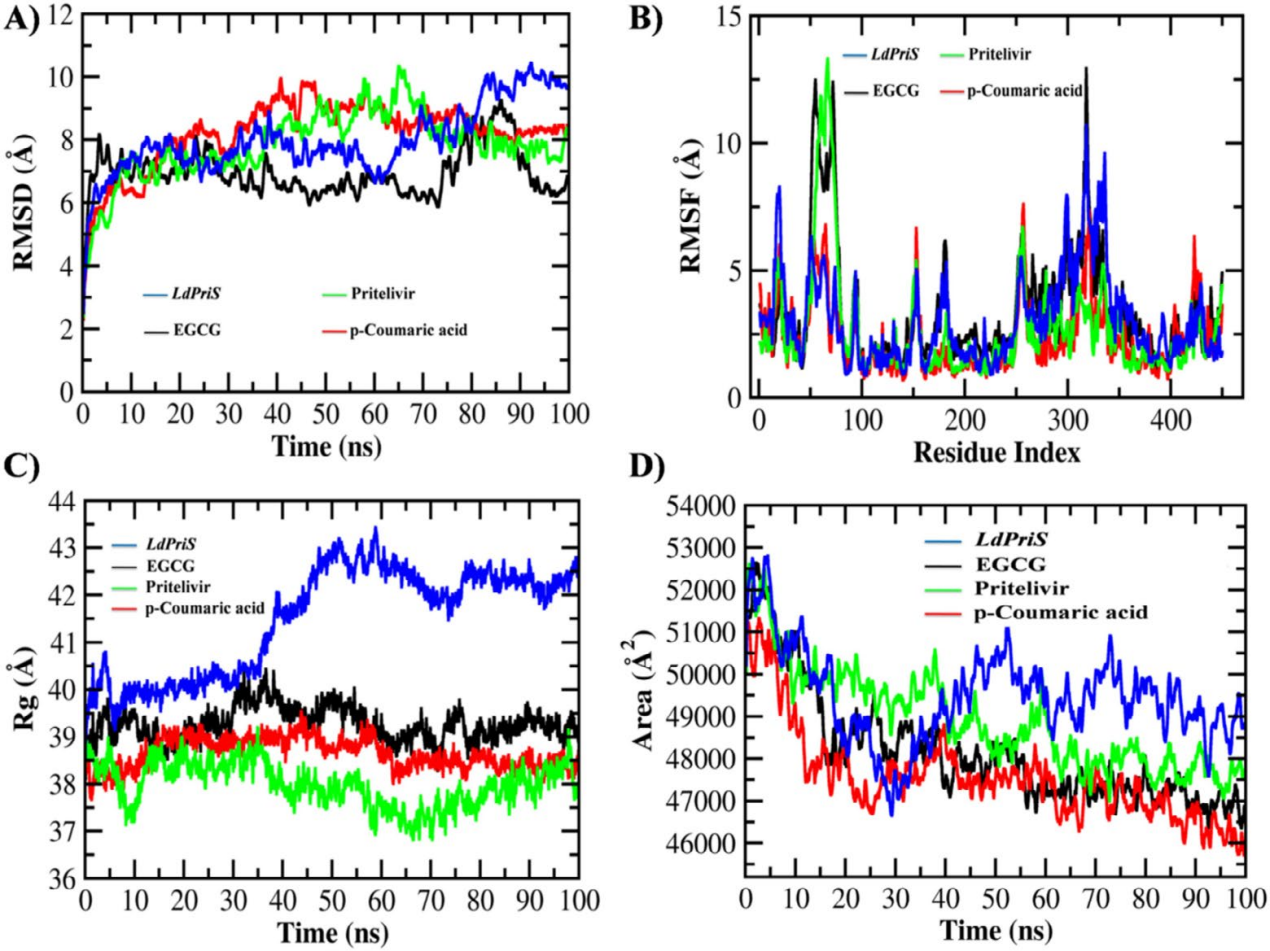
Analysis of molecular dynamics simulation results of EGCG (black), p‐Coumaric (red) acid and Pritelivir (green) and *LdPriS* apo (blue) for **Set 2** (A) Root mean square deviation (RMSD), (B) root mean square fluctuation (RMSF), (C) Radius of gyration (Rg), (D) solvent accessible surface area (SASA).

**FIGURE 5:**
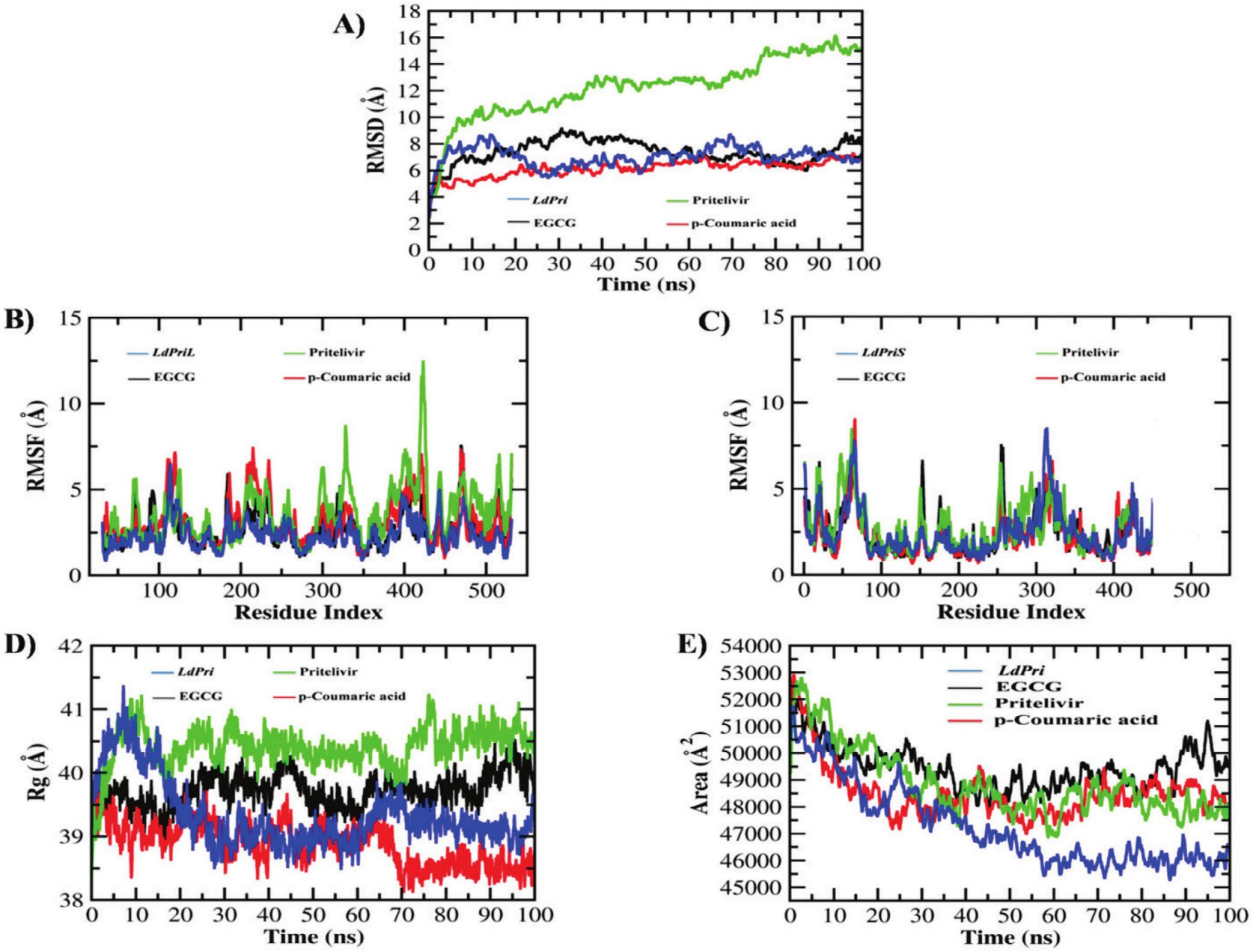
Analysis of Molecular Dynamics Simulation results of EGCG (black), p‐Coumaric (red) acid, Pritelivir (green) and *LdPri* complex (blue) for **Set 3** (A) root mean square deviation (RMSD), (B, C) root mean square fluctuation (RMSF), (D) radius of gyration (Rg), (E) solvent accessible surface area (SASA).

**FIGURE 6:**
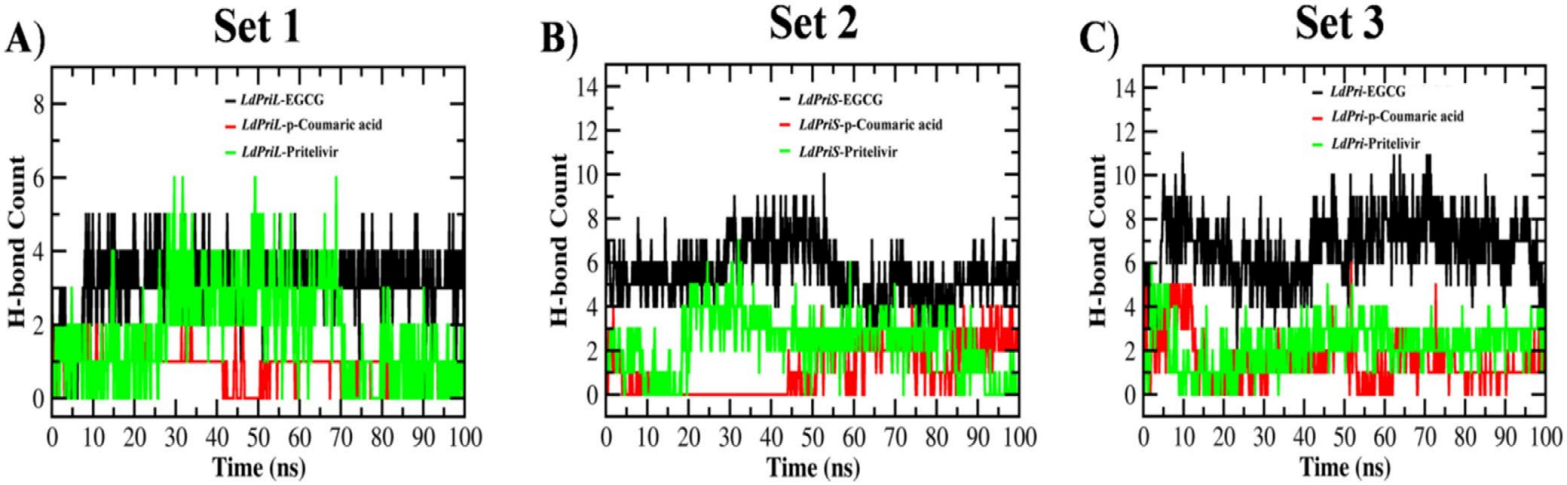
Analysis of molecular dynamics simulation results of intermolecular H‐bonds for EGCG (black), p‐Coumaric (red) acid and Pritelivir (green) in **Set (1–3)**.

Through 100 ns MD simulation parameters, including RMSD, RMSF, the Radius of gyration (Rg), SASA energy, and hydrogen bonds, were used to determine the structural stability and consistency of the docked complexes, that is, EGCG, p‐Coumaric acid, and Pritelivir with *LdPriL* apo (**Set 1**), *LdPriS* apo (**Set 2**), and *LdPri* complex (**Set 3**) (Figures 3, 4, 5, 6).

#### 3.6.1 Root Mean Square Deviations (RMSD)

The RMSD study clarifies the structural variations of whole protein molecules over time (Amera et al. 2020). From the time‐averaged RMSD of the unbound *LdPri* complex and Set 1, that is, EGCG, p‐Coumaric acid, and Pritelivir with *LdPriL* apo, the fluctuation was observed more in the inhibitor‐bound *LdPri* than in the unbound form. Though fluctuations were observed in both unbound *LdPriL* in *the LdPri* complex and **Set 1** during the initial 35 ns, stability was observed with minimal fluctuations throughout the (35–100) ns, with RMSD values of 0.2 for EGCG and 0.3 for p‐Coumaric acid. Pritelivir‐bound *LdPriL* in *LdPri* complex in **Set 1** were found to be < 1.5 nm (15 Å) (Figure 3A).

While stability in Set 2, that is, EGCG, p‐Coumaric acid, and Pritelivir with *LdPriS* apo for the considered inhibitors was observed for more than 65 ns, EGCG‐bound *LdPriS* in the *LdPri* complex showed the lowest fluctuations, < 0.9 nm (9 Å), than the unbound form. Pritelivir showed the highest fluctuation of ≤ 0.1 nm (10 Å). High fluctuations were noted for final (70–100) ns for unbound *LdPriS* in *LdPri* complex than **Set 2**, with Pritelivir stabilized highly than EGCG with fluctuation of ≤ 0.85 nm (8.5 Å) (Figure 4A). EGCG, followed by Pritelivir, was considered a potent binder of the catalytic primase subunit (*LdPriS*) in *the LdPri* complex, with superior Cα backbone stability.

Lastly, EGCG and p‐Coumaric acid were found to form the most stable structure in the interaction site between *LdPriL* and *LdPriS* in *LdPri* complex (**Set 3**), with fluctuation ranging from (0.4–0.9) nm, that is, (4–9 Å) in the final complex. In contrast, the increased fluctuation was associated with the Pritelivir‐bound complex (Figure 5A), indicating that p‐Coumaric acid and EGCG are potential PPI inhibitors and may act in a non‐competitive or uncompetitive manner, unlike Pritelivir, which was more competitive toward the active‐site residues evaluated for *LdPri* catalytic activity.

#### 3.6.2 Root Mean Square Fluctuations (RMSF)

Additionally, the Root mean square fluctuation (RMSF) of the unbound *LdPri* and for the Cα atom of each amino acid residue for Set (1–3) was established over the whole simulation to evaluate the elasticity of the residues in Set (1–3) (John et al. 2017). High RMSF fluctuations reveal more flexibility, while low RMSF fluctuations during MD simulation reveal restrictions on residue mobility and more stability. RMSF fluctuations for **Set 1**, that is, EGCG, p‐Coumaric acid, and Pritelivir with *LdPriL* apo in the predicted active site regions of *LdPriL* (363–427) in *the LdPri* complex were found to be < 1 nm (10 Å) with arbitrary fluctuations throughout the whole complex, particularly toward the N‐terminal domain (Figure 3B).

Furthermore, it was found that the variations in the *LdPriS* active‐site residues were minor in the *LdPriS* apo complex upon binding with the selected inhibitors (**Set 2**), that is, EGCG, p‐Coumaric acid, and Pritelivir. Pritelivir and p‐Coumaric acid showed the slightest fluctuations < 0.3 nm (3 Å), even better than the unbound form, indicating an increase in the stability due to hydrogen bond formation with the inhibitors, indicating the inhibitors to be more efficient against *LdPriS* than *LdPriL* in inhibiting *LdPri* activity (Figure 4B).

Finally, analysis of the RMSF fluctuations for **Set 3**, that is, EGCG, p‐Coumaric acid, and Pritelivir with *LdPri* complex, indicated a low RMSF (< 0.3 nm, i.e., 3 Å) for EGCG and p‐Coumaric acid against *LdPriL* (ARG 248) as well as for Pritelivir against *LdPriS* (SER 56 and ASP 58) in the interaction site between *LdPriL* and *LdPriS* in *LdPri* complex, respectively (Figure 5B,C). Fluctuations for **Set 3** for both *LdPriL* and *LdPriS* amino acid residues in the *LdPri* complex remained impressively constant.

#### 3.6.3 Radius of Gyration (*R*_g_)

The radius of gyration (Rg) analysis for **Set (1–3)** was also used to determine the homogeneity or unfolding of the backbone atoms throughout the 100 ns MD simulation. The lowest Rg value was obtained for Pritelivir in **Set 1**, that is, EGCG, p‐Coumaric acid, and Pritelivir with *LdPriL* apo (Figure 3C), but the Rg rate remains the same in the other complexes. At the same time, all the inhibitors had lower Rg than the unbound form in **Set 2**, that is, EGCG, p‐Coumaric acid, and Pritelivir with *LdPriS* apo, indicating tight binding to the catalytic *LdPriS* and greater stiffness (Figure 4C).

Finally, only p‐Coumaric acid possessed the lowest Rg of 3.83 nm (38.3 Å) than the unbound *LdPri* complex for **Set 3**, that is, EGCG, p‐Coumaric acid, and Pritelivir with *LdPri* complex (Figure 5D), which indicated potential PPI inhibition of p‐Coumaric acid between *LdPriL* and *LdPriS* in *LdPri* complex.

#### 3.6.4 Solvent Accessible Surface Area (SASA)

Because solvents behave differently under varying conditions, solvent‐accessible surface area (SASA) is a valuable metric for understanding a protein's structural motion in aqueous environments. It was noticeable that binding of EGCG, p‐Coumaric acid, and Pritelivir against different hotspot site residues of *LdPri* complex in **Set (1–3)** was related to decreasing SASA throughout the 100 ns simulation (Figures 3D, 4D, and 5E). **Set 2**, that is, EGCG, p‐Coumaric acid, and Pritelivir with *LdPriS* apo showed decreased SASA for EGCG, p‐Coumaric acid, and Pritelivir than the unbound complex (Figure 4D). Thus, the decrease in SASA in all situations shows that SASA has reached a state of equilibrium during the simulation, indicating the structural stability of EGCG, p‐Coumaric acid, and Pritelivir against different hotspot site residues of *LdPri* complex in **Set (1–3)** (Figures 3D, 4D, and 5E).

#### 3.6.5 H‐Bond Count

Intermolecular H‐bonds were estimated using g_hbond to assess Sets **(1–3)**'s stability (Yang et al. 2017). An essential indicator of binding specificity is the presence of persistent hydrogen bonds between a receptor and an inhibitor molecule. H‐bond counts evaluated the strong interactions of EGCG with the hotspot site residues of the *LdPri* complex in Sets **(1–3)**, followed by Pritelivir (Figure 6A–C). Moreover, p‐Coumaric acid tended to disassemble from the complex in **Set 2** (Figure 6B) without any H‐bond formation during (15–45) ns MD simulation run, while maintaining at least a single H‐bond in **Set 1** and **3**, that is, EGCG, p‐Coumaric acid, and Pritelivir with *LdPriL* apo and, that is, EGCG, p‐Coumaric acid, and Pritelivir with *LdPri* complex (Figure 6A,C).

Comparative examination of various positions of 100 ns simulation of EGCG, p‐Coumaric acid, and Pritelivir against distinct *LdPri* complex hotspot site residues in Sets (1–3) was also shown (Figures S14–S16). Final posture plots in two dimensions (2D) were created and compared to the starting structure. In Sets **(1–3)**, the interaction of EGCG and Pritelivir inside the *LdPri* complex was stable and remained in the same active site pocket while p‐Coumaric acid leaves the pocket in **Set 2**, that is, EGCG, p‐Coumaric acid, and Pritelivir with *LdPriS* apo without forming any H‐bond during a 25 ns run, as evaluated during H‐bond count above and remained around the interacting site rather than the *LdPriS* catalytic site during (50–100) ns run (Figure S15).

#### 3.6.6 Principal Component Analysis

To better understand the conformational alterations, the effects of EGCG, p‐Coumaric acid, and Pritelivir on the hotspot sites of the *LdPri* complex in Sets **(1–3)** and on the conformational mobility of the entire LdPri complex were examined using PCA in the current work. A 2D projection of each of the two main principal components (PC1 and PC2) was created using ED, separating the structural subspace into critical subspaces (Verma et al. 2017) for EGCG, p‐Coumaric acid, and Pritelivir against *LdPri* complex in Sets **(1–3)**. As shown in Figure S17, the eigenvectors calculated from the MD motion for every model differ significantly, illustrating the diversity in molecular dynamics within the systems and perhaps leading to structural shifts within the **Sets (1–3)**. As a result, in PCA analysis, the less colonized the conformation space is, the stronger the inhibiting potency of the confined ligand toward the protein structure (Zarezade et al. 2018). Our findings for Sets **(1–3)** after PCA showed that Pritelivir had a substantial inhibitory impact in **Sets 1**, that is, EGCG, p‐Coumaric acid, and Pritelivir with *LdPriL* apo, and **2**, that is, EGCG, p‐Coumaric acid, and Pritelivir with *LdPriS* apo (Figure S17C,F), with less explored structural space, indicating that combined movements manifest to facilitate protein‐inhibitor interaction. Whereas a smaller explored conformational space was seen for EGCG and p‐Coumaric acid (Figure S17G,H) than Pritelivir (Figure S17I) in Set 3, that is, EGCG, p‐Coumaric acid, and Pritelivir with *LdPri* complex indicated Pritelivir to be an inhibitor of *LdPri* precisely and efficiently utilizing the active site regions for both *LdPriL* and *LdPriS*, and might interfere competitively for substrate utilization. At the same time, EGCG and p‐Coumaric acid may act as potent PPI inhibitors, either non‐competitively or uncompetitively.

#### 3.6.7 Binding Free Energy Calculation

The binding free energies of the protein‐ligand complexes in sets (1–3) were determined using GROMACS's g_mmpbsa tool, with the final 20 ns (80–100 ns) of MD simulation data used for analysis. Calculation of binding free energy (*ΔEbinding*), van der Waals energy (*ΔEvdw*), electrostatic energy (*ΔEelec*), polar solvation energy (*ΔEpolar*), and SASA for each receptor‐inhibitor complex in **Sets (1–3)** was carried out (Table S11). Only p‐Coumaric acid had a positive binding free energy in **Set 2**, that is, EGCG, p‐Coumaric acid, and Pritelivir with *LdPriS* apo, indicating that p‐coumaric acid binding constituted a random kinetic procedure. The binding free energy indicates that Pritelivir interacts more efficiently with the active site than with the interaction site of both subunits in *the LdPri* complex, and may inhibit the catalytic activity of *LdPri*. EGCG and p‐Coumaric acid were efficient at inhibiting the interaction site between *LdPriL* and *LdPriS* in the *LdPri* complex, as evaluated during MD simulations. The calculated van der Waal energy was lowest for Pritelivir in **Set 1**, that is, EGCG, p‐Coumaric acid, and Pritelivir with *LdPriL* apo. In contrast, the lowest van der Waals energy for EGCG and p‐Coumaric acid was seen for **Set 3**, that is, EGCG, p‐Coumaric acid, and Pritelivir with *LdPri* complex; electrostatic energies, on the other hand, for EGCG, p‐Coumaric acid, and Pritelivir were lowest for **Set 3**. The overall binding free energy across all **Sets** was positively affected by the polar solvation energy of the inhibitors Pritelivir and EGCG. SASA energy for EGCG, p‐Coumaric acid, and Pritelivir was lowest for **Set 3**. Finally, our study indicated that the binding of EGCG and Pritelivir to the *LdPri* complex in **Sets (1–3)** was genuine and kinetically viable. However, the binding of p‐Coumaric acid in **Set 2** was random and unsustainable. The comparison of inhibitor energies (EGCG, p‐Coumaric acid, and Pritelivir) binding to the different hotspot sites in the *LdPri* complex for **Sets (1–3)** is shown in Figure S18.

### 3.7 Overexpression and Purification of *LdPriL* and *LdPriS*

The His tag constructs LdPriL‐pASK‐IBA43plus and LdPriS‐pET‐28a(+) expressed the recombinant proteins *LdPriL* and *LdPriS*, with molecular weights of 62 and 50 kDa, respectively. The recombinant proteins were soluble and purified by affinity chromatography (Figure S19A,B). The purified products were further desalted using Pierce Protein Concentrators with a 10 MWCO (PES, Thermo Scientific). The concentration of both the purified *LdPriL* and *LdPriS* by Bradford assay (Kruger 1994) was found to be 38.6 mg/mL (0.62 mM) and 38.2 mg/mL (0.77 mM), respectively (*R* ^2^ = 0.99) (Figure S19C). Western blot analysis with a 6× His‐tagged rabbit Monoclonal antibody (NovagenR) detected up to 1 ng of each recombinant protein. In contrast, the titer of the 6× His‐tagged Monoclonal antibody decreased with increasing dilution, suggesting the purity of our recombinant *LdPriL* and *LdPriS* and the specificity of the selected Ab (Figure S20A–C). The absorbance ratio A260/A280 was used to assess the purity of the isolated *LdPriL* and *LdPriS*. For specific proteins, an A260/A280 ratio of 0.6 is appropriate. Higher ratios can indicate DNA contamination of isolated proteins (Wilfinger et al. 1997). *LdPriL* and *LdPriS* purified by metal‐affinity chromatography showed A260/A280 ratios of 0.61 and 0.63, respectively, and yielded > 95% purified products. Detailed experimental protocols and buffer compositions have been provided in the Supporting Information.

### 3.8 Optimization of Primase‐Pyrophosphatase Assay Conditions and Biochemical Characterization of *LdPri*

The DNA primase activity of *LdPri* was assessed using a non‐radioactive primase‐pyrophosphatase assay. Biochemical characterization was performed by optimizing various parameters in the primase‐pyrophosphatase assay for *LdPri*. The in vitro condition was maintained to enhance protein‐DNA interaction. The reaction mixture was added with the significant electrolyte, potassium glutamate (KGlu) (Leirmo et al. 1987). The biochemical property of *LdPri* is to synthesize a primer on the M13ssDNA template; to test these parameters, various reaction conditions were tested.

**FIGURE 7:**
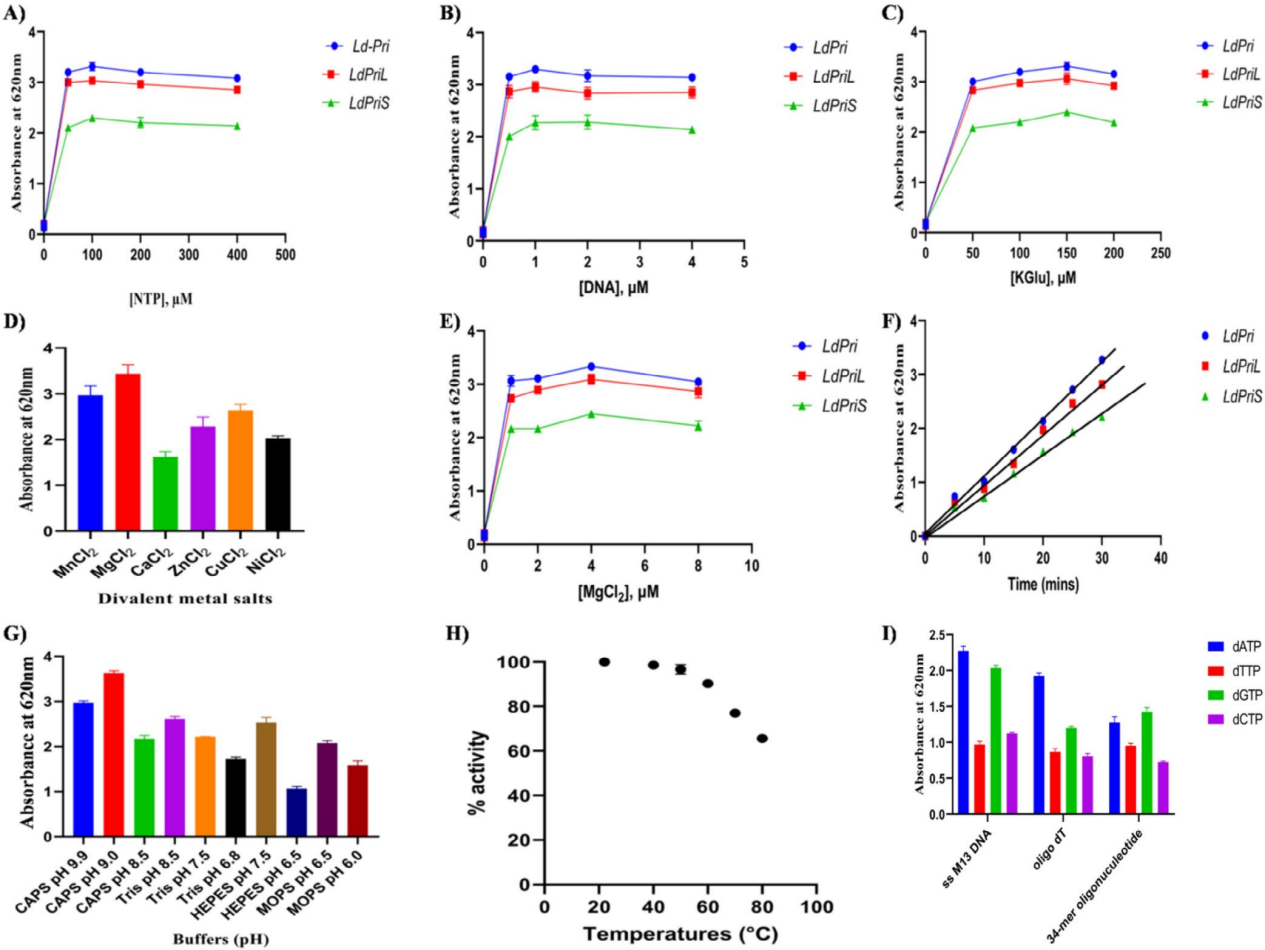
Optimization of *LdPri* activity by primase‐pyrophosphatase assay under different conditions and parameters, (A) Optimization of *LdPriL*, *LdPriS* and *LdPri* Activity as a function of the concentration of NTP, (B) Optimization of *LdPriL*, *LdPriS* and *LdPri* Activity as a function of the concentration of DNA, (C) Optimization of *LdPriL*, *LdPriS* and *LdPri* Activity as a function of the concentration of potassium glutamate, (D) Optimization of *LdPri* Activity in the presence of different monovalent and divalent metals at 2 mM, (E) Optimization of *LdPriL*, *LdPriS* and *LdPri* Activity as a function of the concentration of MgCl_2_, (F) Linear time course of the primase activity of *LdPriL*, *LdPriS* and *LdPri* by colorimetric primase‐pyrophosphatase assay, (G) Optimization of *LdPri* Activity in a variety of buffers (20 mM) at different pH range, (H) *Thermostability* of *LdPri* Activity. Activity was checked upto 80°C and (I) The 3′‐terminal nucleotidyl‐transferase activity of *LdPri* on different DNA templates.

The primase activity assay was optimized under various conditions and parameters to maximize activity (Figure S21). *LdPri*, along with individual studies on *LdPriL* and *LdPriS*, was conducted to measure primase activity as a function of dNTP concentration, ss M13 DNA, the divalent metal of choice, and potassium glutamate (KGlu) (Figure 7A–E). *LdPri*, *LdPriL*, and *LdPriS* all displayed a similar concentration requirement of the measured parameters in the reaction mixture for their maximal activity. *LdPri* activity was checked for different divalent metals (Mn^2+^, Mg^2+^, Ca^2+^, Zn^2+^, Cu^2+^, Ni^2+^), and it was seen that the *LdPri* activity was higher in the presence of the divalent metal Mg^2+^ at an optimum concentration of 4 mM (Figure 7D,E). The *LdPri* activity was optimal in the physiological KGlu concentration range of 110–150 mM (Figure 7C). The absorbance versus time graph showed linear primase activity for *LdPriL*, *LdPriS*, and the *LdPri* complex (Figure 7F). The most noticeable difference was that *LdPriL* primase activity was higher, almost twice that of *LdPriS* alone. Primase activity of *LdPriS* alone was detectable, suggesting that *the LdPriS* subunit is very labile, unstable, and inefficient in the absence of a large subunit (Copeland and Tan 1995; Schneider et al. 1998; Kuchta and Stengel 2010).

1. Effect of pH: *LdPri* activity was observed at different pHs (pH = 6–9.9), preferring a higher pH for optimal activity. Efficient *LdPri* activity was observed in 20 mM CAPS at pH 9.0 among the tested buffers and pH values (Figure 7G).
2. Thermostability test: Following a 15‐min pre‐incubation at room temperature, the primase‐pyrophosphatase test was used to assess the heat sensitivity of *LdPri* activity. *LdPri* activity was resistant up to 60°C, with a 10% loss relative to the initial activity. Further, with increasing temperature, *LdPri* activity declined by 25% and 35% at 70°C and 80°C, respectively (Figure 7H).
3. *LdPri* has 3′‐terminal nucleotidyltransferase activity: The 3′‐terminal transferase activity of *LdPri* was tested with the different selected single‐stranded oligonucleotides of varied lengths and thought to be extended by *LdPri* in the presence of dNTPs. All four tested dNTPs (dATP, dCTP, dGTP, dTTP) were incorporated individually and at different potentials due to variations in DNA template lengths (Figure 7I). Thus, we could hypothesize that primer synthesis by *LdPri* was template‐directed and independent of the template sequence.

### 3.9 Primase Inhibition Assay

**FIGURE 8:**
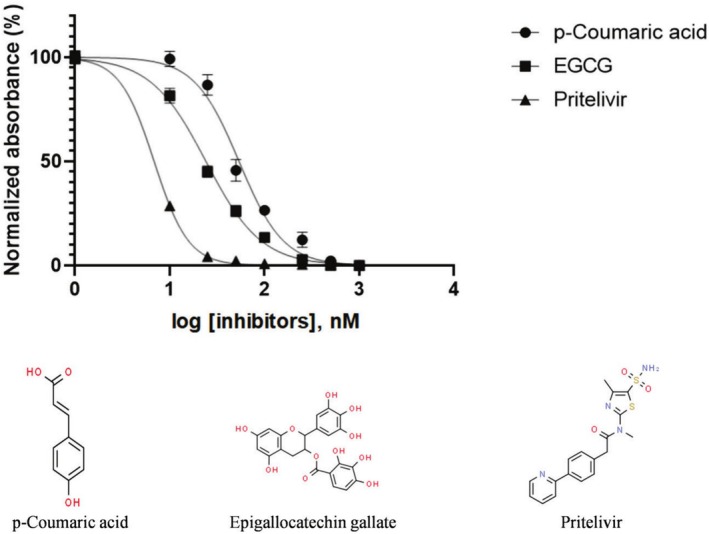
Dose–response plot for Pritelivir, EGCG (fourfold higher), and p‐Coumaric acid (eightfold higher) from primase inhibition studies in 96‐well clear polystyrene plates (upper panel) along with the 2D diagrammatic pictures (lower panel) of the best inhibitors against *Leishmania donovani* nuclear DNA primase (*LdPri*) complex upon molecular docking. All the experiments were done in triplicates.

Using non‐linear regression equations, the inhibitory activities of EGCG, p‐Coumaric acid, and Pritelivir on *LdPri* activity were expressed as IC_50_ values. It is shown in the graph (Figure 8). The regression data yielded IC_50_ values of 24.21 ± 3.6 nM (*R* ^2^ = 0.99), 53.77 ± 3.24 nM (*R* ^2^ = 0.98), and 6.85 ± 0.09 nM (*R* ^2^ = 0.99) for EGCG (*p* = 0.0481), p‐Coumaric acid (*p* = 0.0991), and Pritelivir (*p* = 0.0095), respectively, indicating their inhibitory effects against *LdPri* activity. EGCG displayed approximately fourfold, and p‐Coumaric showed eightfold weaker inhibition than Pritelivir. The dose–response curves of EGCG, p‐Coumaric acid, and Pritelivir from the non‐linear regression equations generated Hill coefficients of 1.45 ± 0.21, 1.7 ± 0.77, and 2.45 ± 0.45, respectively, indicating multiple inhibitor‐binding sites in *LdPri* (Prinz 2010). Moreover, while studying the inhibitory activity of EGCG, p‐Coumaric acid, and Pritelivir on individual *LdPriL* and *LdPriS* activity, it was surprising to notice that primase activity could still be observed with increasing concentrations of p‐Coumaric acid on *LdPriL* (*p* = 0.3147) and *LdPriS* (*p* = 0.2887) and was not characterized further, while EGCG and Pritelivir were both inhibitory to the individual proteins.

### 3.10 Analysis of the Mode of Inhibition

#### 3.10.1 Competitive Inhibition by Pritelivir

**FIGURE 9:**
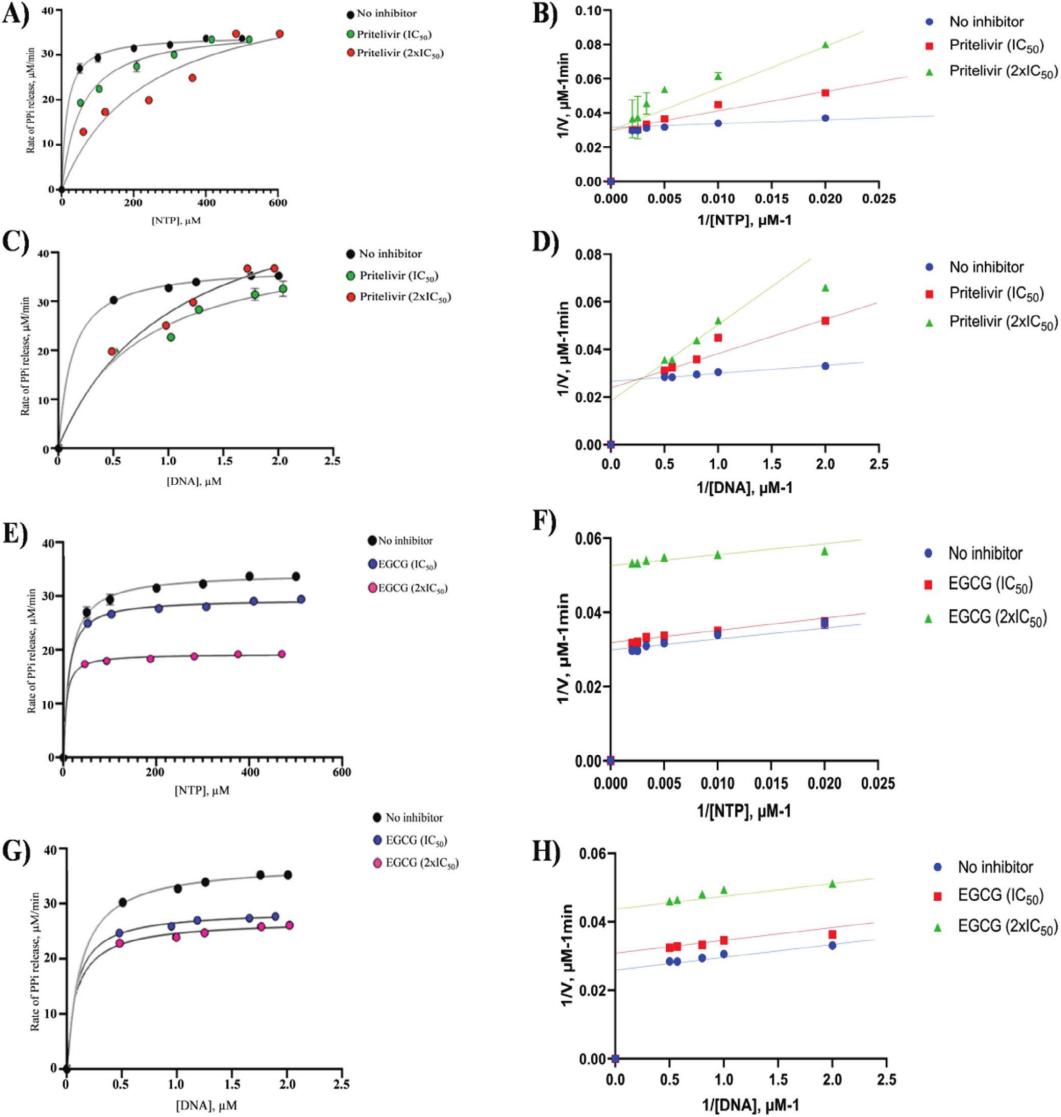
(A) The steady‐state rate of PPi release by *LdPri* as a function of NTP concentration (50, 100, 200, 300, 400, and 500 μM, respectively) in the absence and presence of inhibitor Pritelivir (IC_50_, 2× IC_50_), (B) Lineweaver–Burk plot for inhibition of *LdPri* in the presence of Pritelivir (IC50, 2× IC50) and substrate NTP at varying concentrations (50, 100, 200, 300, 400, and 500 μM, respectively), (C) the steady‐state rate of PPi release by *LdPri* as a function of DNA concentration (0.5, 1, 1.25, 1.75, and 2 μm, respectively) in the absence and presence of inhibitor Pritelivir (IC_50_, 2× IC_50_), (D) Lineweaver–Burk plot for inhibition of *LdPri* in the presence of Pritelivir (IC50, 2× IC50) and substrate DNA at varying concentrations (0.5, 1, 1.25, 1.75 and 2 μM, respectively), (E) The steady‐state rate of PPi release by *LdPri* as a function of NTP concentration (50, 100, 200, 300, 400, and 500 μM, respectively) in the absence and presence of inhibitor EGCG (IC_50_, 2× IC_50_), (F) Lineweaver–Burk plot for inhibition of *LdPri* in the presence of EGCG (IC50, 2× IC50) and substrate NTP at varying concentrations (50, 100, 200, 300, 400, and 500 μM, respectively), (G) The steady‐state rate of PPi release by *LdPri* as a function of DNA concentration (0.5, 1, 1.25, 1.75, and 2 μm, respectively) in the absence and presence of inhibitor EGCG (IC_50_, 2× IC_50_) and (H) Lineweaver–Burk plot for inhibition of *LdPri* in the presence of EGCG (IC50, 2× IC50) and substrate DNA at varying concentrations (0.5, 1, 1.25, 1.75, and 2 μM, respectively). All the experiments were done in triplicates. The bars indicate the standard deviation.

The mode of inhibition was analyzed using Lineweaver–Burk plots of enzyme activity (i.e., the change in absorbance over time, indicating velocity (1/*V*)) plotted against different substrate concentrations (1/[*S*]). The linear regression in the Lineweaver–Burk plot allows us to determine changes in the Michaelis constant (*K*_m_) and enzyme velocity (*V*_max_). Lineweaver–Burk plot for *LdPri* activity in the absence and presence of Pritelivir (IC_50_, 2× IC_50_) for both the substrates, that is, NTP and DNA displayed the same *V*_max_ (the slight change in *V*_max_ were neglected in comparison to a high difference in *K*_m_ due to minor experimental error) but that the *K*_m_ has increased threefold from 14.07 ± 3.22 μM (*R* ^2^ = 0.95) to 42.95 ± 2.61 μM (*R* ^2^ = 0.93) and sixfold upto 89.08 ± 2.09 μM (*R* ^2^ = 0.98) in the presence of the Pritelivir (IC_50_, 2× IC_50_) respectively for substrate NTP. In contrast, *K*_m_ increased fivefold from 0.1 ± 0.02 μM (*R* ^2^ = 0.95) to 0.54 ± 0.05 μM (*R* ^2^ = 0.89) and sevenfold up to 0.77 μM (*R* ^2^ = 0.97) in the presence of Pritelivir (IC_50_, 2× IC_50_), respectively, for substrate DNA. The steady‐state rate of PPi release by *LdPri* as a function of substrates NTP and DNA in the presence and absence of Pritelivir (IC_50_, 2× IC_50_) is shown in Figure 9A,C. The confidence intervals for *V*_max_ Pritelivir (IC_50_ and 2× IC_50_) for substrate NTP was found to be (34.22–37.72) μM/min (*R* ^2^ = 0.93) and (36.56–39.05) (*R* ^2^ = 0.92) μM/min, respectively while *K*_m_ Pritelivir (IC_50_ and 2× IC_50_) for substrate NTP was found to be (40.96–64.48) μM (*R* ^2^ = 0.93) and (110.90–190.10) μM (*R* ^2^ = 0.92), respectively.

While the confidence intervals for *V*_max_ Pritelivir (IC_50_ and 2× IC_50_) for substrate DNA were found to be (37.66– 50.58) μM/min (*R* ^2^ = 0.90) and (37.85–51.30) μM/min (*R* ^2^ = 0.94), respectively, while *K*_m_ Pritelivir (IC_50_ and 2× IC_50_) for substrate DNA was found to be (0.47–1.07) μM (*R* ^2^ = 0.90) and (0.77–1.53) μM (*R* ^2^ = 0.94), respectively.

Pritelivir's Lineweaver–Burk plots exhibit the same *V*_max_ but higher *K*_m_, indicating that it is a competitive inhibitor of *LdPri* for both substrates, NTP and DNA, at different substrate and inhibitor concentrations (Figure 9B,D). Competitive inhibition hinders reversible substrate binding by the inhibitor, directly competing with the substrate for the enzyme's substrate‐binding site (Dean 2002).

The following equation describes the interaction between LdPri and Pritelivir (Brandt et al. 1987).

(7) $$\begin{array}{l} E+S⇄\mathrm{ES}\to E+P\\ +I\\ ↓↑\\ \mathrm{EI} \end{array}$$

where *E* is the enzyme, *I* is the inhibitor concentration, and *S* is the substrate concentration. The following equation was used to calculate *K*_*i*_ for Pritelivir‐mediated competitive inhibition (Brandt et al. 1987).

(8) $$K_{i}=\frac{I_{50}}{\left(S/K_{m}\right)+1}.$$

*I*_50_ is the inhibitor concentration required for 50% inhibition of the enzymatic reaction (i.e., *I*_50_ = IC_50_), and *K*_m_ is the Michaelis–Menten constant. The *K*_*i*_ values for Pritelivir were 1.41 ± 1.01 nM and 2.21 ± 0.83 nM for the substrates NTP and DNA, respectively. *K*_*i*_ is smaller than IC_50_ for competitive inhibition as *S* increases, indicating that *K*_m_ decreases (Brandt et al. 1987).

$$K_{i}<I_{50}\left(S>>K_{m}\;\text{for}\;\text{competitive}\;\text{inhibitors}\right).$$

#### 3.10.2 Uncompetitive Inhibition by EGCG

The interaction of the inhibitor with the enzyme‐substrate (ES) complex, rather than with the free enzyme, underlies uncompetitive inhibition. The ES complex is suppressed in the presence of the inhibitor, and the *V*_max_ is lowered. Unrestricted ES complexes have a lower *K*_m_ when the inhibitor is present because they require less substrate for half‐maximal saturation. Consequently, the uninhibited slope parallels the inhibited slope in the Lineweaver–Burk plot (Dean 2002), and EGCG was identified as an uncompetitive inhibitor of *LdPri* for both substrates (Figure 9F,H). Both *V*_max_ and *K*_m_ declined in the presence of EGCG (IC_50_, 2× IC_50_) for the substrates NTP and DNA. *V*_max_ declined from 34.48 μM/min (*R* ^2^ = 0.92) to 31.25 μM/min (*R* ^2^ = 0.94) and from 37.98 ± 0.82 μM/min (*R* ^2^ = 0.90) to 31.26 ± 0.97 μM/min (*R* ^2^ = 0.9) for substrates NTP and DNA respectively, while *K*_m_ declined from 14.07 ± 3.22 μM (*R* ^2^ = 0.95) to 0.7 ± 0.12 μM (*R* ^2^ = 0.91) and from 0.1 ± 0.02 μM (*R* ^2^ = 0.95) to 0.07 ± 0.03 μM (*R* ^2^ = 0.93) for substrates NTP and DNA respectively in the presence of EGCG (IC_50_).

While *V*_max_ declined from 34.48 μM/min (*R* ^2^ = 0.95) to 18.86 μM/min (*R* ^2^ = 0.98) and from 37.98 ± 0.82 μM/min (*R* ^2^ = 0.91) to 22.38 ± 0.28 μM/min (*R* ^2^ = 0.93) for substrates NTP and DNA, respectively, *K*_m_ declined from 14.07 ± 3.22 μM (*R* ^2^ = 0.95) to 3.3 ± 0.05 μM (*R* ^2^ = 0.91) and from 0.1 ± 0.02 μM (*R* ^2^ = 0.95) to 0.06 ± 0.001 μM (*R* ^2^ = 0.90) for substrates NTP and DNA respectively in the presence of EGCG (2× IC_50_). The steady‐state rate of PPi release by *LdPri* as a function of substrates NTP and DNA in the presence and absence of EGCG (IC_50_, 2× IC_50_) is shown in Figure 9E,G. The confidence intervals for *V*_max_ EGCG (IC_50_ and 2× IC_50_) for substrate NTP were found to be (31.14–32.05) μM/min (*R* ^2^ = 0.90) and (18.62–18.88) (*R* ^2^ = 0.85) μM/min, respectively, while *K*_m_ EGCG (IC_50_ and 2× IC_50_) for substrate NTP was found to be (7.61–11.39) μM (*R* ^2^ = 0.90) and (2.58–4.13) μM (*R* ^2^ = 0.85) respectively.

While the confidence intervals for *V*_max_ EGCG (IC_50_ and 2× IC_50_) for substrate DNA were found to be (31.50–32.40) μM/min (*R* ^2^ = 0.93) and (21.82–22.77) μM/min (*R* ^2^ = 0.84) respectively, while *K*_m_ Pritelivir (IC_50_ and 2× IC50) for substrate DNA was found to be (00.06–0.09) μM (*R* ^2^ = 0.93) and (0.05–0.009) μM (*R* ^2^ = 0.84) respectively.

The interaction of *LdPri* with EGCG is represented as (Cheng and Prusoff 1973; Brandt et al. 1987).

(9) $$\begin{array}{l} E+S⇄\mathrm{ES}\to E+P\\ +I\\ \begin{array}{c} ↓↑ \end{array}\\ \mathrm{ESI} \end{array}$$

*K*_*i*_ for uncompetitive inhibition by EGCG was calculated using Equation (10) (Cheng and Prusoff 1973; Brandt et al. 1987).

(10) $$K_{i}=\frac{I_{50}}{\left(K_{m}/S\right)+1}.$$

The *K*_*i*_ values for EGCG were 24.08 ± 0.11 and 22.68 ± 0.86 nM for the substrates NTP and DNA, respectively. For uncompetitive inhibition, *K*_*i*_ equals the IC_50_ as *S* increases with *K*_m_ (Brandt et al. 1987).

$$K_{i}=I_{50}\left(S>>K_{m}\;\text{for}\;\text{uncompetitive}\;\text{inhibitors}\right).$$

### 3.11 Parasite Inhibition Assay

This study aimed to determine the effects of EGCG, p‐coumaric acid, and Pritelivir on the vitality of *L. donovani* parasites. To this end, *L. donovani* promastigotes were incubated with different concentrations of EGCG, p‐Coumaric acid, and Pritelivir, along with the positive control, Amphotericin B. The inhibitor that elicited the most potent anti‐leishmanial activity was Pritelivir, which was superior to the control, Amphotericin B (Figure S22). Parasites' growth was primarily affected by Pritelivir in a dose‐dependent way with an IC_50_ value of 9.21 ± 0.26 μM (*R* ^2^ = 0.98; *p* = 0.0018) while Amphotericin B exhibited an IC_50_ value of 12.8 ± 0.75 μM (*R* ^2^ = 0.99; *p* = 0.0027). EGCG also exhibited an anti‐leishmanial effect, with an IC_50_ of 23.58 ± 3.94 μM (*R* ^2^ = 0.99, *p* = 0.0236), which was twofold higher than that of the control. The anti‐leishmanial effect of EGCG was found to be greater against *L. donovani* than against its counterpart, *L. infantum*, which is responsible for VL epidemiology (Inacio et al. 2021). P‐Coumaric acid exhibited a poor anti‐leishmanial effect with a fivefold higher value than the control, with an IC_50_ value of 59.37 ± 3.91 μM (*R* ^2^ = 0.99; *p* = 0.0624), comparable to previous works (Antwi et al. 2019).

### 3.12 Effects of Pritelivir and ECGC on Parasite Growth and Morphology of *L. donovani* Promastigotes

**FIGURE 10:**
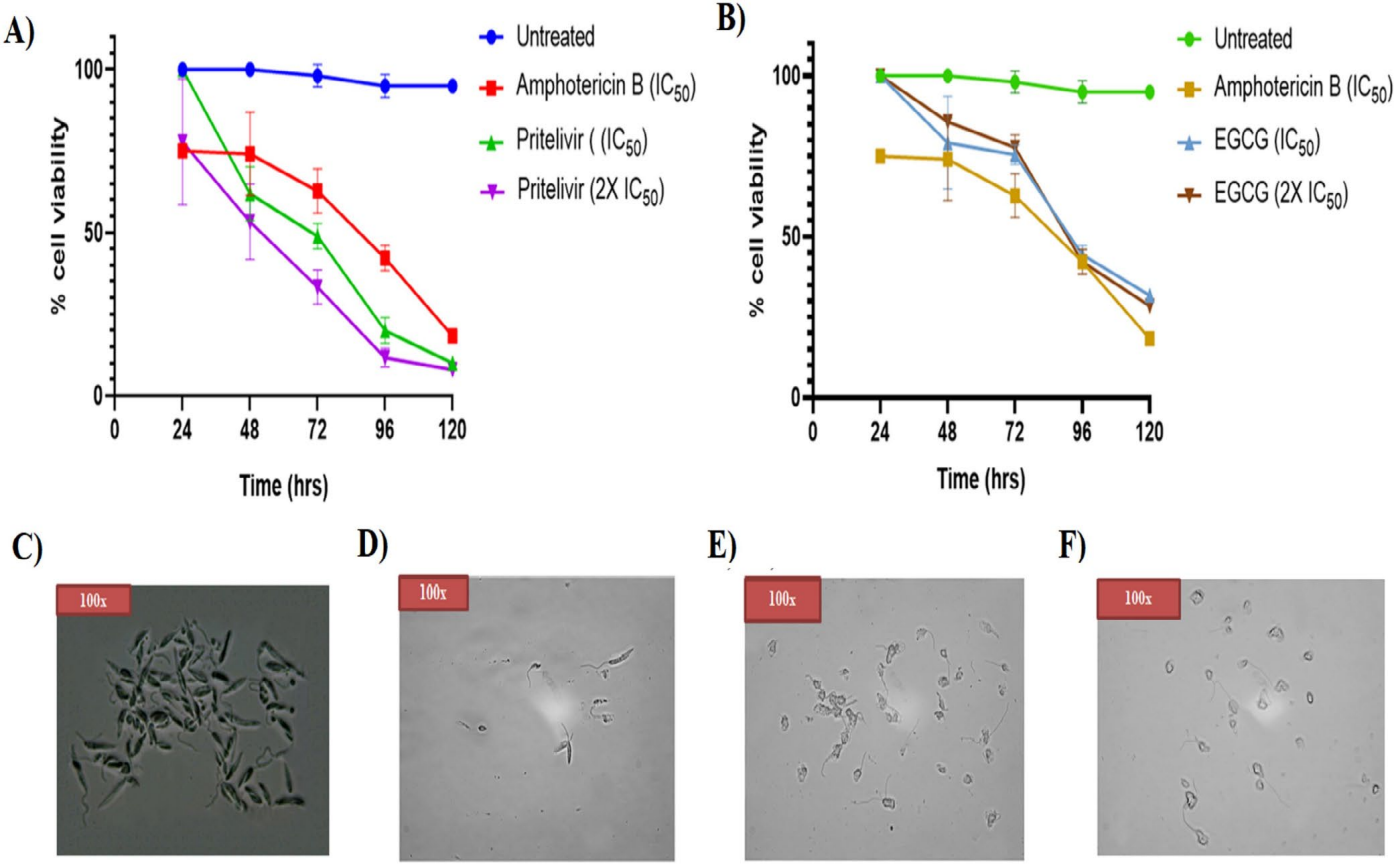
(A) Effect on the cell viability of *L. donovani* promastigotes treated with Pritelivir (IC_50_, 2× IC_50_) and Amphotericin B (IC_50_) for 120 h, (B) Effect on the cell viability of *L. donovani* promastigotes treated with EGCG (IC_50_, 2× IC_50_) and Amphotericin B (IC_50_) for 120 h, (C) Cell morphology of *L. donovani* promastigotes in the absence of inhibitor after 24 h under Phase Contrast Microscope (100×), (D) Effect on the cell morphology of *L. donovani* promastigotes treated with Pritelivir after 24 h under Phase Contrast Microscope (100×), (E) Effect on the cell morphology of *L. donovani* promastigotes treated with EGCG after 24 h under Phase Contrast Microscope (100×) and (F) Effect on the cell morphology of *L. donovani* promastigotes treated with Amphotericin B after 24 h under Phase Contrast Microscope (100×). All the experiments were done in triplicates. The bars indicate the standard deviation.

The effects of Pritelivir and EGCG on parasite growth were assessed by culturing promastigotes in the presence or absence of the inhibitors. The viable cells were checked every 24 h by trypan blue staining and counting using a hemocytometer, and were finally observed under a phase‐contrast microscope (100×). The replication of *L. donovani* promastigotes was significantly decreased at higher concentrations of Pritelivir (IC_50_, 2× IC_50_) and EGCG (IC_50_, 2× IC_50_) (Figure 10A,B). Untreated cells showed > 95% cell viability, while cell growth was reduced with Pritelivir (IC_50_, 2× IC_50_) as well as EGCG (IC_50_, 2× IC_50_) treatment at 48 and 24 h with Amphotericin B (IC_50_). At 72 h, promastigotes decreased by more than 50% in the presence of Pritelivir (IC_50_) (*p* = 0.023), and at 96 h with EGCG (IC_50_) (*p* = 0.0372). At 120 h, the promastigotes significantly decreased by 90% in the presence of Pritelivir (IC_50_) (*p* = 0.0127).

After 24 h, promastigotes treated with Pritelivir (IC_50_, 2× IC_50_), EGCG (IC_50_, 2× IC_50_), and Amphotericin B (IC_50_) exhibited morphological alterations. Abnormal changes in the size, shape, and integrity of promastigotes in treated cells were observed under a phase‐contrast microscope (100×) compared with untreated cells. 95% of untreated promastigotes had uniform cell surfaces, were slender and longer, and had an extended preserved flagellum (Figure 10C). Upon treatment with Pritelivir, the parasites showed irregular shapes and short flagella (Figure 10D). In contrast, EGCG‐ and Amphotericin B‐treated parasites exhibited rounded morphology and a distorted cell surface, with loss of flagella (Figure 10E,F).

## 4 Discussion

DNA primase of all the studied eukaryotes to date is a heterodimer of two subunits with the large regulatory (*PriL*) subunit and small catalytic (*PriS*) subunit of approximately 58 and 49 kDa in molecular weight, and generally associates strongly with the DNA polymerase α to form Prim‐Pol (Kuchta and Stengel 2010; Baranovskiy et al. 2016). Homologous heterodimeric primases are encoded in archaea and eukaryotes and harbor both *PriL* and *PriS* subunits, unlike bacterial DnaG. Recent research reveals that Prim‐Pol is essential for DNA repair (Wan et al. 2013). The present study has targeted the nuclear DNA primase of *L*. *donovani* (*LdPri*). In this study, we have biochemically characterized *LdPri* and evaluated it as a potential drug target to identify new treatment options for *L. donovani* infections.

*LdPri* activity was checked by a novel non‐radioactive primase‐pyrophosphatase assay developed by Biswas et al. (2013). It was a robust technique, and the reaction could be well performed at room temperature and interpreted. Thus, it did not require radiolabelled primers or other components, such as ssDNA‐binding or helicase proteins.

In the above study, we cloned, overexpressed, and purified both *LdPriL* and *LdPriS* using the His‐tag purification system (Figure S19A–C). *LdPri* activity could be observed on the ssM13 DNA template as a measure of absorbance using this novel non‐radioactive assay (Figure 7). *LdPri* activity was seen at maximum at a higher pH (20 mM of CAPS pH 9.0) and in the presence of the divalent cation Mg^2+^ (4 mM), probably due to the importance of Mg^2+^ ions in different physiological activities in the cell (Pilchova et al. 2017; Morais et al. 2017). The heat sensitivity of *LdPri* activity was also tested over a range of 40°C–80°C, with a 35% loss in activity at 80°C. While individual primase activity of *LdPriL* and *LdPriS* suggested *LdPriL* to be more efficient in primer synthesis on ssM13 DNA template than *LdPriS*, suggesting *LdPriS* to be unstable and inefficient without the association of *LdPriL* (Figure 7A–I).

Though *LdPriS* activity was poor, it could be measured in a plate reader, which accurately reflects its catalytic activity (Copeland and Tan 1995; Desogus et al. 1999; Kuchta and Stengel 2010). The catalytic subunit of DNA primase of all known crystal structures plays a vital role in the 3′‐terminal nucleotidyltransferase activity through the utilization of three negatively charged amino acids containing either aspartic acid or glutamic acid (Steitz et al. 1994). Site‐directed mutagenesis of the catalytic subunit (p50) in mouse (Copeland and Tan 1995) evaluated the conservation of residues GLU 105, ASP 109, and ASP 111, which are crucial for catalysis. It formed the motif (ELVFDID), which is particularly conserved in archaeal primases and *LdPriS*. It was considered for docking studies in our previous in silico studies (Bhowmik et al. 2021). Through MSA studies, we showed the sequence conservation of the ELVFDID with the three negatively charged residues GLU 162, ASP 166, and ASP 168, all well conserved in *LdPriS* (Figure S1B), along with our biochemical characterization study, indicated that the *LdPri* complex is equipped with 3′‐terminal nucleotidyltransferase activity in a non‐templated fashion (Figure 7I). However, its biological role remains poorly understood. The incorporation of individual dATP, dCTP, dGTP, and dTTP for *LdPri* nucleotidyltransferase activity testing indicated that primase initiation activity may be pyrimidine‐dependent, as in eukaryotic DNA primases (Kuchta and Stengel 2010). The exciting concept of *S. solfataricus* DNA primase, which uses poly‐pyrimidine single‐stranded DNA templates with low efficiency to de novo synthesize RNA primers in a non‐templated manner, contrasts with the 3′‐terminal nucleotidyltransferase activity of *LdPri* (De Falco et al. 2004). This terminal transferase activity is shared by DNA polymerases and DNA primase in prokaryotic, archeal, and eukaryotic organisms, as well as by reverse transcriptase from avian myeloblastosis virus (De Falco et al. 2004; Lao‐Sirieix and Bell 2004).

Zinc‐binding motifs include cysteine and histidine, which are necessarily conserved residues in several proteins involved in DNA and RNA recognition, but only a few primases from bacteria and bacteriophages that contain zinc atoms and conserved residues have been studied to date (Mendelman et al. 1994; Griep and Lokey 1996). The suggested Zn‐binding motif in the mouse DNA primase resides within 128–171 of the small catalytic subunit (p50) with sequence C X2C X35C X2C, where, in the case of *LdPriS*, the penultimate cysteine residue was replaced by a leucine residue (C X2C X35L X2C). In contrast, serine replaced the last cysteine in *Saccharomyces cerevisiae* (Figure S23). The first two cysteines in all available sequences are highly conserved in archaea, except in *Archaeoglobus fulgidus* (Desogus et al. 1999).

The Fe–S cluster of DNA primase is a redox switch involved in DNA replication and is responsible for the initiation of replication through DNA charge transport on single‐stranded DNA, which has been identified in proteins (Klinge et al. 2007; Weiner et al. 2007; Vaithiyalingam et al. 2010; O'Brien et al. 2017). While the catalytic subunit of the heteromeric DNA primase lacks an Fe–S cluster, the regulatory subunit contains one, which is required for efficient primer synthesis (Weiner et al. 2007; Pellegrini 2012). Previous reports on the crystal structure of the yeast ( *S. cerevisiae* ) primase suggested that yeast *PriL*‐CTD folds into two large, independent alpha‐helical domains, connected by a [4Fe–4S] cluster at their interface (Sauguet et al. 2010). The report also suggests conservation of a sequence between the eukaryotic *PriL*‐CTD and the C‐terminal region of archaeal folds around the Fe–S cluster, with four conserved cysteine residues (Klinge et al. 2007; Weiner et al. 2007). The Fe–S cluster of *PriL* from *S. cerevisiae* was characterized in *E. coli* , along with the evaluation of its crystal structure (PDB: 3LGB) (Sauguet et al. 2010). The Fe‐S cluster of yeast *PriL*‐CTD with residues CYS 336 and CYS 417 in the N‐terminal and CYS 434 and CYS 474 in the C‐terminal domain was found to be conserved in *LdPriL* (CYS 350, CYS 432, CYS 450, and CYS 502) upon sequence alignment (Figure S24). Moreover, superimposition between *LdPriL* and Crystal Structure of the Fe–S Domain of the yeast DNA primase (PDB: 3LGB) by PyMol evaluated a very low RMSD of 0.85 Å (Figure S25), which indicated high‐level similarity in structural and functional properties (Castrignanò et al. 2006; Gaur et al. 2018). Such primase activity shown by both the subunits of *LdPri* suggests that *LdPriL* and *LdPriS* may orient in cis form during primer synthesis and that the template‐primer is directed by both the functional domains situated on the same two subunit molecules (Baranovskiy et al. 2016).

Visceral leishmaniasis is the second most neglected potentially fatal protozoan disease caused by *L. donovani* parasites, and there is an urgent need for new potent drugs to overcome the resistance of the ongoing leishmanicidal drugs. Although several drugs are already approved and marketed, these drugs have severe adverse effects, such as abdominal pain, myalgia, anorexia, erythema, hypokalemia, diarrhea, and kidney failure. Apart from these adverse effects, new challenges have arisen in the form of drug resistance for VL. These parasites are developing resistance to most first‐line drugs for VL (Saha et al. 2021), creating a new challenge and an urgent need for drug discovery. In this study, we evaluated the *LdPri* complex as a potential drug target using in silico docking and a drug‐repurposing approach to identify a potential inhibitor. Also, the low similarity between *Leishmania* primase and its human counterpart makes it a suitable drug target. We found that EGCG, p‐Coumaric acid, and Pritelivir possess a higher binding affinity with the individual proteins, individual proteins in *the LdPri* complex, and the interacting sites of *the LdPri* complex (Tables S7–S9)—the outcome of MD simulations and binding free energy calculations via the MMPBSA approach for an additional 100 ns. Together with the results obtained from RMSD, RMSF, Rg, SASA, ED/PCA, and binding free energy analyses, we propose that EGCG, p‐Coumaric acid, and Pritelivir may act as potential inhibitors of *LdPri*, utilizing different sites and modes of action, and be further *examined* in vitro examinations for their proposed potential inhibitory activity.

In vitro, primase inhibition with purified *LdPri* proteins by non‐radioactive primase‐pyrophosphatase assay evaluated Pritelivir as the most efficient *LdPri* inhibitor with IC_50_ 6.85 ± 0.09 nM, followed by EGCG with IC_50_ 24.21 ± 3.6 nM (Figure 8). Nevertheless, the in vitro parasite inhibition assay using the MTT method showed Pritelivir as the most potent anti‐leishmanial drug, with greater growth inhibition than the control Amphotericin B (Figure S22). Pritelivir (AIC316, BAY 57‐1293) belongs to the class of anti‐HSV medications known as helicase‐primase inhibitors and is effective against HSV‐1 and HSV‐2 strains that are resistant to nucleoside analogs (Wald et al. 2016). The anti‐leishmanial effect of EGCG was found to be greater against *L. donovani* than against its counterpart, *L. infantum*, which is responsible for VL epidemiology (Inacio et al. 2021). p‐Coumaric acid exhibited a poor anti‐leishmanial effect, with a fivefold higher value than the control and an IC_50_ of 59.37 ± 3.91 μM, comparable to previous reports (Antwi et al. 2019). Moreover, Pritelivir was found to be a competitive inhibitor for NTP and DNA substrates. EGCG was evaluated as an uncompetitive inhibitor of the substrates NTP and DNA in an enzyme kinetics study (Table S15). At the same time, microscopic analysis revealed that Pritelivir was associated with the parasite's irregular shape and short flagellum. In contrast, EGCG and Amphotericin B were associated with rounded morphology, distorted cell membrane, and loss of flagellum.

The present study evaluated Pritelivir as a strong anti‐leishmanial for the first time by targeting the nuclear DNA primase of *L. donovani* . On the other hand, the previously reported anti‐leishmanial drug EGCG was also found to be effective against the nuclear DNA primase of *L. donovani* , with a lower IC_50_ than against *L. infantum* trypanothione reductase (TR), an essential enzyme of redox homeostasis (Inacio et al. 2021). Our findings shed light on the importance of drug repurposing and the potential of Pritelivir and EGCG to eradicate VL. Further, combination studies of the evaluated inhibitors with already established anti‐leishmanial and synergistic effects are still a long way off, but may provide more valuable information. The poor binding affinity of EGCG, p‐Coumaric acid, and Pritelivir toward both subunits of human DNA primase indicates a reduced likelihood of off‐target inhibition of the host enzyme, suggesting their potential suitability as selective inhibitors of Leishmania DNA primase. Further, in vivo studies with Pritelivir and EGCG for leishmanicidal activity are necessary for candidate evaluation.

## 5 Conclusion

By interacting with active‐site residues and exhibiting high binding energies to both *LdPriL* and *LdPriS* in the *LdPri* complex, Pritelivir, p‐Coumaric acid, and EGCG likely inhibit the replication machinery and developmental processes in *L. donovani* . Pritelivir, EGCG, and p‐Coumaric acid were found to stabilize at the binding sites of the *LdPri* protein during the 100 ns MD simulation for both subunits, and the MMPBSA free‐energy analysis suggests that EGCG and Pritelivir could be more effective. The current data also shed further light on how EGCG and Pritelivir work against *L. donovani* . Pritelivir was a competitive inhibitor, whereas EGCG was a non‐competitive inhibitor of the *LdPri* substrate. Our research indicates that EGCG and Pritelivir may both be useful treatments for eliminating VL in the future by targeting the nuclear DNA primase of *L. donovani* . The results indicate that Pritelivir and EGCG, though via different mechanisms of action, both inhibit the replication machinery of *L. donovani* . They may also affect the cell cycle, altering cell morphology and integrity. While our study demonstrates promising activity of the identified compounds against *Leishmania* promastigotes, the clinically relevant intracellular amastigote stage was not investigated. As drug efficacy and host–parasite interactions can differ significantly between parasite stages, this represents a limitation of our work. As our lab does not have the capacity to initiate amastigote cultures, future studies will be required to validate these findings in amastigote models and in mammalian cells for cytotoxicity studies to establish their therapeutic relevance. Further in vivo studies on Pritelivir, EGCG, and p‐Coumaric acid will certainly pave the way for new and effective anti‐leishmanial agents.

## Author Contributions

**Anupama Sharma:** methodology, investigation, formal analysis. **Deep Bhowmik:** investigation, writing – original draft, writing – review and editing, methodology, formal analysis. **Ravi Prakash Arya:** writing – review and editing, formal analysis. **Diwakar Kumar:** conceptualization, investigation, writing – review and editing, funding acquisition, supervision, methodology, writing – original draft, resources, formal analysis.

## Funding

This work was supported by the Anusandhan National Research Foundation (ANRF), India (File No. ECR/2015/000155), awarded to Dr. Diwakar Kumar.

## Ethics Statement

The authors have nothing to report.

## Consent

The authors have nothing to report.

## Conflicts of Interest

The authors declare no conflicts of interest.

## Supporting information


**Table S1:** Binding sites residues for protein–protein interaction of *LdPriL* and *LdPriS* derived from SPPIDER web server.
**Table S2:** Results of alignment study of important and functionally active site residues in yeast *PriL* with *LdPriL* for evaluation of active site in *LdPriL*.
**Table S3:** Sequence producing significant alignment against *LdPriL* and *LdPriS* through BLASTP (https://blast.ncbi.nlm.nih.gov/Blast.cgi?PAGE=Proteins).
**Table S4:** Statistics of the interaction between *LdPriL* and *LdPriS* by HADDOCK 2.4.
**Table S5:** Total energy difference of individual residues after in silico Alanine Scanning Mutagenesis by PPCheck for *LdPriL* active site residues after MSA studies.
**Table S6:** Total energy difference of individual residues after in silico Alanine Scanning Mutagenesis by PPCheck for *LdPriS* active site residues after MSA studies.
**Table S7:** Binding energy of the top 10 natural anti‐lesihmanial compounds against different hotspot sites of *LdPri* complex, *LdPriL*‐*LdPriS* interaction as well as individual proteins by PyRx along with their biological activity.
**Table S8:** Binding energy of the top 10 DNA/RNA synthesis inhibitors against different hotspot sites of *LdPri* complex, *LdPriL*‐*LdPriS* interaction as well as individual proteins by PyRx along with their biological activity.
**Table S9:** Binding energy of the top 10 protein–protein interaction inhibitors against different hotspot sites of *LdPri* complex, *LdPriL*‐*LdPriS* interaction as well as individual proteins by PyRx along with their biological activity.
**Table S10:** Key interaction of selected ligands with the hotspot residues at different hotspot sites of *LdPri* complex, *LdPriL*‐*LdPriS* interaction as well as individual proteins by PyRx.
**Table S11:** Contribution of individual energy components involved in complex formation between EGCG, p‐Coumaric acid and Pritelivir with the different hotspot sites of *LdPri* complex in **Set (1–3)**.
**Table S12:** Active sites for large and small subunit of Human DNA Primase selected for molecular docking.
**Table S13:** Binding energy of EGCG, p‐Coumaric acid and Pritelivir against the large and small subunits of Human DNA Primase by PyRx.
**Table S14:** Key interaction of EGCG, p‐Coumaric acid and Pritelivir against the large and small subunits of Human DNA Primase by Pymol.
**Table S15:** Inhibitiory effect of Pritelivir and EGCG on *LdPri* after enzyme kinetic study.
**Figure S1:** MSA by Clustal Omega for evaluating the conservation of residues (highlighted in green) and active site prediction in *L. donovani* DNA Primase (A) large sub‐unit (*LdPriL*) and (B) small sub‐unit (*LdPriS*).
**Figure S2:** (A) Ramachandaran plot of Model 2 for *LdPriL* modeling through PROCHECK, (B) ERRAT plot for the *L. donovani* DNA Primase large sub‐unit model. Black bars indicate distantly located misfolded region from the active site, gray bars demonstrate the error region between 95% and 99% and white bars indicate regions with lower rate protein folding, (C) ProSA‐web z‐scores of all protein chains in PDB which are determined by X‐ray crystallography (light blue) or NMR spectroscopy (dark blue) with respect to their length. The z‐score of our target protein is −9.5 and is highlighted with black dot, (D) ProSA‐web energy plot of *L. donovani* DNA Primase large sub‐unit model. Thick line demonstrates average energy over each 40 residue fragment. The thin line demonstrates the same with a smaller window size of 10 residues in the background of the plot.
**Figure S3:** (A) Ramachandaran plot of Model 4 for *LdPriS* modeling through PROCHECK, (B) ERRAT plot for the *L. donovani* DNA Primase small sub‐unit model. Black bars indicate distantly located misfolded region from the active site, gray bars demonstrate the error region between 95% and 99% and white bars indicate regions with lower rate protein folding, (C) ProSA‐web z‐scores of all protein chains in PDB which are determined by X‐ray crystallography (light blue) or NMR spectroscopy (dark blue) with respect to their length. The z‐score of our target protein is −5.71 and is highlighted with black dot, (D) ProSA‐web energy plot of *L. donovani* DNA Primase small sub‐unit model. Thick line demonstrates average energy over each 40 residue fragment. The thin line demonstrates the same with a smaller window size of 10 residues in the background of the plot.
**Figure S4:** Bar diagram of Total energy difference of predicted active site residues after *In silico* Alanine Scanning Mutagenesis by PPCheck for (A) *LdPriL* by GraphPad prism versus 8.0 and (B) *LdPriS* by GraphPad prism versus 8.0.
**Figure S5:** Diagrammatic reprsentation of the hotspot residues of *LdPriL* (blue and cartoon) and *LdPriS* (red and cartoon) in *LdPri* complex after in silico Alanine Scanning Mutagenesis selected for molecular docking. The hotspot residues of *LdPriL* are shown in green and sticks while hotspot residues of *LdPriS* are shown in yellow and sticks.
**Figure S6:** 2D H‐bond interactions of EGCG with *LdPriL* (left) and *LdPriL* in *LdPri* complex (right) by LigPlot+.
**Figure S7:** 2D H‐bond interactions of EGCG with *LdPriS* (left) and *LdPriS* in *LdPri* complex (right) by LigPlot+.
**Figure S8:** 2D H‐bond interactions of p‐Coumaric acid with *LdPriL* (left) and *LdPriL* in *LdPri* complex (right) by LigPlot+.
**Figure S9:** 2D H‐bond interactions of p‐Coumaric acid with *LdPriS* (left) and *LdPriS* in *LdPri* complex (right) by LigPlot+.
**Figure S10:** 2D H‐bond interactions of Pritelivir with *LdPriL* (left) and *LdPriL* in *LdPri* complex (right) by LigPlot+.
**Figure S11:** 2D H‐bond interactions of Pritelivir with *LdPriS* (left) and *LdPriS* in *LdPri* complex (right) by LigPlot+.
**Figure S12:** 2D H‐bond interactions of (A) EGCG, (B) p‐Coumaric acid, and (C) Pritelivir with interacting residues in *LdPri*complexbyLigPlot+.
**Figure S13:** (A) 3D representation of the H‐bond interaction of EGCG (orange and sticks) with Human DNA Primase large subunit (ruby and cartoon), (B) 3D representation of the H‐bond interaction of p‐Coumaric acid (black and sticks) with Human DNA Primase large subunit (ruby and cartoon), (C) No H‐bond interaction was observed for Pritelivir (green and sticks) against Human DNA Primase large subunit (ruby and cartoon), (D) No H‐bond interaction was observed for EGCG (orange and sticks) against Human DNA Primase small subunit (cyan and cartoon), (E) No H‐bond interaction was observed for p‐Coumaric acid (black and sticks) against Human DNA Primase small subunit (cyan and cartoon) and (F) 3D representation of the H‐bond interaction of Pritelivir (green and sticks) against Human DNA Primase small subunit (cyan and cartoon).
**Figure S14:** The stability and convergence ofEGCG, p‐Coumaric acid and Pritelivir within the active site of *LdPriL* in *LdPri* complex in **Set 1** during 0, 25, 50, 75, and 100 ns MD run along with the apo form showing the active site region (surface and black).
**Figure S15:** The stability and convergence ofEGCG, p‐Coumaric acid and Pritelivir within the active site of *LdPriS* in *LdPri* complex in **Set 2** during 0, 25, 50, 75, and 100 ns MD run along with the apo form showing the active site region (surface and black).
**Figure S16:** The stability and convergence ofEGCG, p‐Coumaric acid and Pritelivir within the interacting site between *LdPriL* and *LdPriS* in *LdPri* complex in **Set 3** during 0, 25, 50, 75, and 100 ns MD run along with the apo form showing the active site region (surface and black).
**Figure S17:** First two eigenvectors describing the projection of protein motion in phase space for EGCG, p‐Coumaric acid and Pritelivir in **Set (1–3)**.
**Figure S18:** Comparison of different mmpbsa calculated energies evaluated for EGCG, p‐Coumaric acid and Pritelivir against different hotspot sites in *LdPri* complex for **Set (1–3)**.
**Figure S19:** (A) Purification of overexpressed LdpriS‐pET‐28a(+) by Co^2+^‐NTA affinity chromatography. (B) Purification of overexpressed LdpriL‐pASK‐IBA43plus by Co^2+^‐NTA affinity chromatography, and (C) Quantification of recombinant *LdPriL* and *LdPriS* by Bradford assay.
**Figure S20:** (A) Western blot analysis of 6× His‐tagged Monoclonal antibody (NovagenR) using varying amount of purified recombinant *LdPriL* as Ag. Lane 1: 1 ng of Ag‐protein, Lane 2: 5 ng of Ag‐protein, Lane 3: 10 ng of Ag‐protein, Lane 4: 20 ng of Ag‐protein, Lane 5: 30 ng of Ag‐protein, Lane 6: 50 ng of Ag‐protein and Lane 7: 100 ng of Ag‐protein; (B) Western blot analysis of 6× His‐tagged Monoclonal antibody (NovagenR) using varying amount of purified recombinant *LdPriS* as Ag. Lane 1: 100 ng of Ag‐protein, Lane 2: 50 ng of Ag‐protein, Lane 3: 30 ng of Ag‐protein, Lane 4: 20 ng of Ag‐protein, Lane 5: 10 ng of Ag‐protein, Lane 6: 5 ng of Ag‐protein and Lane 7: 1 ng of Ag‐protein, Lane8: 0.5 ng of Ag‐protein; (C) Determination of titer of 6× His‐tagged Monoclonal antibody by ELISA. The titer of 6× His‐tagged Monoclonal antibody decreases with the increase of dilutions which suggests the purity of our recombinant *LdPriL* and *LdPriS*.
**Figure S21:** Optimization of *LdPri* activity by primase‐pyrophosphatase assay in a 96‐well microplate under different conditions and parameters.
**Figure S22:** (A) Dose–response plot for Amphotericin B, Pritelivir, EGCG and p‐Coumaric acid from MTT assay studies in 96‐well clear polystyrene plates and (B) MTT assay in 96‐well clear polystyrene plates evaluating *L. donovani* parasite inhibition at different concentrations of anti‐leishmanials—Lane1 (right panel): Amphotericin B (control) treated parasites; Lane 2, 3, 4 (right panel): EGCG treated parasites; Lane 5, 6, 7 (right panel): p‐Coumaric acid treated parasites; Lane 8, 9, 10 (right panel): Pritelivir treated parasites. Left panel: Controls and 0.1% DMSO treated cells with purple color indicating no inhibition. All the experiments were done in triplicates. The bars indicate the standard deviation.
**Figure S23:** MSA by Clustal Omega for evaluating the conservation of Zn‐binding motif residues in *L. donovani* DNA Primase small sub‐unit (*LdPriS*). The conserved cysteine residues are highlighted in green. The penultimate cysteine residue was replaced by a leucine residue in *L. donovani* and is highlighted in red.
**Figure S24:** MSA by Clustal Omega for evaluating the conservation of Fe‐S cluster in *L. donovani* DNA Primase large sub‐unit (*LdPriL*). The conserved cysteine residues are highlighted in green.
**Figure S25:** Protein–protein superimposition of *LdPriL*(pink) and *Crystal structure of* Fe‐S Domain of the yeast DNA primase (PDB: 3LGB) (yellow) by Pymol software.

## Data Availability

All data generated or analyzed during this study are included in this article (and its Supporting Information files).
