# Supplementary material for: Elucidation of DNA Primase as a Drug Target in Leishmania donovani: Structure‐Guided Inhibitor Identification and Biochemical Validation
Source: Chem Biol Drug Des. 2026 Jul 25;108(1):e70362. doi: 10.1111/cbdd.70362 (PMC13401365; doi:10.1111/cbdd.70362)
Supplement: Supplementary file 1 — Table S1: Binding sites residues for protein–protein interaction of LdPriL and LdPriS derived from SPPIDER web server. Table S2: Results of alignment study of important and functionally active site residues in yeast PriL with LdPriL for evaluation of active site in LdPriL. Table S3: Sequence producing significant alignment against LdPriL and LdPriS through BLASTP (https://blast.ncbi.nlm.nih.gov/Blast.cgi?PAGE=Proteins). Table S4: Statistics of the interaction between LdPriL and LdPriS by HADDOCK 2.4. Table S5: Total energy difference of individual residues after in silico Alanine Scanning Mutagenesis by PPCheck for LdPriL active site residues after MSA studies. Table S6: Total energy difference of individual residues after in silico Alanine Scanning Mutagenesis by PPCheck for LdPriS active site residues after MSA studies. Table S7: Binding energy of the top 10 natural anti‐lesihmanial compounds against different hotspot sites of LdPri complex, LdPriL‐LdPriS interaction as well as individual proteins by PyRx along with their biological activity. Table S8: Binding energy of the top 10 DNA/RNA synthesis inhibitors against different hotspot sites of LdPri complex, LdPriL‐LdPriS interaction as well as individual proteins by PyRx along with their biological activity. Table S9: Binding energy of the top 10 protein–protein interaction inhibitors against different hotspot sites of LdPri complex, LdPriL‐LdPriS interaction as well as individual proteins by PyRx along with their biological activity. Table S10: Key interaction of selected ligands with the hotspot residues at different hotspot sites of LdPri complex, LdPriL‐LdPriS interaction as well as individual proteins by PyRx. Table S11: Contribution of individual energy components involved in complex formation between EGCG, p‐Coumaric acid and Pritelivir with the different hotspot sites of LdPri complex in Set (1–3). Table S12: Active sites for large and small subunit of Human DNA Primase selected for molecular docking. Tab [file CBDD-108-e70362-s001.doc]

**Supplementary data**

**“Elucidation of DNA primase as a drug target in *Leishmania donovani*: structure-guided inhibitor identification and biochemical validation.”**

**Supplementary Table**

**Table S1.** Binding sites residues for protein-protein interaction of *LdPriL* and *LdPriS* derived from SPPIDER web server.

| **Proteins** | **Binding site residues** |
| --- | --- |
| *LdPriL* | 31, 43, 44, 45, 49, 93, 104, 106, 107, 108, 109, 111, 112, 113, 114, 115, 116, 117, 118, 121, 122, 135, 198, 199, 223, 227, 230, 231, 243, 244, 247, 250, 251, 253, 254, 257, 259, 260, 261, 287, 292, 293, 295, 296, 298, 299, 301, 304, 319, 320, 321, 330, 366, 370, 402, 406, 407, 412, 415, 416, 419, 469, 470, 471, 472, 473, 474, 531 |
| *LdPriS* | 16, 17, 18, 19, 20, 21, 22, 23, 25, 52, 53, 54, 55, 56, 57, 58, 59, 60, 61, 62, 63, 64, 65, 66, 67, 68, 69, 70, 71, 73, 74, 109, 110, 111, 112, 113, 114, 115, 116, 149, 150, 152, 153, 154, 155, 156, 157, 255, 264, 266, 269, 270, 274, 277, 287, 407, 417, 420, 421, 423, 424, 425, 426, 427, 430 |

**Table S2.** Results of alignment study of important and functionally active site residues in yeast *PriL* with *LdPriL* for evaluation of active site in *LdPriL.*

| **Active site residues in yeast *PriL*** | **Residues Aligned with *LdPriL* after MSA** | **Conservation Status** | **Assigned residual activity in yeast** | **Reference** |
| --- | --- | --- | --- | --- |
| HIS 349 | HIS 363 | Conserved | Conserved basic and polar residues that could interact with the phosphate backbone of template DNA and the substrate ribonucleotides | Sauguet et al., 2010 |
| ARG 351 | LYS 365 | Non-conserved | Conserved basic and polar residues that could interact with the phosphate backbone of template DNA and the substrate ribonucleotides |
| TYR 352 | HIS 366 | Non-conserved | Solvent exposed residue providing points of contact through aromatic stacking with the bases of the DNA template |
| ARG 355 | ARG 369 | Conserved | Potential interaction with both RNA nucleotides and template DNA. |
| LYS 363 | LYS 377 | Conserved | Architectural role via multiple polar interactions residues ASN 402 and TYR 412 and side-chain amide function of ASN 411, thus fixing the conformation of the putative DNA-binding loop. |
| LYS 390 | ALA 404 | Non-conserved | Conserved basic and polar residues that could interact with the phosphate backbone of template DNA and the substrate ribonucleotides |
| LYS 393 | THR 408 | Non-conserved | Conserved basic and polar residues that could interact with the phosphate backbone of template DNA and the substrate ribonucleotides |
| TYR 395 | TYR 410 | Conserved | Solvent exposed residue providing points of contact through aromatic stacking with the bases of the DNA template |
| TYR 397 | TYR 412 | Conserved | Solvent exposed residue providing points of contact through aromatic stacking with the bases of the DNA template |
| HIS 401 | HIS 416 | Conserved | Missense mutation of HIS 401 is lethal in yeast. Conserved basic and polar residues that could interact with the phosphate backbone of template DNA and the substrate ribonucleotides |
| ASN 402 | ASN 417 | Conserved | Polar interactions with residue LYS 363 fixing the conformation of the putative DNA-binding loop. |
| ASN 411 | SER 426 | Non-conserved | Polar interactions with residue LYS 363 fixing the conformation of the putative DNA-binding loop. |
| TYR 412 | TYR 427 | Conserved | Solvent exposed residue providing points of contact through aromatic stacking with the bases of the DNA template |
|  |  |  |  |  |

**Table S3.** Sequence producing significant alignment against *LdPriL* and *LdPriS* through BLASTP (<https://blast.ncbi.nlm.nih.gov/Blast.cgi?PAGE=Proteins>)

|  | *LdPriL* | *LdPriS* |
| --- | --- | --- |
| Description | Chain B, Crystal Structure Of Human Primase | Chain A, Crystal Structure Of The Catalytic Subunit Of Human Primase |
| Max Score | 159 | 199 |
| Total Score | 159 | 199 |
| Query cover | 64% | 72% |
| E-value | 2e-42 | 2e-59 |
| Similarity | 31% | 34% |
| Accession | 4RR2_B | 4LIK |

**Table S4.** Statistics of the interaction between *LdPriL* and *LdPriS* by HADDOCK 2.4

|  | **cluster 1** | **Cluster 2** |
| --- | --- | --- |
| HADDOCK score (kcal/mol) | − 200.1 + / − 26.0 | 31.7 +/-13.5 |
| Cluster size | 8 | 14 |
| RMSD from the overall lowest-energy structure (Å) | 10.9 +/-0.5 | 12.2 +/-0.1 |
| Van der Waals energy (kcal/mol) | -28.9 +/-10.0 | -57.8 +/-8.2 |
| Electrostatic energy (kcal/mol) | -385.3 +/-5.6 | -246.4 +/-34.7 |
| Desolvation energy (kcal/mol) | -17.3 +/-6.1 | -17.3 +/-2.0 |
| Restraints violation energy (kcal/mol) | 92.9 +/-38.9 | 1560.8 +/-42.4 |
| Buried Surface Area (A^2^) | 2100.5 + / − 93.6 Å^2^ | 1799.2 +/-202.1 |
| Z-Score | -2.3 | 1.0 |

**Table S5.** Total energy difference of individual residues after *In silico* Alanine Scanning Mutagenesis by PPCheck for *LdPriL* active site residues after MSA studies.

| **Residue before mutation** | **Residue after mutation** | **Total energy before mutation (kJ/mol)** | **Total energy after mutation (kJ/mol)** | **Energy Difference** |
| --- | --- | --- | --- | --- |
| HIS 363 | ALA 363 | -465.41 | -435.06 | 30.45 |
| LYS 365 | ALA 365 | -465.41 | -425.62 | 39.79 |
| HIS 366 | ALA 366 | -465.41 | -453.74 | 11.67 |
| ARG 369 | ALA 369 | -465.41 | -451.88 | 13.53 |
| LYS 377 | ALA 377 | -465.41 | -444.98 | 20.43 |
| ALA 404 | ALA 404 | -465.41 | -465.41 | 0 |
| THR 408 | ALA 408 | -465.41 | -450.06 | 15.35 |
| TYR 410 | ALA 410 | -465.41 | -457.18 | 8.23 |
| TYR 412 | ALA 412 | -465.41 | -465.41 | 0 |
| HIS 416 | ALA 416 | -465.41 | -465.43 | -0.02 |
| ASN 417 | ALA 417 | -465.41 | -458.41 | 7.00 |
| SER 426 | ALA 426 | -465.41 | -466.16 | -0.75 |
| TYR 427 | ALA 427 | -465.41 | -459.44 | 5.97 |

**Table S6.** Total energy difference of individual residues after *In silico* Alanine Scanning Mutagenesis by PPCheck for *LdPriS* active site residues after MSA studies.

| **Residue before mutation** | **Residue after mutation** | **Total energy before mutation (kJ/mol)** | **Total energy after mutation (kJ/mol)** | **Energy Difference** |
| --- | --- | --- | --- | --- |
| GLU 162 | ALA 162 | -699.28 | -679.93 | 19.35 |
| LEU 163 | ALA 163 | -699.28 | -681.93 | 17.35 |
| VAL 164 | ALA 164 | -699.28 | -693.57 | 5.71 |
| PHE 165 | ALA 165 | -699.28 | -682.44 | 16.84 |
| ASP 166 | ALA 166 | -699.28 | -666.68 | 32.60 |
| ILE 167 | ALA 167 | -699.28 | -696.79 | 2.49 |
| ASP 168 | ALA 168 | -699.28 | -673.03 | 26.25 |
| ASP 171 | ALA 171 | -699.28 | -695.48 | 3.80 |
| ARG 219 | ALA 219 | -699.28 | -696.64 | 2.64 |
| ARG 220 | ALA 220 | -699.28 | -668.80 | 30.48 |

**Table S7.** Binding energy of the top 10 natural anti-lesihmanial compounds against different hotspot sites of *LdPri* complex, *LdPriL*-*LdPriS* interaction as well as individual proteins by PyRx along with their biological activity

| **Ligands** | **Binding energy against (kcal/mol)** | | | | | **Biological activity** |
| --- | --- | --- | --- | --- | --- | --- |
|  | ***LdPri*L (unbound) (kcal/mol)** | ***LdPriL* in *LdPri* complex (kcal/mol)** | ***LdPriS* (unbound) (kcal/mol)** | ***LdPriS* in *LdPri* complex (kcal/mol)** | ***LdPriL*-*LdPriS* interaction (kcal/mol)** |  |
| EGCG | -8.8 | -8.9 | -8.5 | -8.5 | -7.7 | potent antioxidant, anti-inflammatory, cardioprotective, and antitumor properties (Narotzki et al., 2012) |
| p-Coumaric acid | -7.8 | -7.8 | -7.2 | -7.2 | -6.2 | antimelanogenic effects (An et al., 2010) |
| Apigenin | -6.5 | -6.0 | -6.2 | -5.9 | -3.9 | antioxidant, anti-inflammatory, blood pressure reduction, and chemo-preventive (Shankar et al., 2017) |
| Caffeic acid | -6.4 | -5.4 | -6.8 | -6.0 | -1.7 | anti-hepatocarcinoma activity (Aysegul, 2017) |
| Rosamarinic acid | -6.0 | -5.2 | -6.3 | -6.1 | -1.3 | anti-inflammatory, antioxidant, antidepression, and antiallergy properties (Al-Sereiti et al., 1999; Zheng & Wang, 2001; Takeda et al., 2002; Ito  et al., 1998) |
| Ferulic acid | -5.9 | -5.4 | -6.2 | -5.8 | -0.9 | antioxidant, anti-inflammatory, antimicrobial, and anticancer properties (Mancuso & Santangelo, 2014; Pyrzynska, 2024) |
| Diplacone | -5.5 | -5.2 | -6.0 | -5.9 | +0.8 | anti-inflammatory (Hošek et al., 2010; Hošek et al., 2013), antiradical, cytoprotective 76 (Šmejkal et al., 2008; Zima et al., 2010), and antibacterial activities ( Navrátilová et al., 2013) |
| Amentoflavone | -6.3 | -6.3 | -5.5 | -5.4 | +2.3 | anti-inflammatory ([Tordera et al., 1994](https://pmc.ncbi.nlm.nih.gov/articles/PMC8727548/#B169); [Kim et al., 1998](https://pmc.ncbi.nlm.nih.gov/articles/PMC8727548/#B83); [Oh et al., 2013](https://pmc.ncbi.nlm.nih.gov/articles/PMC8727548/#B128); [An et al., 2016](https://pmc.ncbi.nlm.nih.gov/articles/PMC8727548/#B5); [Cai et al., 2019](https://pmc.ncbi.nlm.nih.gov/articles/PMC8727548/#B19)), antibacterial ([Hwang et al., 2013](https://pmc.ncbi.nlm.nih.gov/articles/PMC8727548/#B64)), antifungal ([Jung et al., 2006](https://pmc.ncbi.nlm.nih.gov/articles/PMC8727548/#B74); [Jung et al., 2007](https://pmc.ncbi.nlm.nih.gov/articles/PMC8727548/#B73); [Hwang et al., 2012](https://pmc.ncbi.nlm.nih.gov/articles/PMC8727548/#B63)), antivirus ([Lin et al., 1997](https://pmc.ncbi.nlm.nih.gov/articles/PMC8727548/#B112); [Wilsky et al., 2012](https://pmc.ncbi.nlm.nih.gov/articles/PMC8727548/#B185); [Coulerie et al., 2013](https://pmc.ncbi.nlm.nih.gov/articles/PMC8727548/#B41)), anti-oxidative ([Bonacorsi et al., 2012](https://pmc.ncbi.nlm.nih.gov/articles/PMC8727548/#B14); [Li et al., 2020](https://pmc.ncbi.nlm.nih.gov/articles/PMC8727548/#B111)), anti-angiogenesis ([Guruvayoorappan and Kuttan, 2008c](https://pmc.ncbi.nlm.nih.gov/articles/PMC8727548/#B55); [Tarallo et al., 2011](https://pmc.ncbi.nlm.nih.gov/articles/PMC8727548/#B166); [Zhang et al., 2014](https://pmc.ncbi.nlm.nih.gov/articles/PMC8727548/#B196)), neuroprotection ([Cao et al., 2017](https://pmc.ncbi.nlm.nih.gov/articles/PMC8727548/#B22); [Chen et al., 2018](https://pmc.ncbi.nlm.nih.gov/articles/PMC8727548/#B27); [Rong et al., 2019](https://pmc.ncbi.nlm.nih.gov/articles/PMC8727548/#B148); [Zhao et al., 2019](https://pmc.ncbi.nlm.nih.gov/articles/PMC8727548/#B201); [Cao et al., 2021](https://pmc.ncbi.nlm.nih.gov/articles/PMC8727548/#B21)), osteogenesis ([Zha et al., 2016](https://pmc.ncbi.nlm.nih.gov/articles/PMC8727548/#B194)), anti-arthritis ([Bais et al., 2017](https://pmc.ncbi.nlm.nih.gov/articles/PMC8727548/#B10); [Vasconcelos et al., 2019](https://pmc.ncbi.nlm.nih.gov/articles/PMC8727548/#B175)), radioprotection ([Park et al., 2011](https://pmc.ncbi.nlm.nih.gov/articles/PMC8727548/#B133); [Xu et al., 2014](https://pmc.ncbi.nlm.nih.gov/articles/PMC8727548/#B188); [Qu et al., 2019](https://pmc.ncbi.nlm.nih.gov/articles/PMC8727548/#B141)), antidiabetic ([Qin et al., 2018](https://pmc.ncbi.nlm.nih.gov/articles/PMC8727548/#B140); [Su et al., 2019](https://pmc.ncbi.nlm.nih.gov/articles/PMC8727548/#B163)) and antidepressant ([Ishola et al., 2012](https://pmc.ncbi.nlm.nih.gov/articles/PMC8727548/#B66)) |
| Robustoflavone | -5.8 | -5.7 | -6.1 | -5.8 | +4.5 | anti-inflammatory (Jo et al., 2019; antioxidant, anticancer, antimicrobial properties (Ververidis et al., 2007) |
| Colchicoside | -5.5 | -5.5 | -5.8 | -5.2 | +6.3 | muscle relaxation, analgesic effects, and anti-spasmodic properties (Mustafa et al., 2024) |

**Table S8.** Binding energy of the top 10 DNA/RNA synthesis inhibitors against different hotspot sites of *LdPri* complex, *LdPriL*-*LdPriS* interaction as well as individual proteins by PyRx along with their biological activity

| **Ligands** | **Binding energy against (kcal/mol)** | | | | | **Biological activity** |
| --- | --- | --- | --- | --- | --- | --- |
|  | ***LdPri*L (unbound) (kcal/mol)** | ***LdPriL* in *LdPri* complex (kcal/mol)** | ***LdPriS* (unbound) (kcal/mol)** | ***LdPriS* in *LdPri* complex (kcal/mol)** | ***LdPriL*-*LdPriS* interaction (kcal/mol)** |  |
| Pritelivir | -8.2 | -8.1 | -8.5 | -8.2 | -6.1 | Antiviral (Aoki, 2015) |
| Pyridostatin | -5.8 | -5.2 | -8.2 | -7.7 | -2.0 | Anti-tumoural activity ([Groelly](https://pubmed.ncbi.nlm.nih.gov/?term="Groelly FJ"%5BAuthor%5D) et al., 2022) |
| Cidofovir | -7.8 | -7.7 | -6.6 | -6.4 | +1.8 | Antiviral ([Hitchcoc](https://journals.sagepub.com/doi/abs/10.1177/095632029600700301#con1)k et al., 1996; Andrei & Snoeck, 2010) |
| Raltitrexed | -5.2 | -5.0 | -6.4 | -6.0 | +3.2 | antitumour agent (Gunasekara & Faulds, 2012) |
| Floxuridine | -5.0 | -4.9 | -6.2 | -6.0 | +3.8 | Anti-tumour (Morihiro et al., 2021), antibacterial (Yeo et al., 2018; Li et al., 2022; Li et al., 2023) |
| Gemcitabine | -7.1 | -6.2 | -6.0 | -5.8 | +5.5 | Anti-tumour (van Moorsel et al., 1997; Kroep et al., 2006) |
| Fludarabine | -6.8 | -6.3 | -6.1 | -5.4 | +7.4 | clinical treatment of lymphocytic hematologic malignancies (Plunkett et al., 1990) |
| Amenamevir | -6.0 | -5.1 | -5.8 | -5.2 | +7.6 | Anti viral (Shiraki et al., 2021) |
| Triazavirin | -6.0 | -5.5 | -5.3 | -5.1 | +7.9 | Antiviral (Loginova et al., 2007; Karpenko et al., 2010; Kiselev et al., 2012; Deyeva et al., 2014; Tikhonova  et al., 2018) |
| [Clofarabine](https://www.selleckchem.com/products/Clofarabine.html) | -5.2 | -5.1 | -6.3 | -5.4 | +8.2 | Anti-cancer (Zhenchuk et al., 2009; [Ghanem](https://www.tandfonline.com/author/Ghanem%2C+Hady) et al., 2010) |

**Table S9.** Binding energy of the top 10 Protein-Protein interaction inhibitors against different hotspot sites of *LdPri* complex, *LdPriL*-*LdPriS* interaction as well as individual proteins by PyRx along with their biological activity

| **Ligands** | **Binding energy against (kcal/mol)** | | | | | **Biological activity** |
| --- | --- | --- | --- | --- | --- | --- |
|  | ***LdPri*L (unbound) (kcal/mol)** | ***LdPriL* in *LdPri* complex (kcal/mol)** | ***LdPriS* (unbound) (kcal/mol)** | ***LdPriS* in *LdPri* complex (kcal/mol)** | ***LdPriL*-*LdPriS* interaction (kcal/mol)** |  |
| Pritelivir | -8.2 | -8.1 | -8.7 | -8.5 | -6.1 | Antiviral (Aoki, 2015) |
| Raltitrexed | -5.2 | -5.0 | -6.4 | -6.0 | +3.2 | antitumour agent (Gunasekara & Faulds, 2012) |
| Ibrutinib | -6.2 | -5.4 | -6.7 | -5.1 | +4.8 | Anti-cancer (Davids & Brown, 2014) |
| Amenamevir | -5.5 | -5.2 | -6.5 | -5.4 | +6.6 | Anti viral (Shiraki et al., 2021) |
| Mefenamic acid | -6.0 | -5.3 | -5.9 | -5.7 | +7.1 | non-steroidal anti-inflammatory drug (Naithani et al., 2025) |
| Dorzagliatin | -5.8 | -5.4 | -5.8 | -5.2 | +7.7 | a glucokinase activator o treat Type 2 diabetes (Jiang et al., 2024) |
| Ursodiol | -6.1 | -5.8 | -5.2 | -4.8 | +12.7 | Anti-inflammatory, anti-apoptotic, Choleretic, Immune modulation, Alteration of bile acid pool, Cell signaling, Mitochondrial integrity (Lazaridis et al., 2001) |
| Sulfadoxine | -5.6 | -5.1 | -5.9 | -4.7 | +15.8 | broad-spectrum antibiotic activity (Gutman et al., 2012) |
| Lopinavir | -5.6 | -5.0 | -5.4 | -4.2 | +16.2 | protease inhibitor with high specificity for HIV-1 protease (Hurst a Faulds, 2000); Anti-leishmanial (Rebello et al., 2018); Antifungal agent (Santos et al., 2021) |
| Grazoprevir | -5.2 | -4.8 | -5.9 | -5.6 | +20.4 | inhibitor of hepatitis C virus NS3/4A protease (Shah et al., 2014) |

**Table S10.** Key interaction of selected ligands with the hotspot residues at different hotspot sites of *LdPri* complex, *LdPriL*-*LdPriS* interaction as well as individual proteins by PyRx.

| **Ligands** | **Key interactions with *Ld-PriL* (unbound)** | **Key interactions with *Ld-PriL* in *Ld-Pri*complex** | **Key interactions with *Ld-PriS* (unbound)** | **Key interactions with *Ld-PriS* in *Ld-Pri*complex** | **Key interactions with *LdPriL (*chain A)-*LdPriS* (chain B)interacting residues** |
| --- | --- | --- | --- | --- | --- |
| EGCG | His 363 | Tyr 410 | Asp 166, Asp168, ARG 220 | Asp 166 | ARG 248 (A) |
| p-Coumaric acid | His 366 | Lys 365 | Asp 166, ASP 171 | ASP 171 | ARG 248 (A) |
| Pritelivir | Thr 408, Tyr 427 | His 366 | Asp 168 | Asp 166 | SER 56 (B), ASP 58 (B) |

**Table S11. Contribution of individual energy components involved in complex formation between EGCG, p-Coumaric acid and Pritelivir with the different hotspot sites of *LdPri* complex in** Set (1-3)

| **MMPBSA CALCULATION** | | ***ΔE_binding_* (kJ/mol)** | ***ΔE_vdw_*(kJ/mol)** | ***ΔE_elec_*(kJ/mol)** | ***ΔE_polar_*(kJ/mol)** | **SASA (kJ/mol)** |
| --- | --- | --- | --- | --- | --- | --- |
| **Set 1** | EGCG | --43.980 | -127.346 | -113.571 | 212.542 | -15.604 |
|  | p-Coumaric acid | -49.847 | -62.237 | -33.703 | 53.264 | -7.170 |
|  | Pritelivir | -112.539 | -231.943 | -49.316 | 189.661 | -20.940 |
| **Set 2** | EGCG | -1.013 | -120.517 | -163.589 | 301.236 | -18.142 |
|  | p-Coumaric acid | 15.718 | -0.010 | 0.342 | 15.587 | 0.000 |
|  | Pritelivir | -25.330 | -58.314 | -17.745 | 58.548 | -7.819 |
| **Set 3** | EGCG | -78.556 | -162.708 | -213.043 | 316.348 | -19.164 |
|  | p-Coumaric acid | -31.647 | -83.908 | -70.216 | 133.258 | -10.781 |
|  | Pritelivir | -21.519 | -210.081 | -96.736 | 307.286 | -21.989 |

**Table S12.** Active sites for large and small subunit of Human DNA Primase selected for molecular docking.

| **Proteins** | **Acitive site residues** | |
| --- | --- | --- |
|  | **Conserved Sequences** | Fe-S cluster |
| Human DNA Primase large subunit | HIS 300, HIS 303, ARG 306, LYS 314, TYR 345, TYR 347, HIS 351, SER 352, TYR 362 | CYS 287, CYS 367, CYS 384, CYS 424 |
| Human DNA Primase small subunit | GLU 109, LEU 110, VAL 111, PHE 112, ASP 113, ILE 114, ASP 115, ARG 166, ARG 167, GLY 168 |  |

Table S13. Binding energy of EGCG, p-Coumaric acid and Pritelivir against the large and small subunits of Human DNA Primase by PyRx.

| **Ligands** | **Binding energy against (kcal/mol) large subunit of Human DNA Primase** | **Binding energy against (kcal/mol) small subunits of Human DNA Primase** |
| --- | --- | --- |
| EGCG | -4.0 | +1.9 |
| p-Coumaric acid | -4.8 | +1.2 |
| Pritelivir | +2.7 | -3.2 |

**Table S14.** Key interaction of EGCG, p-Coumaric acid and Pritelivir against the large and small subunits of Human DNA Primase by Pymol.

| **Ligands** | **Key interactions with large subunit of Human DNA Primase** | **Key interactions with small subunits of Human DNA Primase** |
| --- | --- | --- |
| EGCG | GLY 310, SER 373 | No H-bond interaction |
| p-Coumaric acid | GLY 305, TRP 327 | No H-bond interaction |
| Pritelivir | No H-bond interaction | THR 117, SER 164 |

**Table S15.** Inhibitiory effect of Pritelivir and EGCG on *LdPri* after enzyme kinetic study.

| **Compound** | ***V_max_* (µM /min) for substrate NTP** | ***K_m_* (µM) for substrate NTP** | ***V_max_* (µM /min) for substrate DNA** | ***K_m_* (µM) for substrate DNA** | ***K_i_* (nM) for substrate NTP** | ***K_i_* (nM) for substrate DNA** | **Type of inhibition** |
| --- | --- | --- | --- | --- | --- | --- | --- |
| No inhibitor | 34.48 | 14.07 ± 3.22 | 37.98 ± 0.82 | 0.1 ± 0.02 | No inhibition | No inhibition | No inhibition |
| Pritelivir | 35.30 ± 0.71 | 42.95 ± 2.61 | 39.56 ± 2.34 | 0.54 ± 0.05 | 1.41 ± 1.01 | 2.21 ± 0.83 | Competitive |
| EGCG | 31.25 | 0.7± 0.12 | 31.26± 0.97 | 0.07± 0.03 | 24.08 ± 0.11 | 22.68 ± 0.86 | Uncompetitive |

**Supplementary Figure**


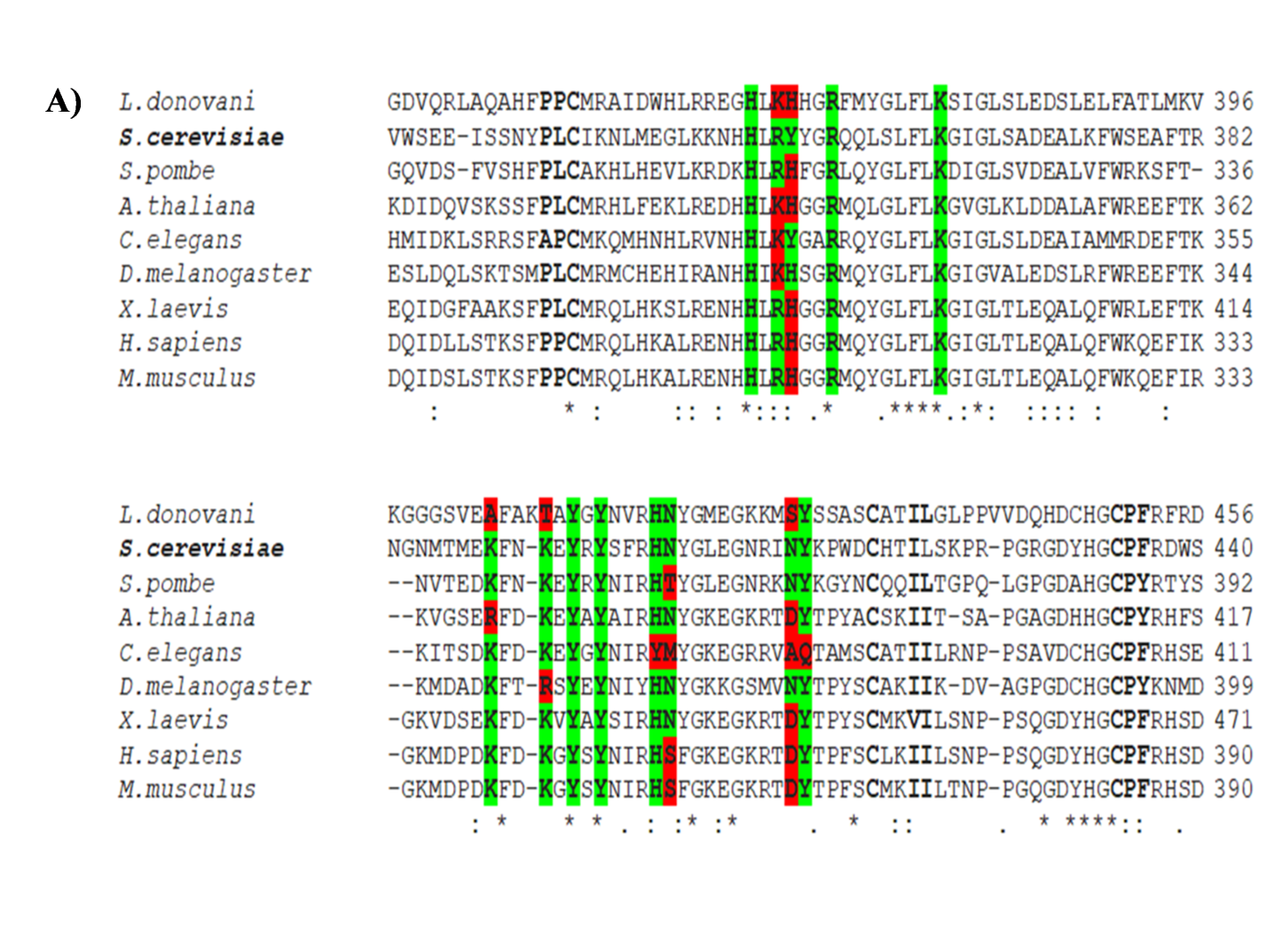


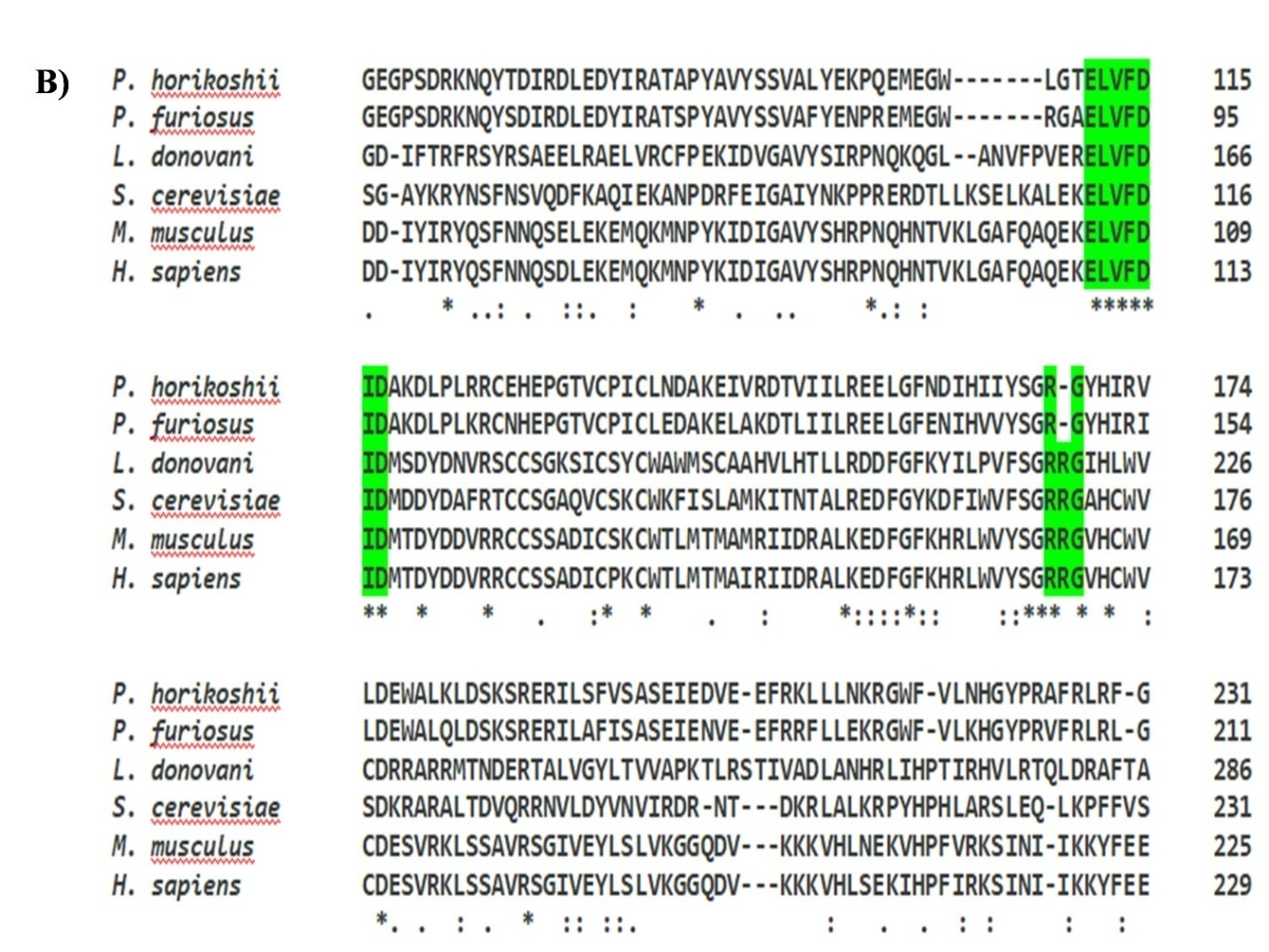


**Figure S1**. MSA by Clustal Omega for evaluating the conservation of residues (highlighted in green) and active site prediction in *L. donovani* DNA Primase **A)** large sub-unit (*LdPriL*) and **B)** small sub-unit (*LdPriS*)


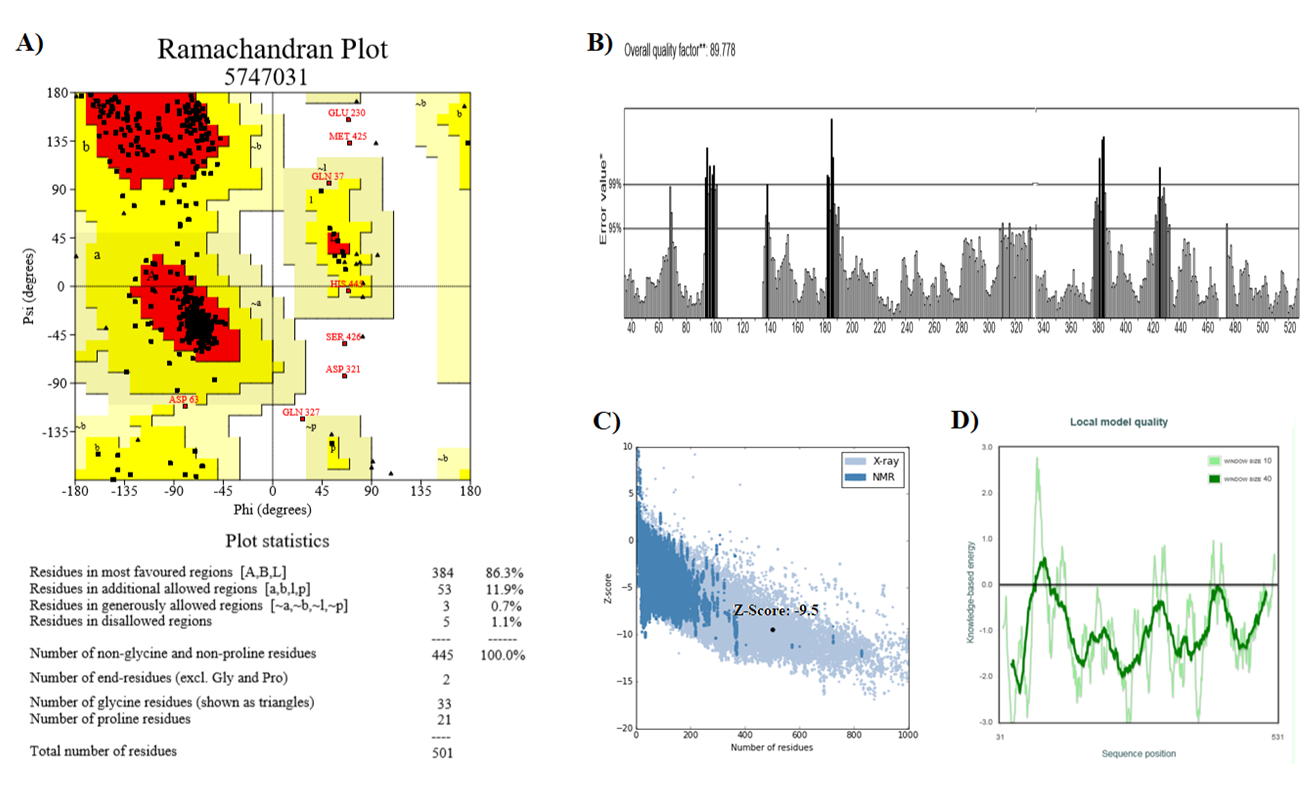


**Figure S2.** A) Ramachandaran plot of Model 2 for *LdPriL* modelling through PROCHECK, **B)** ERRAT plot for the *L. donovani* DNA Primase large sub-unit model. Black bars indicate distantly located misfolded region from the active site, gray bars demonstrate the error region between 95% and 99% and white bars indicate regions with lower rate protein folding, **C)**ProSA-web z-scores of all protein chains in PDB which are determined by X-ray crystallography (light blue) or NMR spectroscopy (dark blue) with respect to their length. The z-score of our target protein is -9.5 and is highlighted with black dot , D) ProSA-web energy plot of *L. donovani* DNA Primase large sub-unit model. Thick line demonstrates average energy over each 40 residue fragment. The thin line demonstrates the same with a smaller window size of 10 residues in the background of the plot.


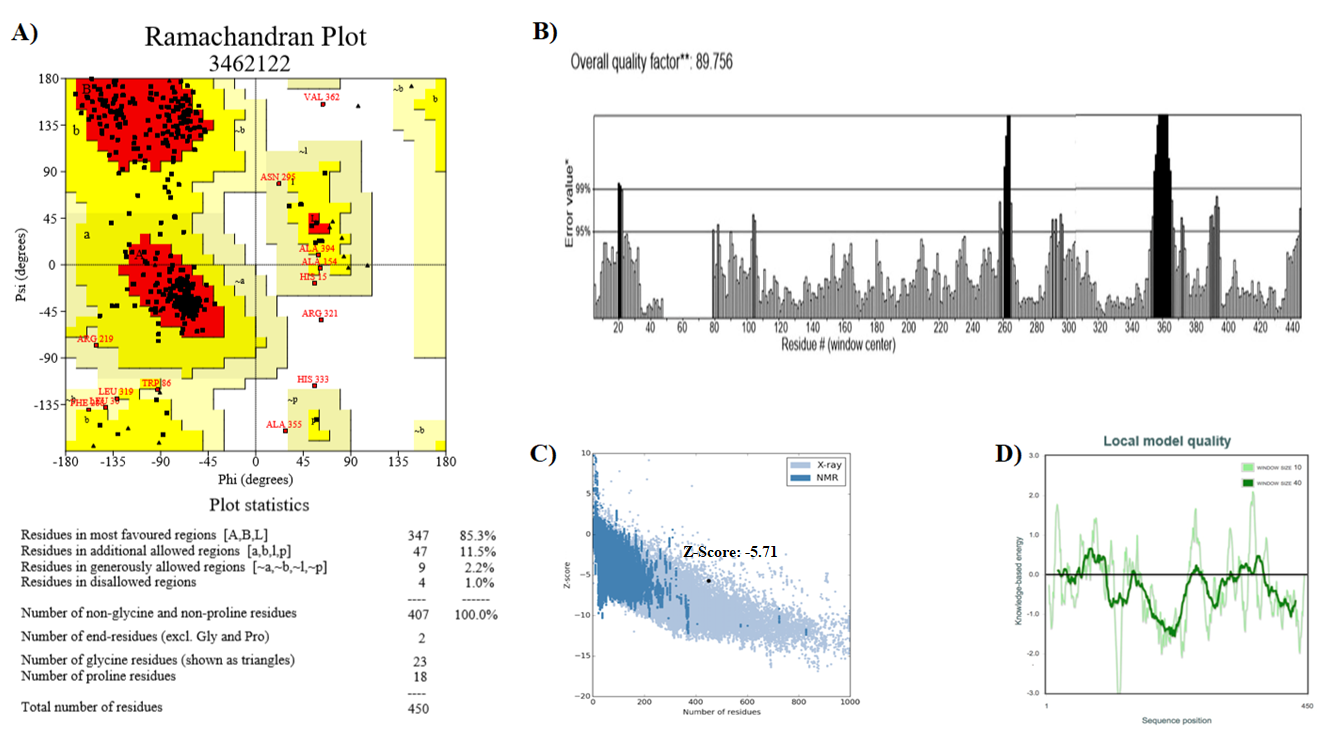


**Figure S3. A)** Ramachandaran plot of Model 4 for *LdPriS* modelling through PROCHECK, **B**) ERRAT plot for the *L. donovani* DNA Primase small sub-unit model. Black bars indicate distantly located misfolded region from the active site, gray bars demonstrate the error region between 95% and 99% and white bars indicate regions with lower rate protein folding, **C)** ProSA-web z-scores of all protein chains in PDB which are determined by X-ray crystallography (light blue) or NMR spectroscopy (dark blue) with respect to their length. The z-score of our target protein is -5.71 and is highlighted with black dot , **D)** ProSA-web energy plot of *L. donovani* DNA Primase small sub-unit model. Thick line demonstrates average energy over each 40 residue fragment. The thin line demonstrates the same with a smaller window size of 10 residues in the background of the plot.


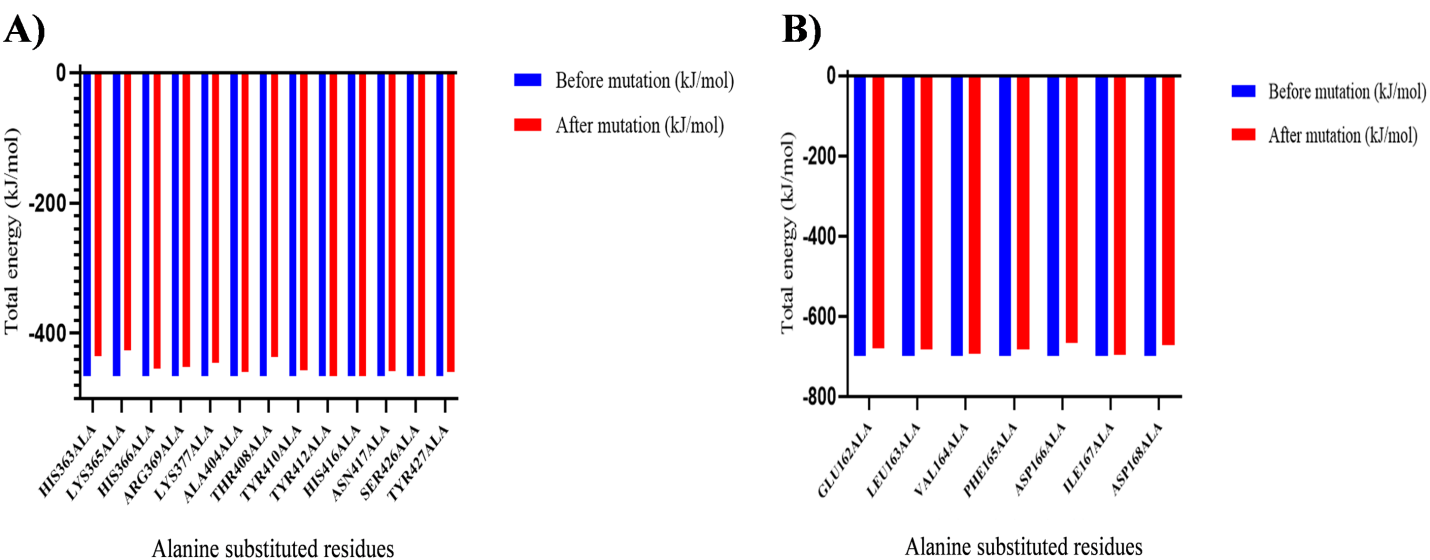


**Figure S4**. Bar diagram of Total energy difference of predicted active site residues after *In silico* Alanine Scanning Mutagenesis by PPCheck for **A)***LdPriL* by GraphPad prism vs. 8.0 and **B)***LdPriS* by GraphPad prism vs. 8.0.


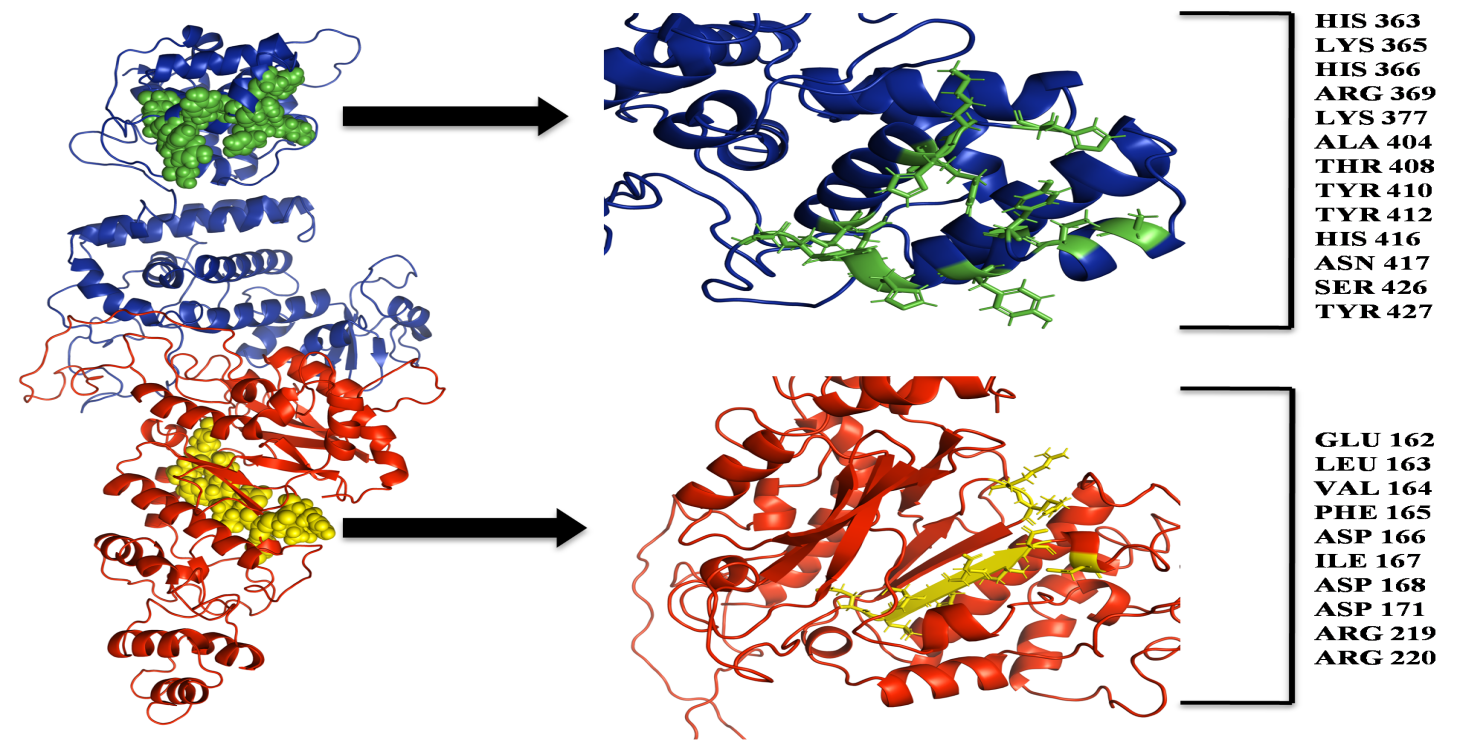


**Figure S5**. Diagrammatic reprsentation of the hotspot residues of *LdPriL* (blue and cartoon) and *LdPriS* (red and cartoon) in *LdPri* complex after *In silico* Alanine Scanning Mutagenesis selected for molecular docking. The hotspot residues of *LdPriL* are shown in green and sticks while hotspot residues of *LdPriS* are shown in yellow and sticks.


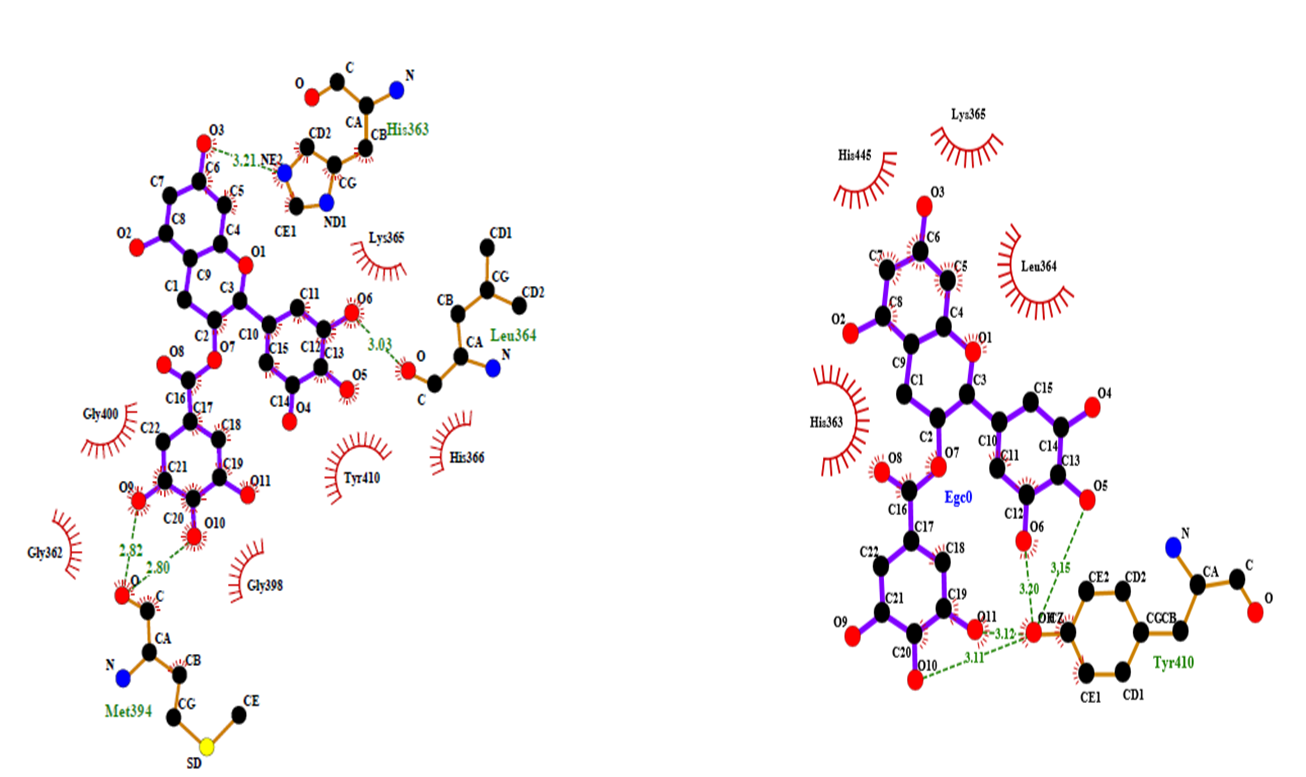
**Figure S6.**2D H-bond interactions of EGCG with *LdPriL* (left) and *LdPriL* in *LdPri* complex (right) by LigPlot+.


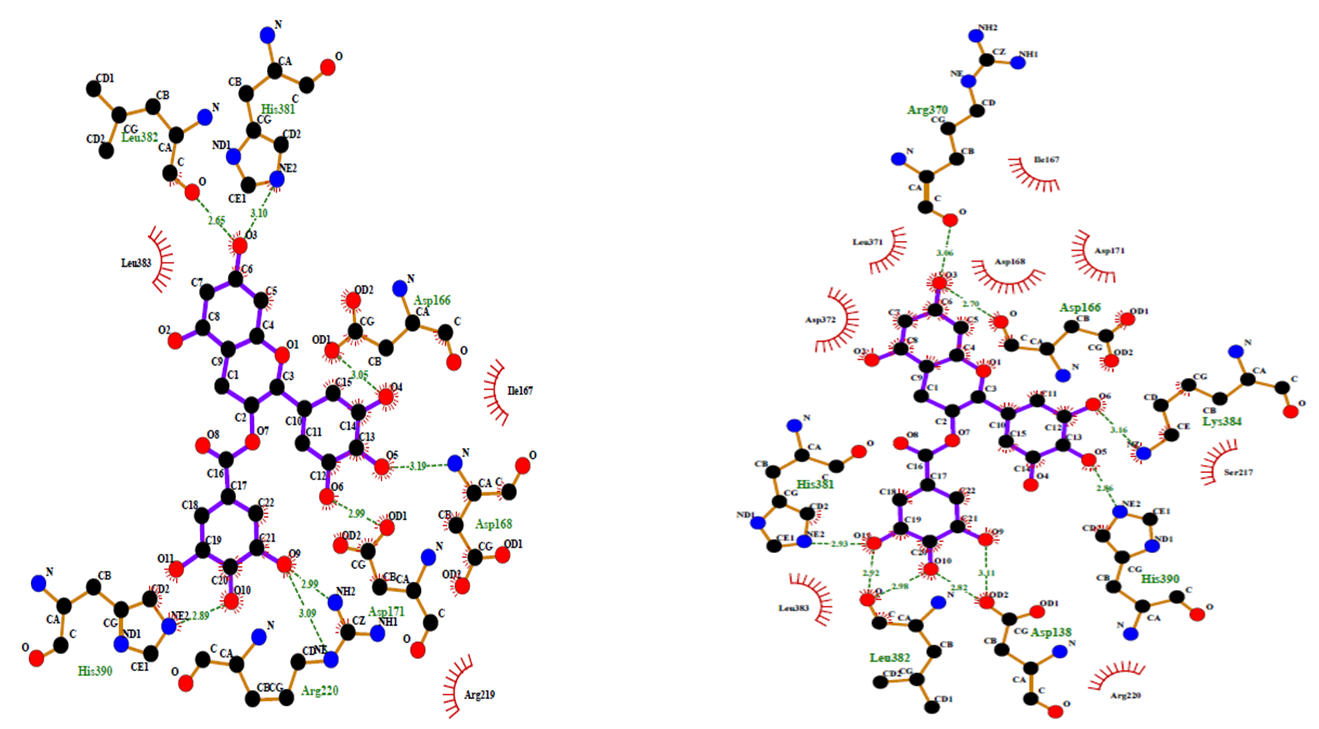


**Figure S7.** 2D H-bond interactions of EGCG with *LdPriS* (left) and *LdPriS* in *LdPri* complex (right) by LigPlot+.


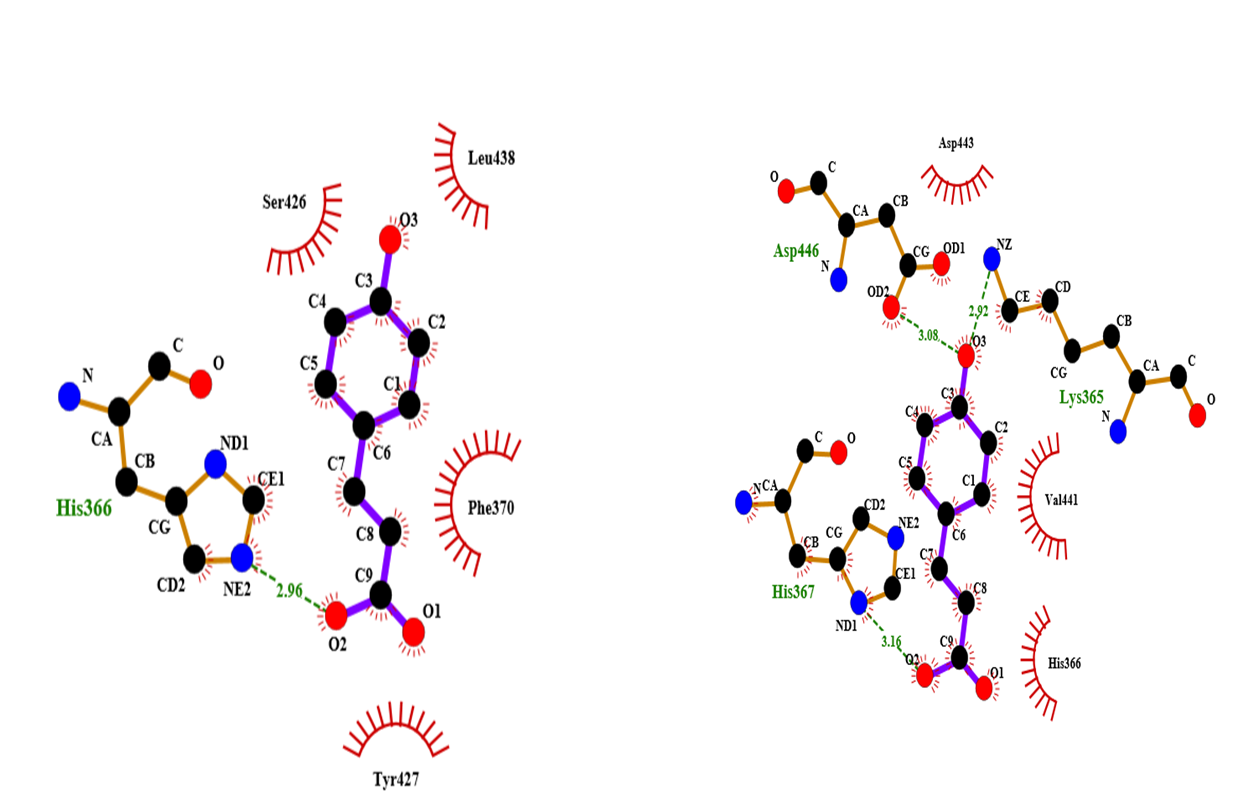


**Figure S8.**2D H-bond interactions of p-Coumaric acid with *LdPriL* (left) and *LdPriL* in *LdPri* complex (right) by LigPlot+.


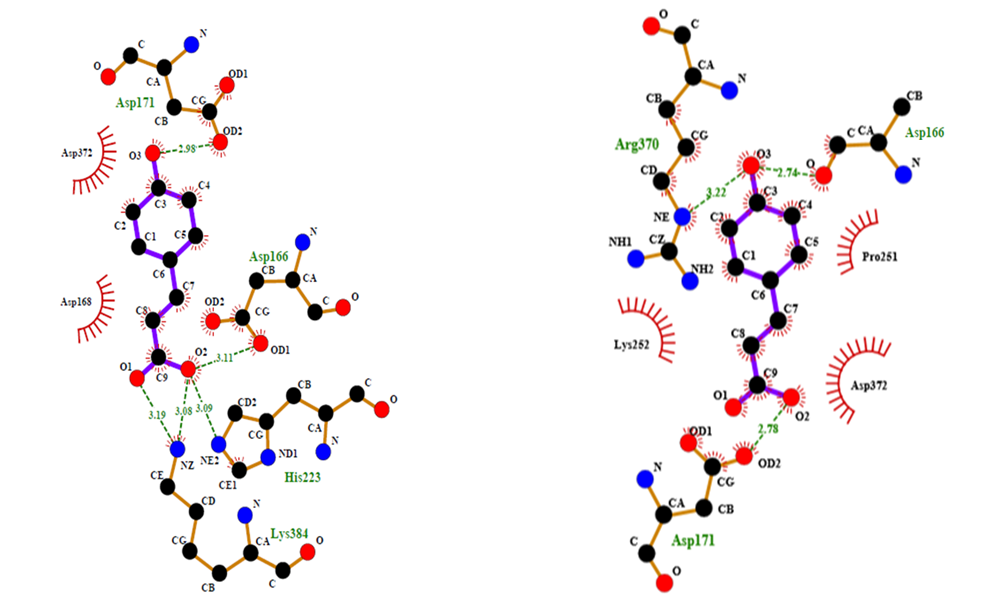


**Figure S9.** 2D H-bond interactions of p-Coumaric acid with *LdPriS* (left) and *LdPriS* in *LdPri* complex (right) by LigPlot+.


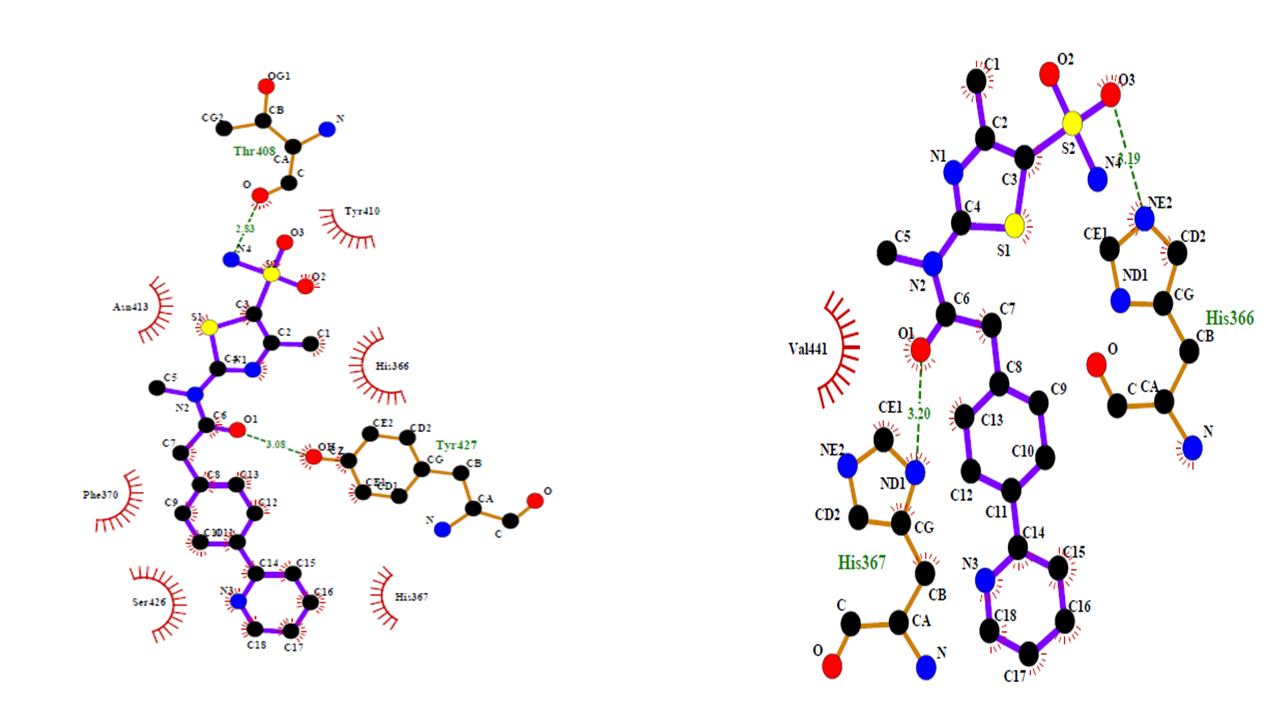


**Figure S10.** 2D H-bond interactions of Pritelivir with *LdPriL* (left) and *LdPriL* in *LdPri* complex (right) by LigPlot+.


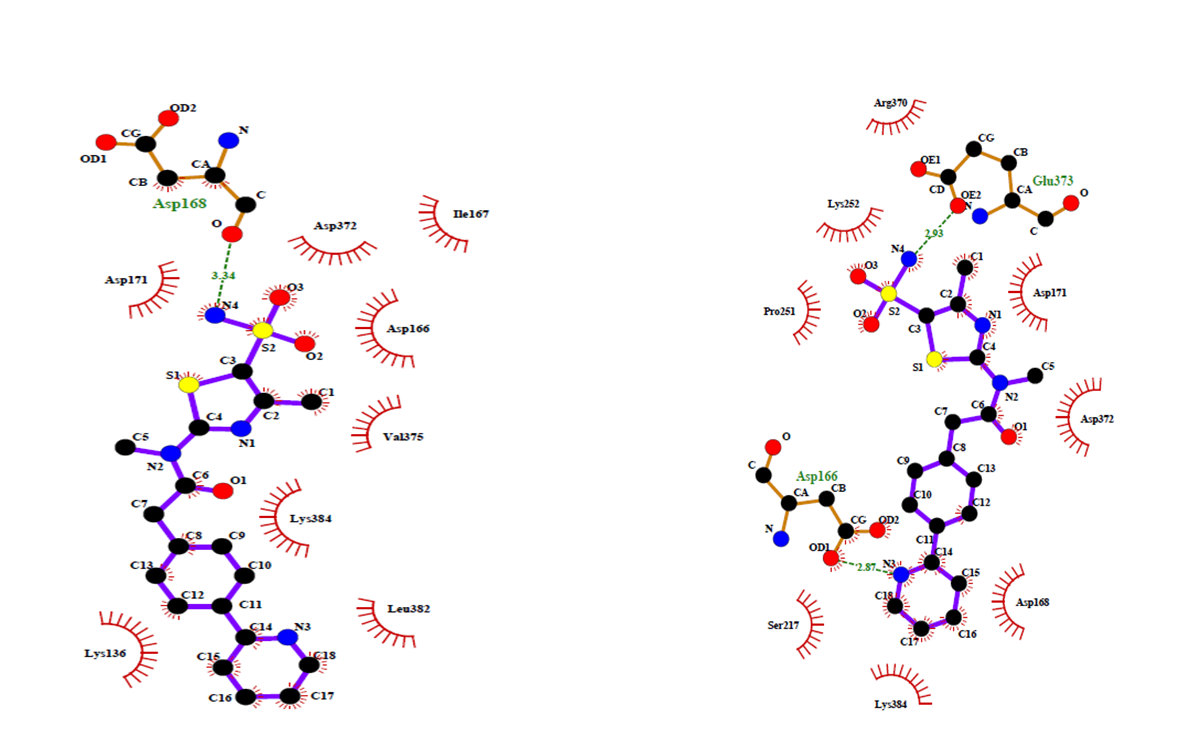


**Figure S11.** 2D H-bond interactions of Pritelivir with *LdPriS* (left) and *LdPriS* in *LdPri* complex (right) by LigPlot+.


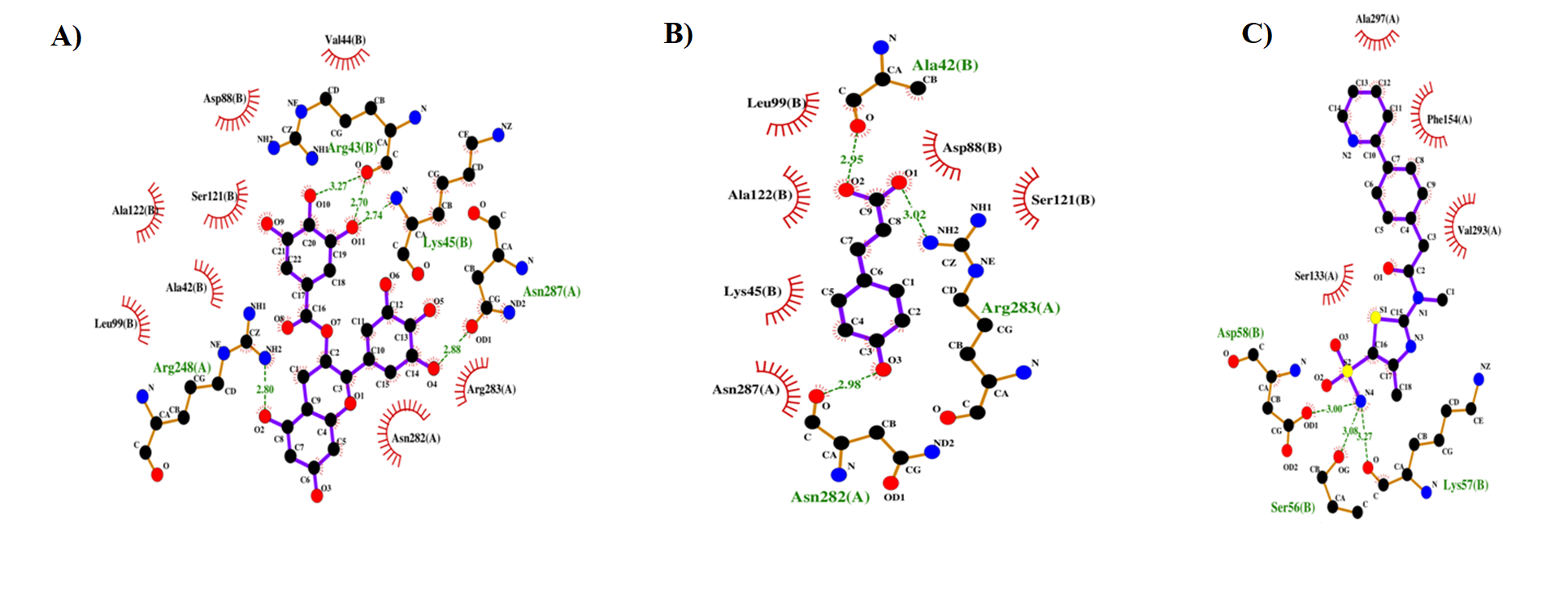


**Figure S12.** 2D H-bond interactions of A) EGCG , B) p-Coumaric acid and C) Pritelivir with interacting residues in *LdPri*complexbyLigPlot+.


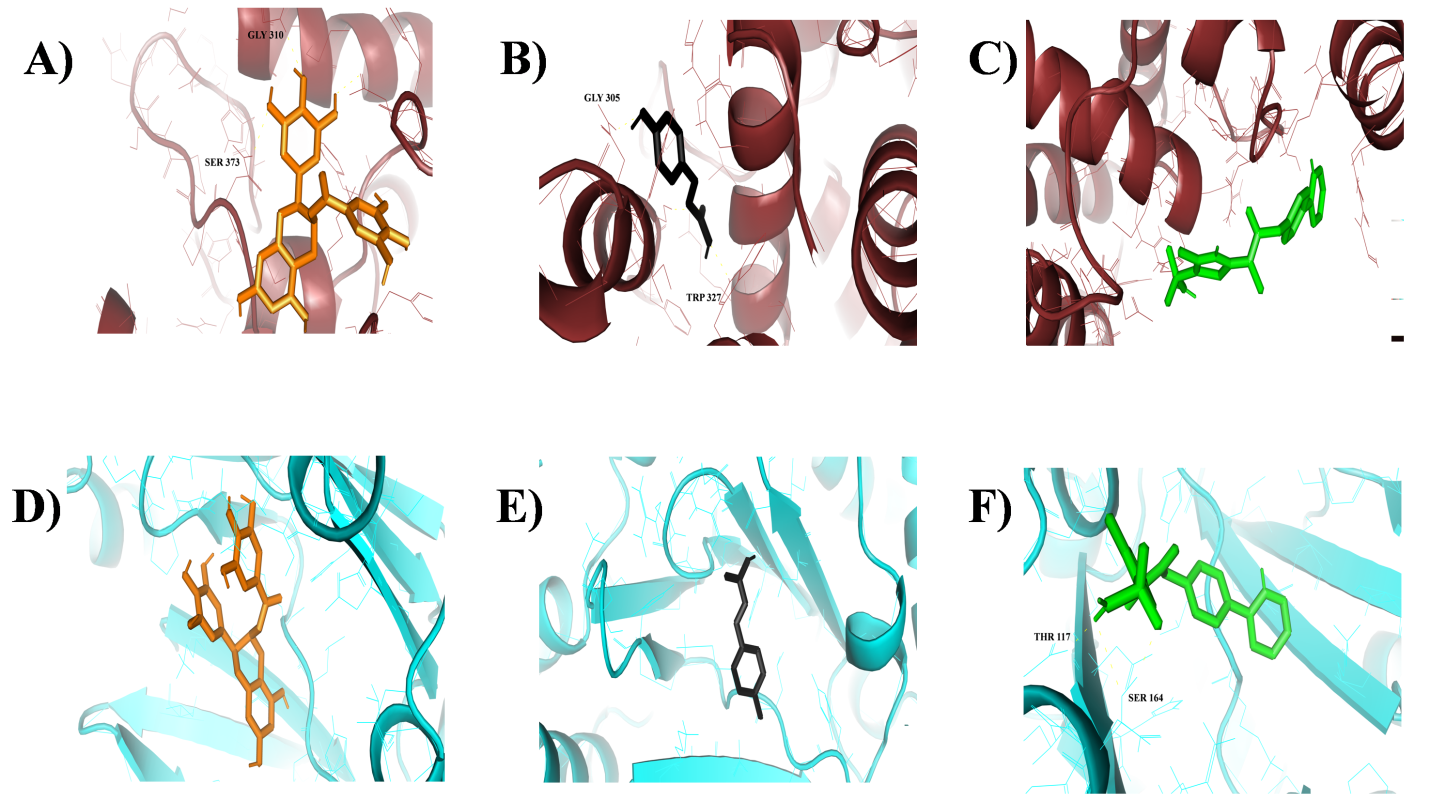


**Figure S13**. **A)** 3D representation of the H-bond interaction of EGCG (orange and sticks) with Human DNA Primase large subunit (ruby and cartoon), **B)** 3D representation of the H-bond interaction of p-Coumaric acid (black and sticks) with Human DNA Primase large subunit (ruby and cartoon), **C)** No H-bond interaction was observed for Pritelivir (green and sticks) against Human DNA Primase large subunit (ruby and cartoon), **D)** No H-bond interaction was observed for EGCG (orange and sticks) ) against Human DNA Primase small subunit (cyan and cartoon), **E)** No H-bond interaction was observed for p-Coumaric acid (black and sticks) against Human DNA Primase small subunit (cyan and cartoon) and **F)** 3D representation of the H-bond interaction of Pritelivir (green and sticks) against Human DNA Primase small subunit (cyan and cartoon).


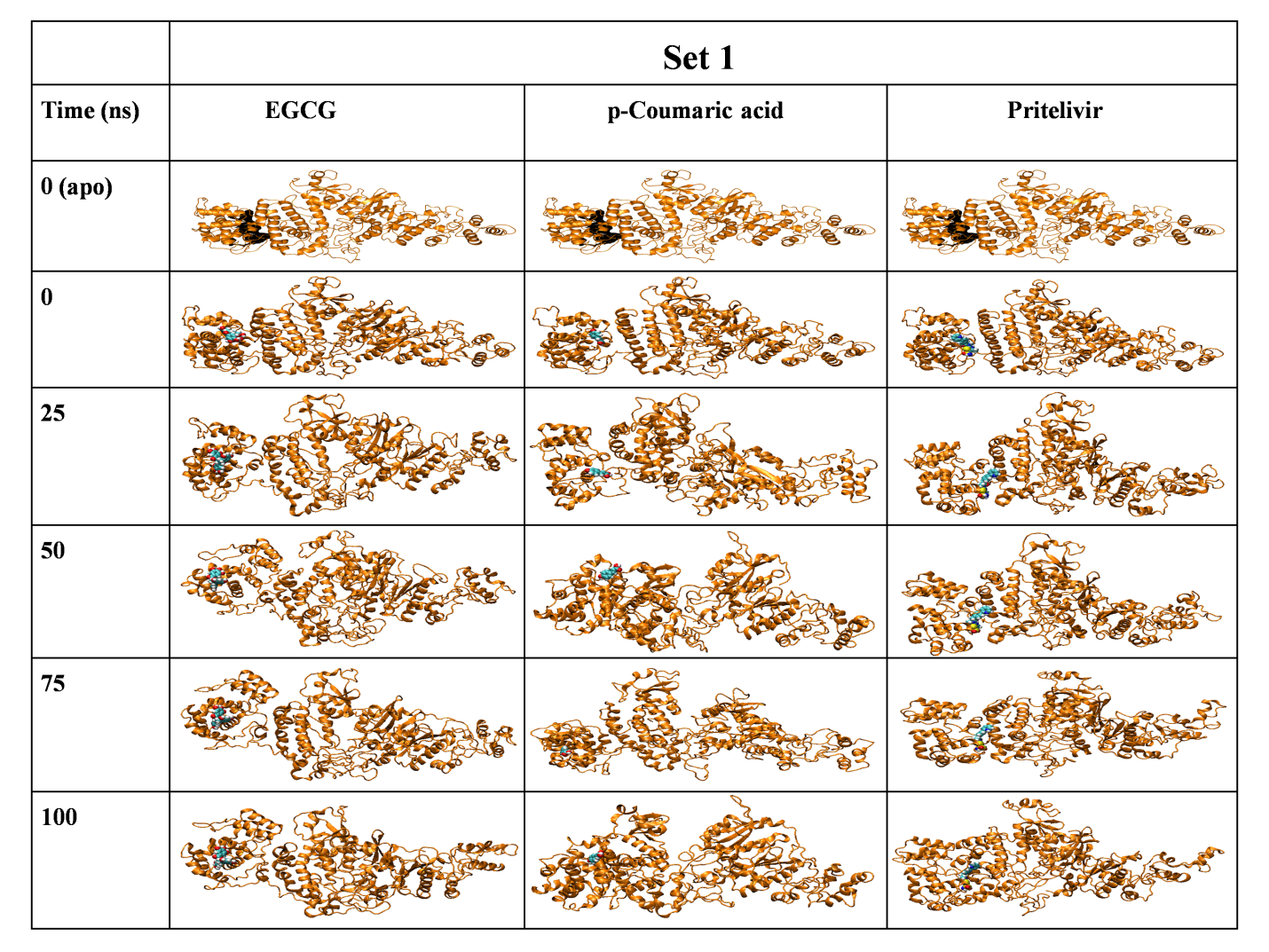
**Figure S14.**The stability and convergence ofEGCG, p-Coumaric acid and Pritelivir within the active site of *LdPriL* in *LdPri* complex in **Set 1**during **0ns, 25ns, 50ns, 75ns and 100ns** MD run along with the apo form showing the active site region (surface and black).


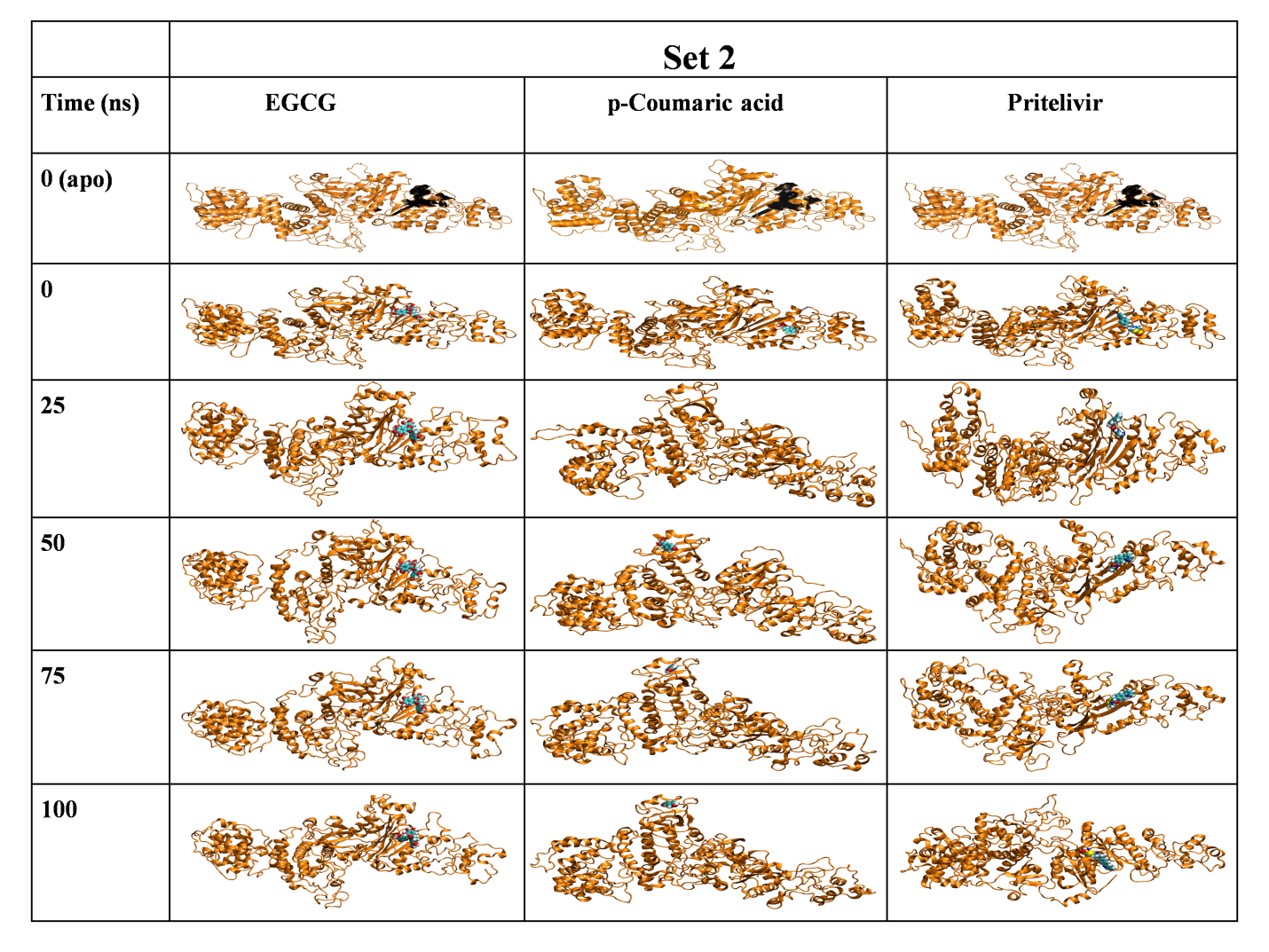
**Figure S15.**The stability and convergence ofEGCG, p-Coumaric acid and Pritelivir within the active site of *LdPriS* in *LdPri* complex in **Set 2**during **0ns, 25ns, 50ns, 75ns and 100ns** MD run along with the apo form showing the active site region (surface and black).

**
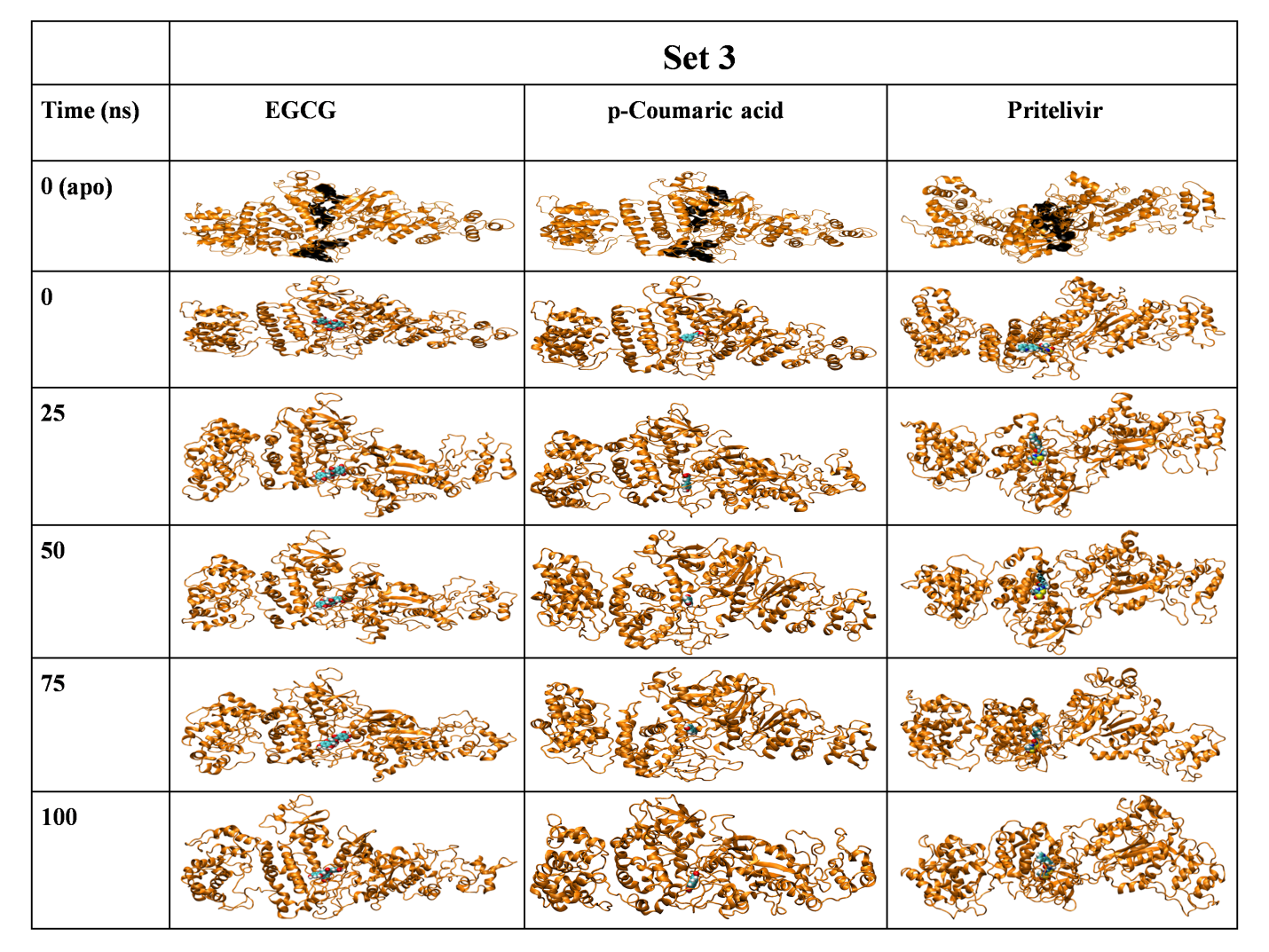
Figure S16.**The stability and convergence ofEGCG, p-Coumaric acid and Pritelivir within the interacting site between *LdPriL* and *LdPriS* in *LdPri* complex in **Set 3**during **0ns, 25ns, 50ns, 75ns and 100ns** MD run along with the apo form showing the active site region (surface and black).

**
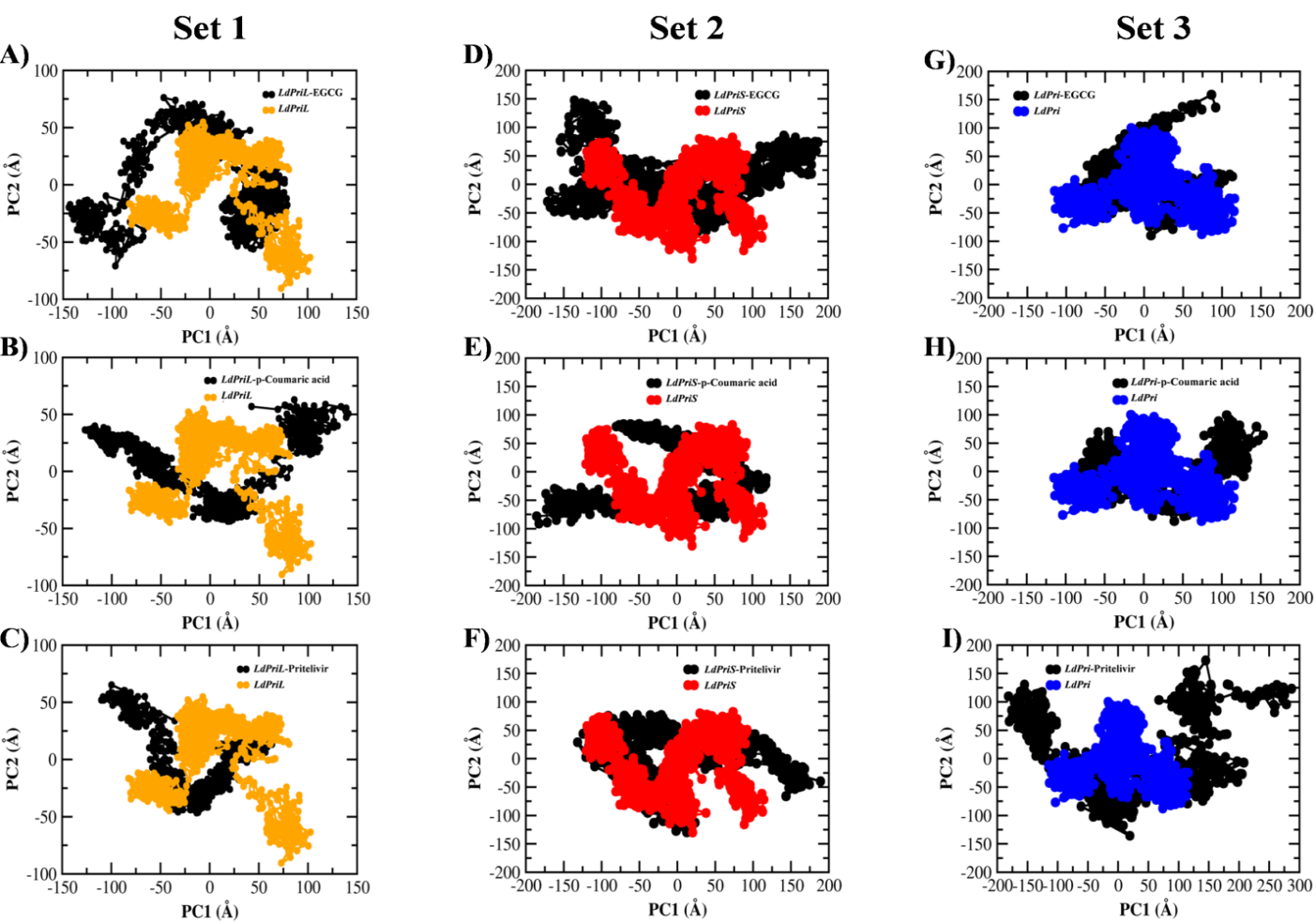
**

**Figure S17**. First two eigenvectors describing the projection of protein motion in phase space for EGCG, p-Coumaric acid and Pritelivir in **Set (1-3)**.

**
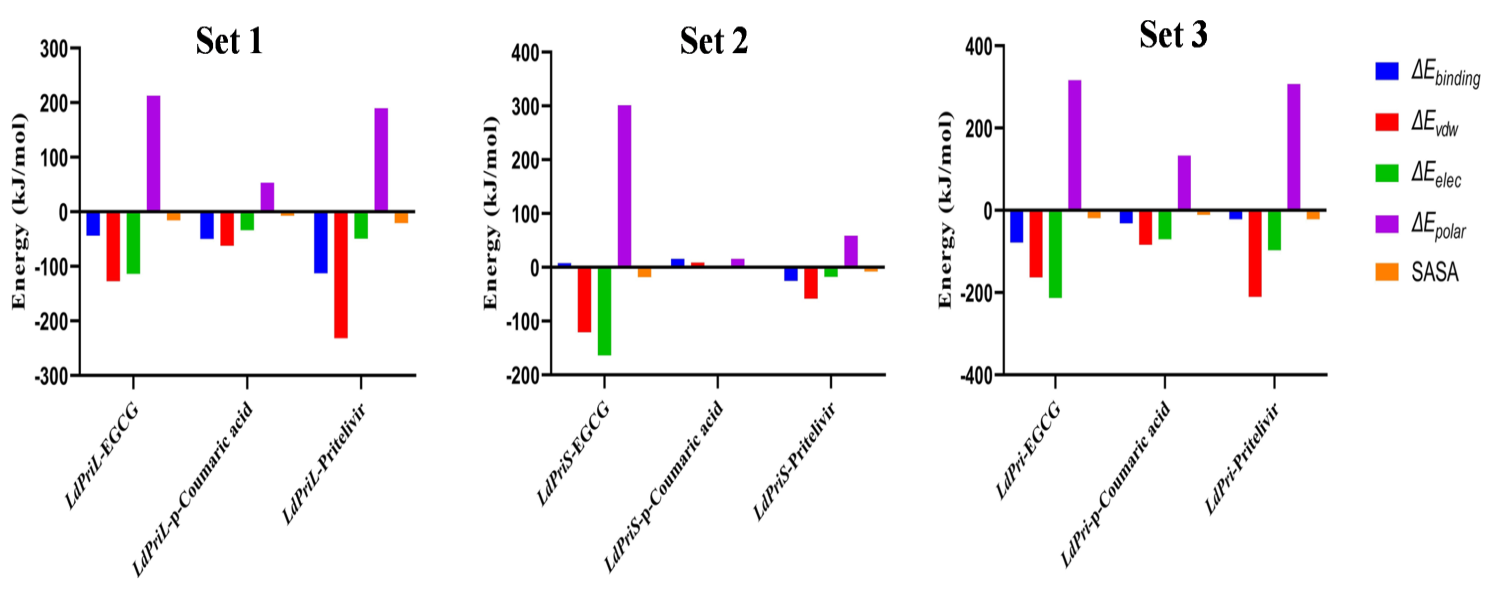
**

**Figure S18**. Comparison of different mmpbsa calculated energies evaluated for EGCG, p-Coumaric acid and Pritelivir against different hotspot sites in *LdPri* complex for **Set (1-3)**.

**
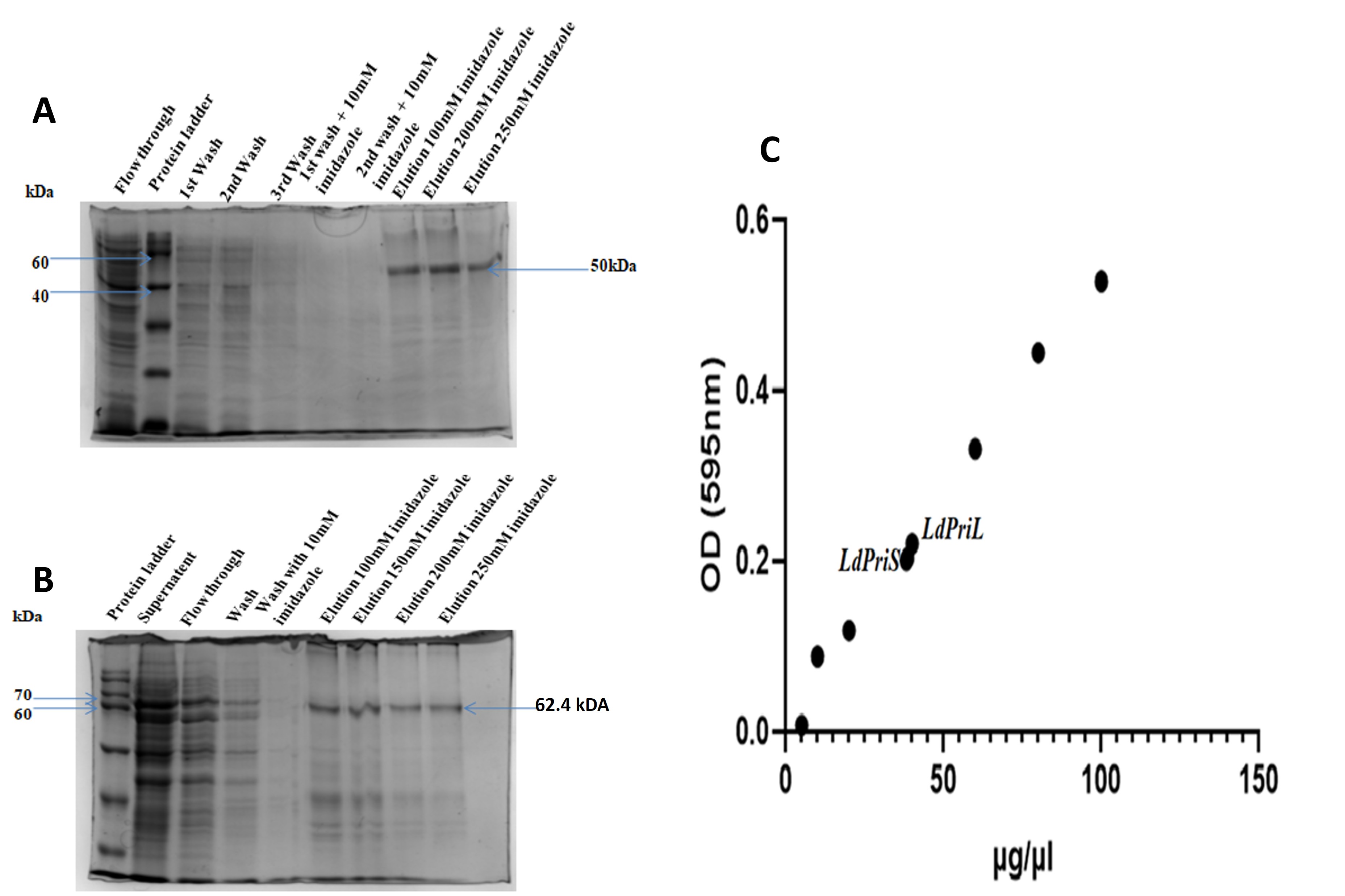
**

**Figure S19.** **A)** Purification of overexpressed LdpriS-pET-28a(+) by Co^2+^-NTA affinity chromatography. **B)** Purification of overexpressed LdpriL-pASK-IBA43plus by Co^2+^-NTA affinity chromatography, and **C)** Quantification of recombinant *LdPriL* and *LdPriS* by Bradford assay.

**
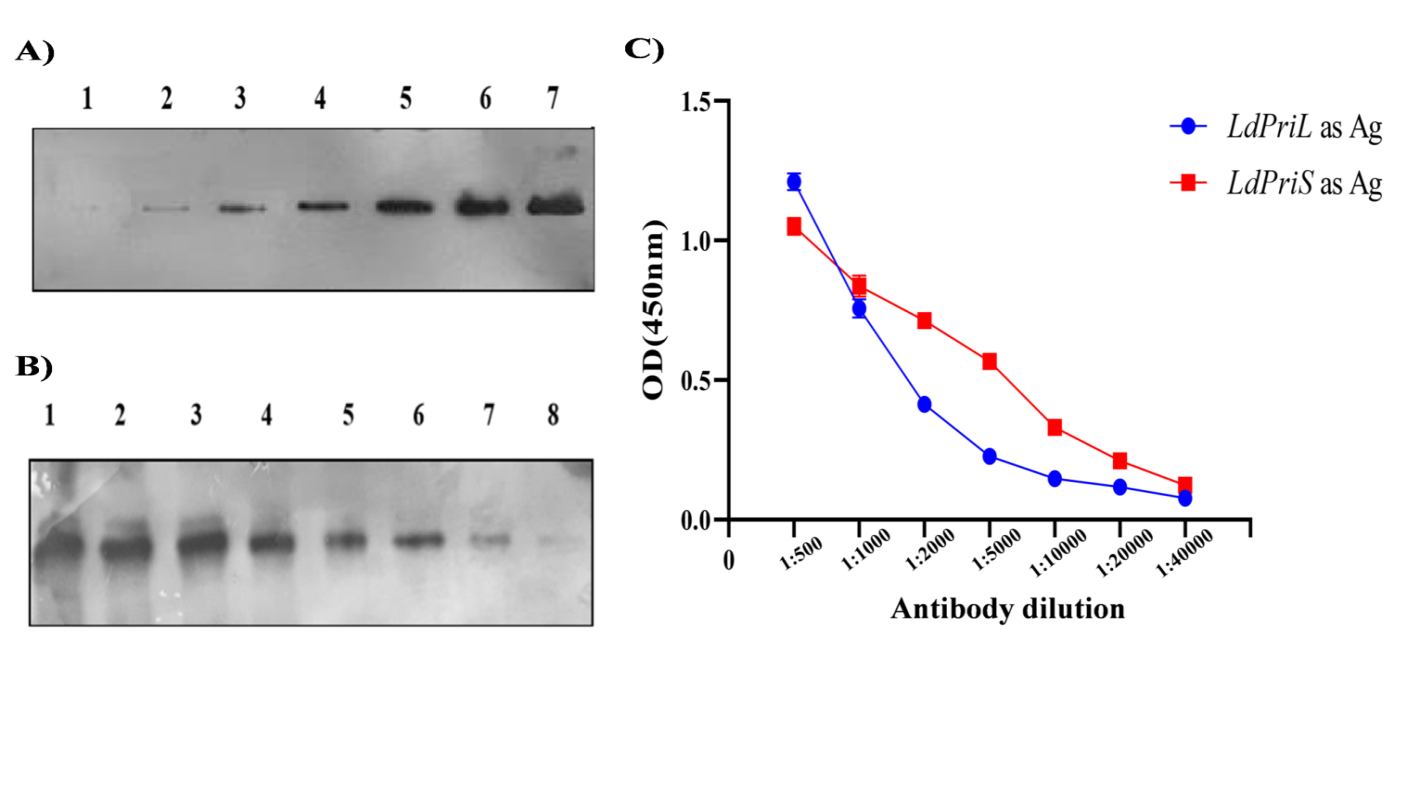
Figure S20.A)** Western blot analysis of 6x His-tagged Monoclonal antibody (NovagenR) using varying amount of purified recombinant *LdPriL* as Ag. Lane 1: 1 ng of Ag-protein, Lane 2: 5 ng of Ag-protein, Lane 3: 10 ng of Ag-protein, Lane 4: 20 ng of Ag-protein, Lane 5: 30 ng of Ag-protein, Lane 6: 50 ng of Ag-protein and Lane 7: 100 ng of Ag-protein; **B)** Western blot analysis of 6x His-tagged Monoclonal antibody (NovagenR) using varying amount of purified recombinant *LdPriS* as Ag. Lane 1: 100 ng of Ag-protein, Lane 2: 50 ng of Ag-protein, Lane 3: 30 ng of Ag-protein, Lane 4: 20 ng of Ag-protein, Lane 5: 10 ng of Ag-protein, Lane 6: 5 ng of Ag-protein and Lane 7: 1 ng of Ag-protein, Lane8: 0.5 ng of Ag-protein; **C)** Determination of titre of 6x His-tagged Monoclonal antibody by ELISA. The titre of 6x His-tagged Monoclonal antibody decreases with the increase of dilutions which suggests the purity of our recombinant *LdPriL* and *LdPriS*

**
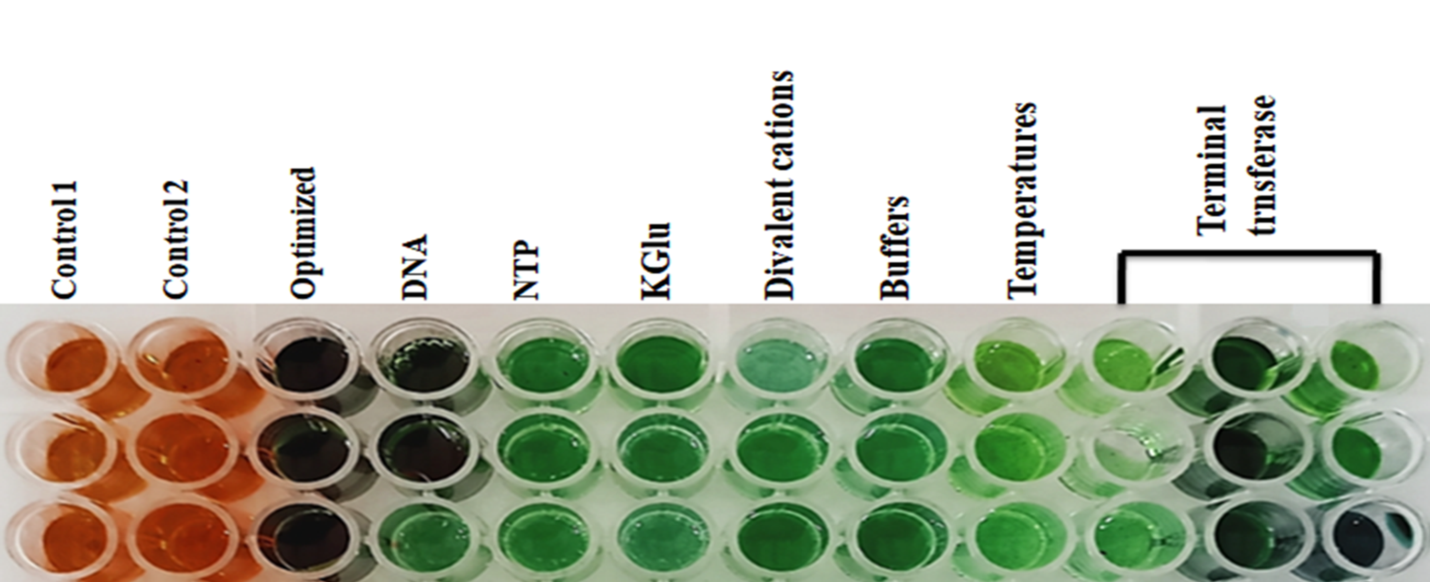
**

**Figure S21.** Optimization of *LdPri* activity by primase-pyrophosphatase assay in a 96-well microplate under different conditions and parameters.


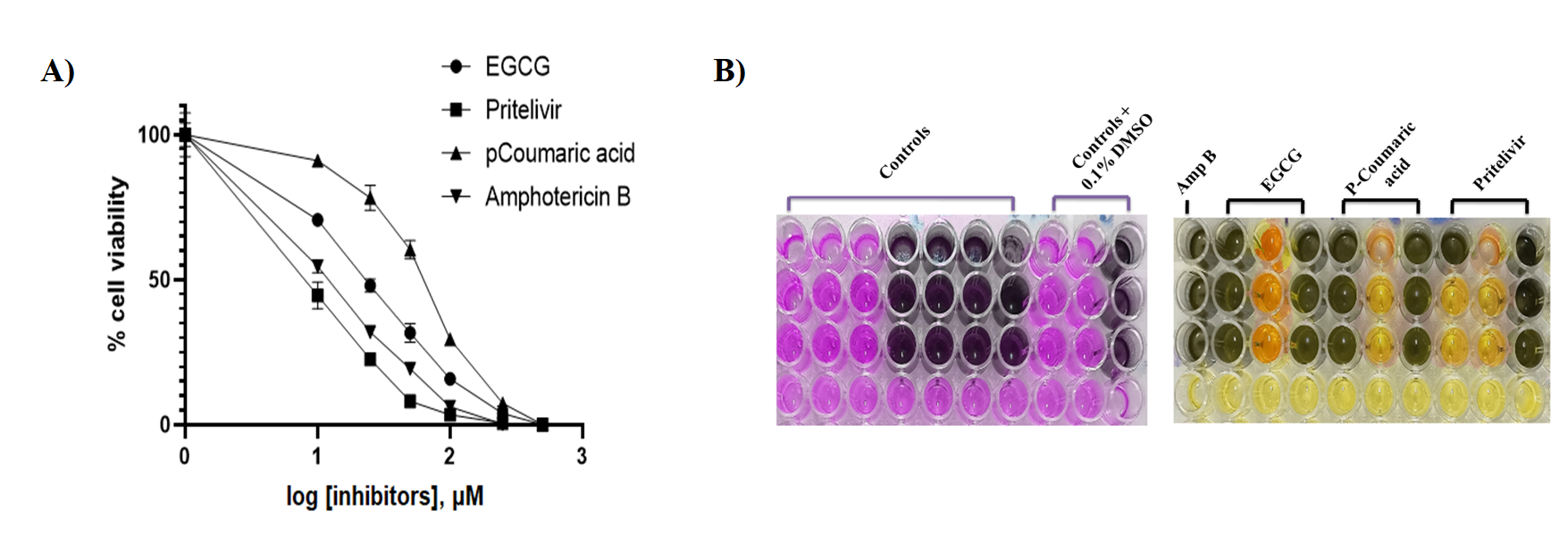


**Figure S22**. **A)** Dose-response plot for Amphotericin B, Pritelivir, EGCG and p-Coumaric acid from MTT assay studies in 96-well clear polystyrene plates and **B)** MTT assay in 96-well clear polystyrene plates evaluating *L. donovani* parasite inhibition at different concentrations of anti-leishmanials - Lane1 (right panel) : Amphotericin B (control) treated parasites; Lane 2, 3, 4 (right panel) : EGCG treated parasites; Lane 5, 6, 7 (right panel) : p-Coumaric acid treated parasites; Lane 8, 9, 10 (right panel) : Pritelivir treated parasites. Left panel : Controls and 0.1% DMSO treated cells with purple colour indicating no inhibition. All the experiments were done in triplicates. The bars indicate the standard deviation


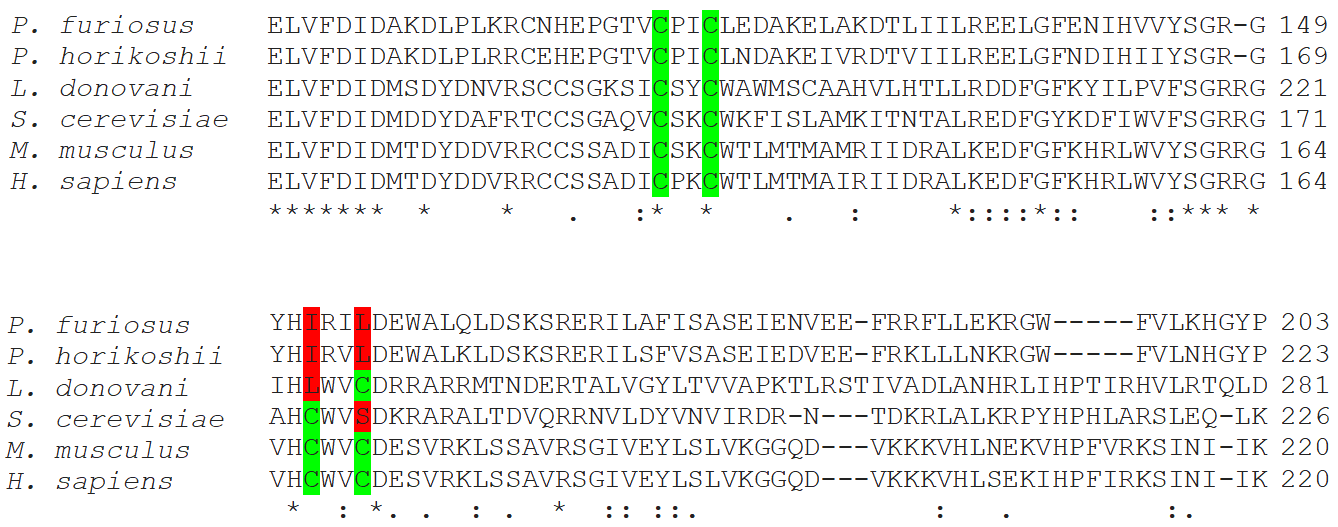


**Figure S23.** MSA by Clustal Omega for evaluating the conservation of Zn-binding motif residues in *L. donovani* DNA Primase small sub-unit (*LdPriS*). The conserved cysteine residues are highlighted in green. The penultimate cysteine residue was replaced by a leucine residue in *L. donovani* and is highlighted in red.


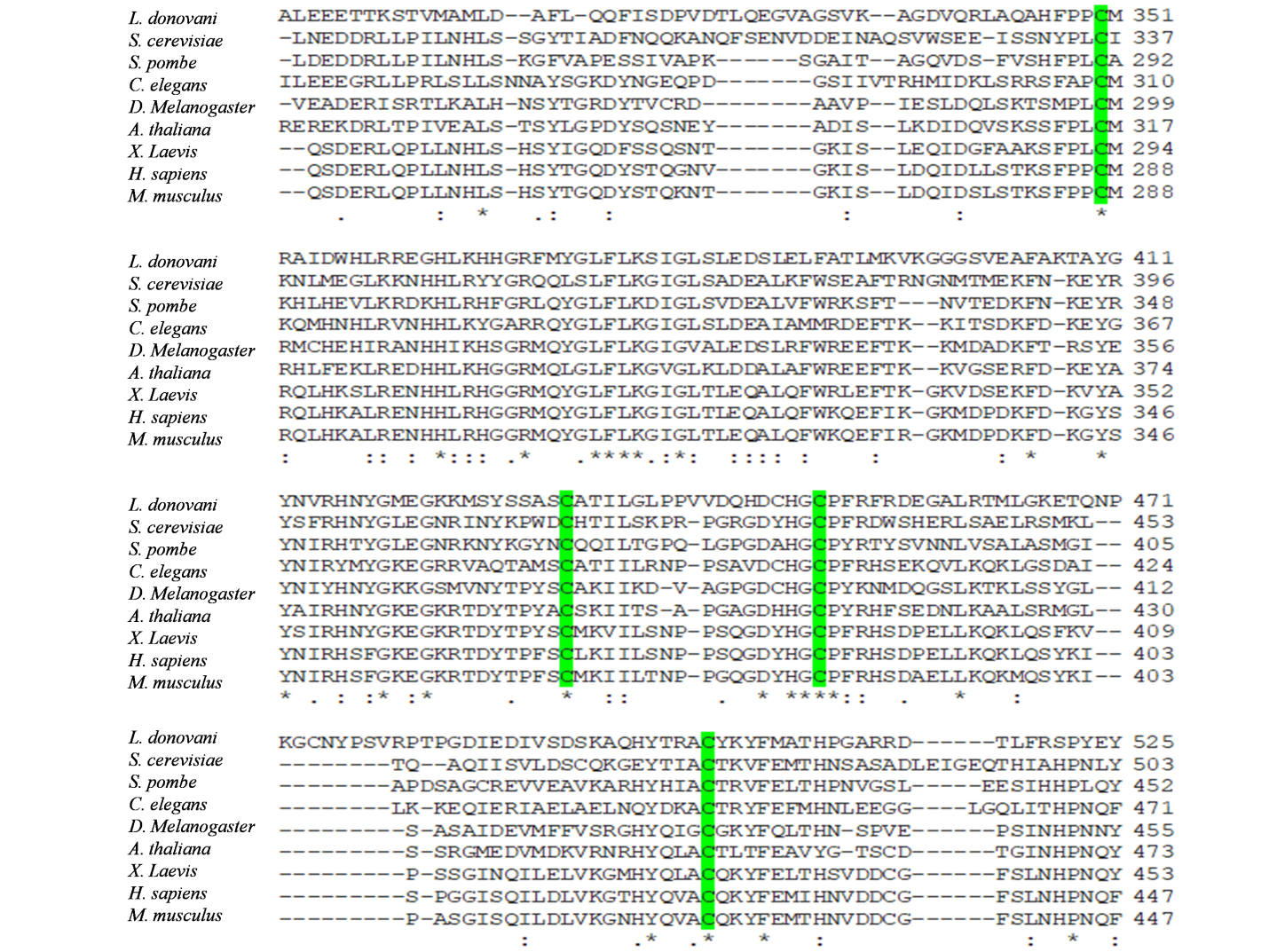


**Figure S24.** MSA by Clustal Omega for evaluating the conservation of Fe-S cluster in *L. donovani* DNA Primase large sub-unit (*LdPriL*). The conserved cysteine residues are highlighted in green.


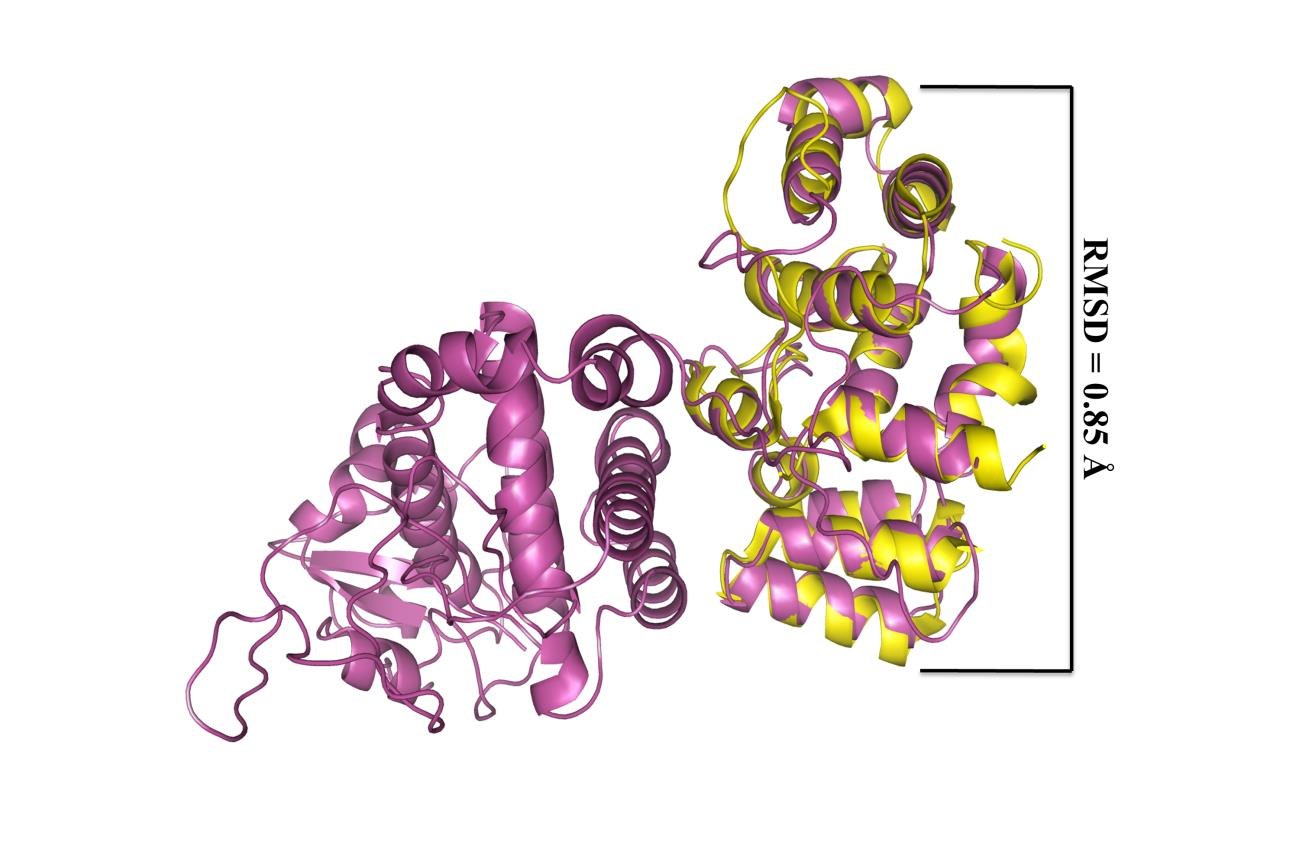
**Figure S25.** Protein-protein superimposition of *LdPriL*(pink) and *Crystal structure of* Fe-S Domain of the yeast DNA primase (PDB : 3LGB) (yellow) by Pymol software.

**Methods**

**Molecular Docking of the selected inhibitors with Human DNA Primase**

Molecular docking between Epigallocatechin Gallate (EGCG), p-Coumaric acid (4-Hydroxycinnamic acid) and Pritelivir (BAY 57-1293) was also done against both the large and small subunits of the energy minimized Human DNA Primase (PDB ID: 4RR2), through PyRx virtual screening software for docking purposes [**Dallakyan and Olson, 2015**]. The macromolecular structure of Human DNA Primase as well as the ligands, were prepared and hotspot residues were prepared inside a grid box for induced fit docking with X, Y and Z axis and dimensions adjusted to 29.25Å*×*35.51Å*×*26.55Å for large subunit and 24.62Å*×*20.33Å*×*24.31Å for small subunit of Human DNA Primase. The active sites for both the subunits of Human DNA Primase were selected based on the multiple sequence studies (**Figure S1)** and the conserved amino acids were considered. Moreover the conserved Fe-S cluster of DNA primase which is responsible for the initiation of replication through DNA charge transport on single-stranded DNA [**Klinge et al. 2007; Weiner et al. 2007; Vaithiyalingam et al. 2010; O'Brien et al. 2017**] was also selected from the multiple sequence study (**Figure S23).** The active site residues for both the subunits are tabulated in **Table S13.**

**Overexpression and purification of the LdpriL-pASK-IBA43plusand LdpriS-pET-28a (+) construct**

1. ***Growth and expression of recombinant E. coli cells*:** Transformation for positive clones for Ldpril-pASK-IBA43plusand Ldpris-pET-28a(+) into expression-competent *E.coli* BL21 cells were carried out for large-scale protein expression studies. As pASK-IBA43plus contains ampicillin resistant gene and pET-28a(+) containing that of kanamycin, a single colony of *E.coli* BL21 cells for the Ldpril-pASK-IBA43plus clone w*as* inoculated into 10 ml of LB medium containing ampicillin(100 mg/ml). In contrast, a single colony of *E. coli* BL21 cells for the Ldpris-pET-28a(+) clone were inoculated in an LB medium containing kanamycin (50 mg/ml). After overnight growth at 37ºC, these cultures were inoculated in 10 sets of fresh 100ml LB medium containing antibiotic ampicillin (100 mg/ml) and 10 sets with antibiotic kanamycin (50 mg/ml) for Ldpril-pASK-IBA43plusand Ldpris-pET-28a(+) respectively. When the cultures reached an optical density (OD) of ~(0.4-0.6) at 600 nm, culture was induced by the addition of anhydrotetracycline (final concentration of 200ng/ml) and isopropyl-β-D-thiogalactopyranoside (IPTG) (final concentration of 0.5 mM) for Ldpril-pASK-IBA43plusand Ldpris-pET-28a(+), respectively. Cells were grown overnight for both Ldpril-pASK-IBA43plusand Ldpris-pET-28a(+) at 16ºC for soluble protein, centrifuge at 10,000 rpm for 15 min at 4ºC and the pellet was stored at –20ºC for purification studies.
2. ***Solubility analysis of expressed protein***: To test the soluble nature of recombinant *LdPriL* and *LdPriS*, lysis buffer B (100 mM NaH_2_PO_4_, 10 mM Tris/HCl, 10 mM imidazole, pH 8.0) was used for suspending the pellet from the induced culture of Ldpril-pASK-IBA43plus and Ldpris-pET-28a(+). Both cultures have been collected and pelleted down in the same manner as earlier mentioned. The pellet was suspended again in 1 ml of buffer B and kept for 15 mins on ice. This lysate was mixed with 1 mg/ml lysozyme, then incubated for 30 mins on ice, sonicated and centrifuged at 4°C for 30 mins at 10,000 rpm. The supernatant was kept in a separate tube while the suspension of the pellet in lysis buffer B was carried out for solubility analysis by SDS-PAGE.
3. ***Single step purification by affinity chromatography*:**100 ml cell pellet for both the LdpriL-pASK-IBA43plus and LdpriS-pET-28a(+) constructs were thawed on ice for 15 mins and then resuspended in 5ml lysis buffer (50 mM NaH_2_PO_4_, 300 mM NaCl, 10 mM imidazole; pH 8.0) for each 100ml pellet. The suspension was added with 1 mg/ml lysozyme at a final concentration alongside cocktail protease inhibitor (1X) and 0.5mM EDTA and was further incubated for 30 min on ice. The suspension was sonicated for cell lysis and cleared lysate formation, was centrifuged at 10,000 *g* for 30 min and again at 14,000 rpm for 5 min at 4ºC to pellet down the cell remains, and collected supernatant only. The supernatant for both the constructs was transferred to a gravity flow column charged with 100μl TALON® Metal Affinity Resin (Takara) containing Co^2+^ ions specific for His-tagged purification (higher specificity than nickel-charged resins) and equilibrated with lysis buffer. Initially, the column was washed with wash buffer alone before being treated with imidazole (50 mM NaH_2_PO_4_, 300 mM NaCl, 20 mM imidazole; pH 8.0), and finally, elution was completed by using elution buffer (50 mM NaH_2_PO_4_, 300 mM NaCl, 250 mM imidazole; pH 8.0). The imidazole concentration was gradually increased to 500 mM for a complete pool down of the His-tagged protein from the charged metal resins and desalted using 10K MWCO Pierce™ Protein Concentrator (Thermo Scientific™). Finally, *LdPriL* and *LdPriS* purified protein was quantified using Bradford protein estimation [Kruger, 1994]. 100 μl aliquots of each purified protein in 20% glycerol were stored at -80°C for future use for primase activity and inhibition assays.

**Optimization of colorimetric primase-pyrophosphatase assay conditions and Biochemical characterization of *LdPri***

Most academic screening facilities are limited to experimenting with radioactive elements; a novel non-radioactive primase-pyrophosphatase (also primase-phosphatase) assay with high throughput screening (HTS) applications reported earlier provided a new ready-to-use approach [**Biswas et al., 2013**]. The primase activity assay was optimized on flat-bottom 96-well clear polystyrene plates (Thermo Scientific™). 30 μl of reaction mixture contained *LdPri* (0.7 µM both *LdPriL* and *LdPriS* in 1:1 molar ratio), M13mp18 ss DNA (1.25μM or specified), dNTPs (100μM or as specified), buffer (20mM of CAPS pH 8.8 or specified), divalent metal ions (4 mM Mg^2+^ or as specified), 50mM NaCl, potassium glutamate (KGlu) (150 mM or as specified) and 1 U pyrophosphatase (PPiase). Reactions were carried out at 22ºC in an incubator for 30mins. After incubating addition of 90μl malachite green reagent (MGR) followed by 30μl of 10% sodium citrate was added after 1min to end the reaction, and the absorbance at 620 nm was taken [**Biswas et al., 2013; Rai et al., 2021**] in a microplate reader. Primase-pyrophosphatase assay for *LdPriL* and *LdPriS* was also considered to measure their primase activity individually.
